# Occupational psychosocial exposures and chronic low-back pain: a systematic review and meta-analysis

**DOI:** 10.5271/sjweh.4165

**Published:** 2024-07-01

**Authors:** Alexander Jahn, Johan Hviid Andersen, Andreas Seidler, David Høyrup Christiansen, Annett Dalbøge

**Affiliations:** 1 Danish Ramazzini Centre, Department of Occupational Medicine, Aarhus University Hospital, Aarhus, Denmark.; 2 Department of Clinical Medicine, Aarhus University, Aarhus, Denmark.; 3 Department of Occupational Medicine - University Research Clinic, Danish Ramazzini Centre, Goedstrup Hospital, Herning, Denmark.; 4 Institute and Policlinic of Occupational and Social Medicine (IPAS), Faculty of Medicine, Technische Universität Dresden, Fetscherstr, 74, 01307 Dresden, Germany.; 5 Elective Surgery Centre, Silkeborg Regional Hospital, Silkeborg, Denmark.; 6 Centre of Health and Nursing Research, Regional Hospital Central Jutland, Viborg, Denmark.

**Keywords:** etiology, job demand, spine, stress, work

## Abstract

**Objective:**

This study aimed to explore the association between occupational psychosocial exposures and chronic low-back pain (LBP) by conducting a systematic review and meta-analysis.

**Methods:**

The research protocol was registered in PROSPERO. A systematic literature search was performed in six databases, identifying articles complying with predefined inclusion criteria. In our PECOS, we defined outcome as chronic LBP ≥3 months, exposures as occupational psychosocial exposures, and restricted study design to case–control and cohort studies. Two authors independently excluded articles, extracted data, assessed risk of bias, and graded evidence levels. Meta-analyses were performed using random-effects models.

**Results:**

The 20 included articles encompassed six different occupational psychosocial exposures (job control, demand, strain, support, stress, and satisfaction), only 1 had low risk of bias. For all occupational psychosocial exposures, odds ratios ranged from 0.8 to 1.1. Sensitivity analyses based on risk of bias was conducted for two outcomes ie, job control and job demand, finding no differences between high and low-to-moderate risk of bias studies. Using GRADE, we found a very low level of evidence of the association for all occupational psychosocial exposures.

**Conclusion:**

In this study, we found no association between occupational psychosocial exposures and chronic LBP. However, it is important to underline that the level of evidence was very low. High quality studies are highly warranted.

Low-back pain (LBP) constitutes one of the leading causes of years lived with functional limitations and work absenteeism (1). Risk factors associated with the development of LBP, including chronic LBP, have been investigated extensively. It is acknowledged that potential risk factors encompass both individual factors (1), occupational and non-occupational mechanical (2–5) and psychosocial exposures (6).

Occupational psychosocial exposures are a complex interplay, and gaining insight in understanding the pathways and profound implications on the development of chronic LBP affecting public health and well-being is crucial. In 1979, Karasek proposed the job strain model, a theoretical framework explaining job strain as a two-dimensional concept resulting from the interplay between job demands and job control (7). Expanding on this framework, Bongers and colleagues integrated former research traditions to develop a concept illustrating the relationship between occupational psychosocial exposures and onset of musculoskeletal symptoms (8, 9). In short, occupational psychosocial exposures might trigger psychological reactions, including biomechanical processes, potentially leading to eg, muscle tension, inhibited repair of muscle tissue, or perception of symptoms (10, 11). However, this interplay contributes to the complexity influencing potential associations with various health outcomes and raises questions about if and how psychosocial factors independently affect, for instance, musculoskeletal symptoms.

Recent studies have identified emerging occupational psychosocial exposures beyond job strain, expanding the research area considerably. Notably, the construct of an organization and management has gained attention to exposures such as overtime work, organizational injustice, work-family imbalance, job insecurity, job satisfaction, etc (6). Despite this attention towards occupational psychosocial exposures as a risk factor for the development of LBP, conflicting evidence arises. In a systematic review, Hartvigsen et al (12) found moderate evidence for no association between perception of work, organizational aspects of work, and social support at work and LPB, while insufficient evidence of a positive association between stress at work and LBP was found. In 2014, the very comprehensive ‘Statens beredning för medicinsk utvärdering’ (SBU) concluded that moderate evidence of an association was found between job control [odds ratio (OR) 1.17, 95% confidence interval (CI) 1.02–1.34] and work satisfaction (OR 1.29, 95% CI 1.18–1.42) and general back problems (13). In addition, Niedhammer et al's meta-review (6) resulted in small but significant findings. Based on two systematic reviews, it found an association between LBP and, respectively, job strain [relative risk (RR) 1.40 and 1.38], low decision latitude (RR 1.37 and 1.30), job demands (RR 1.34 and 1.32), social support (RR 1.22 and 1.42), and insecurity (RR 0.85 and 1.43).

Only one systematic review has been conducted assessing the association between occupational psychosocial exposures and chronic LBP defined as persisting pain for >3 months. In 2019, Buruck et al (14) included 18 epidemiological studies and found that workload (OR 1.32, 95% CI 1.20–1.46) was associated with chronic LBP. Conversely, high job control (OR 0.81, 95% CI 0.71–0.94) and high social support (OR 0.77, 95% CI 0.65–0.90) were significantly related to protection of developing chronic LBP. This systematic review included studies with less strict definition of chronic LBP. In this review, approximately 50% of the included studies were cross-sectional in design with no temporality between exposure and outcome. Therefore, the purpose of this systematic review and meta-analysis was to summarize the epidemiological evidence of the association between occupational psychosocial exposures and the development of chronic LBP strictly adhering to pain persisting in >3 months based on cohort and case–control studies.

## Methods

The systematic review and meta-analysis was conducted in accordance with guidelines stated by the PRISMA (Preferred Reporting Items for Systematic Reviews and Meta-Analyses) 2015 checklist. The study was registered in PROSPERO (the International Prospective Register of Systematic Reviews) with registration number CRD42021281996. No ethical approval was needed since the systematic review and meta-analysis is based upon published data.

### Search strategy

Due to the expected comprehensive literature, we decided to conduct the literature search in two steps. First, articles were retrieved from the SBU report, containing articles published from 1980 to 10 January 2014 (13). Secondly, concerning articles published after 10 January 2014, a systematic literature search was designed, tested, and performed in accordance with a librarian. The search string was developed identically to the SBU literature search. We performed the literature search between 2–21 September 2021 and updated it on 28 September 2022 using the following international electronic databases: PubMed, EMBASE, PsycINFO, CINAHL, Cochrane Library, and Web of Science (supplementary material, www.sjweh.fi/article/4165, Appendix A, table S1). In addition, the first 100 articles that emerged in a Google Scholar search were screened, and bibliographies of the included articles were hand-searched. Afterward, two of the authors selected the relevant articles and independently screened all articles using a two-step model, using the Covidence systematic review software. Firstly, articles were screened based on title and abstract. Secondly, articles were screened based on full-text reading. A third review author resolved any disagreements between the two review authors.

### Eligibility criteria

The systematic literature was based on criteria incorporated in a PECOS (Population, Exposure, Comparison, Outcome, and Study Design) (supplementary Appendix B, table S2). We restricted the population to adults of or above the working age. The exposure was defined as occupational psychosocial exposures with no limitations regarding the exposure assessment. Articles were excluded if solely based on job titles. For comparison, groups should consist of exposed versus non/low exposed and quantified using an appropriate measure of association [OR, RR, prevalence ratio (PR), or hazard ratio (HR)], or a measure that could be calculated. The outcome was defined as persisting LBP for >3 months (15), including specific (ie, sciatica, lumbar disc herniation, or lumbosacral damage) and non-specific LBP. Articles encompassing pain caused by, eg, cancer, inflammation, sickness absenteeism, or fractures (proxy measurements) were excluded. We restricted the study design to cohort and case–control studies and excluded cross-sectional studies due to the lack of temporality. The language was restricted to English and the Nordic languages.

### Data extraction and risk of bias

From each included article, we extracted data on author, study design, population, definition and assessment of outcome and exposure, confounders, measures of associations, and 95% CI. If divergent occupational psychosocial exposures occurred in articles measuring the same construct, they were coded according to the domains of Karasek’s job strain model to be fitted in our exposure categorization. One author extracted all relevant data from the included studies, which another author quality-checked. A third author resolved any disagreements in the data extraction.

The methodological quality was critically appraised using a risk of bias tool developed for chronic diseases and used in several previous systematic reviews (supplementary Appendix C) (16–21). The risk of bias tool comprised five major risk domains and three minor risk domains, and the overall risk of bias of each included study was rated as low, moderate, or high. A study was considered to have a low risk of bias if all major domains and at least one minor domain were rated as low risk of bias. For a study to be considered to have a moderate risk of bias, four out of five major domains and at least one minor domain should be rated as low risk of bias. All other combinations were considered as high risk of bias. Two authors independently performed the risk of bias assessment. If the individual assessments differed, the risk of bias assessments was discussed with all authors until a consensus was reached.

### Statistics

Before conducting the meta-analysis, we excluded studies that were based on identical source populations to avoid double-counting data by (i) excluding the study with the highest risk of bias assessment and, if both studies had the same risk of bias assessment, (ii) excluding the study with the fewest participants.

In the meta-analysis, sex-combined estimates were included for measures of association whenever available. In cases where only sex-specific estimates were provided, associations for each sex were selected. The measure of association between the highest and lowest exposure category was included to ensure exposure contrast, employing the most adjusted models. If the adjusted model was prone to over-adjustment due to the inclusion of highly correlated variables, then, if available, we favored the model without such correlations.

A measure of association with risk estimates other than OR was considered approximately equivalent to OR if the incidence proportion of the outcome was <10%, according to Zhang et al (22). Weighted estimates were calculated in the meta-analysis using OR with a corresponding 95% CI using random-effects models (23). Heterogeneity was assessed using I^2^-statistics, describing the percentage of variability, and quantified by the restricted maximum likelihood method (REML) (24). To interpret the I^2^-values, Cochranes thresholds were used (25). We evaluated publication bias by creating Funnel plots and tested the asymmetry using Egger’s test despite a recommendation of ten studies included for a given exposure category. Finally, sensitivity analyses were conducted if at least two studies appeared in each risk of bias category (low-to-moderate versus high), exploring variations and robustness of findings. All analyses were performed using STATA 17.0 (Stata Corp, College Station, TX, USA).

### Level of evidence

The level of evidence across studies was assessed for the association between each occupational psychosocial exposure and chronic LBP. We used the Navigation Guide methodology based upon observational epidemiological studies in environmental and occupational health (26) and encompassing the Grading of Recommendations Assessment, Development, and Evaluation (GRADE) (27). By applying guidelines from the Navigation Guide, the level of evidence concerning an association from observational studies started at “moderate” evidence. Two authors independently assessed the level of evidence, and a third author was consulted when discrepancies occurred between ratings. The level of evidence concerning an association could be rated as “high”, “moderate”, “low”, or “very low” (supplementary Appendix D, Table S5). We downgraded based on the risk of bias, inconsistency, indirectness, imprecision, and publication bias and upgraded based on the magnitude of effect, dose-response, and residual confounding (supplementary Appendix D, Table S6).

## Results

### Study selection and characteristics

**Figure 1:**
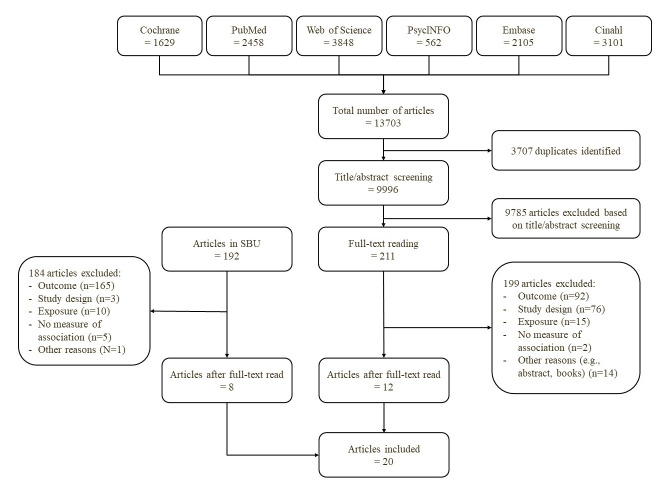
Flowchart.

Figure 1 presents the flow chart, detailing the exclusion process of articles. From the literature search in the SBU, we did a full-text reading of 192 articles which led to the exclusion of 184. In total, 8 articles were considered eligible for inclusion.

The literature searches for articles published after 10 January 2014 initially identified 13 703 articles across all six databases, with 3707 duplicates subsequently removed. Following this, 9996 articles were screened based on title and abstract, leading to the exclusion of 9785 articles. Finally, a full-text reading of 211 articles resulted in a total of 12 articles meeting the eligibility criteria for this systematic review. In total, 20 articles were included in the systematic review. Details regarding the reasons for all exclusions are provided in supplementary Appendix E, table S8 and S9.

Short characteristics of each included study are provided in table 1, and a more comprehensive description is included in Appendix F, table S10 (28–47). Our systematic review included 18 cohort studies and two case–control studies. Chronic LBP was assessed using questionnaires in 11 studies, interviews in three studies, registers in two studies, physical- and radiologic examination in one study, interview and physical examination in one study, radiographic in one study, and diaries in one study. The exposure was assessed using questionnaires in 17 studies and interviews in three studies. The included studies were conducted in Denmark (35, 46), France (30, 33, 47), Germany (36, 43), Sweden (32), The Netherlands (34), USA (31), New Zealand (40–42), Japan (37–39), Iran (28, 44), Thailand (45), and Bangladesh (29), and published between 2002 and 2019.

**Table 1:** Characteristics of studies. [CC=case–control; BJSQ=Brief Job Stress Questionnaire; CUPID=Cultural and Psychosocial Influences on Disability; JCQ=Job Content Questionnaire; MUSIC=Musculoskeletal intervention Center-Norrtälje Study; LBP=low back pain; LDH=lumbar disc herniation; OC=osteochondrosis; S-ERI=short effort-reward imbalance; SL=spondylosis; PE=physical examination; RE=radiological examination; SR=self-reported].

Reference	Country	Design	Follow-up time	Population ^a^	Age (years) Mean (SD)	Exposure assessment	Outcome definition
Aghilinejad 2015 (28)	Iran	Cohort	>3 months	185 metal workers (men)	36 (7.3)	Questionnaire (MUSIC)	SR LBP for >3 months
Ahsan 2013 (29)	Bangladesh	CC	NA	200 cases and 200 controls, mixed workers	39.5	Interview	LDH patients attending a spine unit with LBP
Esquirol 2017 (30)	France	Cohort	5 years	1560 mixed workers	32, 42, or 52 ^b^	Questionnaire (using Karasek’s model)	SR LBP >6 months
Gold 2017 (31)	US	Cohort	6 years	1154 mixed health professionals	41 (13.1)	Questionnaire (JCQ)	SR LBP for 3 months
Halonen 2018 (32)	Sweden	Cohort	6 years	1845 mixed workers	16–64	Questionnaire (S-ERI)	SR LBP for 3 months
Herin 2014 (33)	France	Cohort	5 years	5837 mixed workers	42, 47, 52, or 57 ^b^	Questionnaire (using Karasek’s model)	PE + SR LBP for >6 months + clinical signs
Jansen 2004 (34)	Netherlands	Cohort	1 year	523 mixed workers	41 (9.7)	Questionnaire (using Karasek’s model)	SR LBP with disability in the past 12 months
Jørgensen 2013 (35)	Denmark	Cohort	32–33 years	3833 mixed workers	40-59	Questionnaire	Hospitalisation due to LDH.
Latza 2002 (36)	Germany	Cohort	3 years	488 construction workers (men)	33 (10.0)	Interview	>90 days of SR LBP in the last 12 months
Matsudaira 2014 (37)	Japan	Cohort	1 year	3811 mixed workers	43 (10.1)	Questionnaire (BJSQ)	SR disabling LBP for >3 months
Matsudaira 2015 (38)	Japan	Cohort	1 year	3811 mixed workers	43 (10.1)	Questionnaire (BJSQ)	SR disabling LBP for >3 months
Matsudaira 2019 (39)	Japan	Cohort	1 year	198 mixed workers	36 (9.1)	Questionnaire (CUPID)	SR disabling LBP for >3 months
Melloh 2013 (40)	New Zealand	Cohort	6 months	168 mixed workers	36 (13.1)	Questionnaire (JCQ)	SR persistent LBP for 6 months
Melloh 2013 (41)	New Zealand	Cohort	6 months	169 mixed workers	36 (13.1)	Questionnaire (JCQ)	SR persistent LBP for 3 months
Melloh 2013 (42)	New Zealand	Cohort	>3 months	195 mixed workers	36 (13.1)	Questionnaire (JCQ)	SR persistent LBP for 3 months
Seidler 2003 (43)	Germany	CC	NA	225 cases and 107 controls, mixed workers (men)	44	Interview	LDH + mod./severe OC or SL, in- or outpatients
Seyedmehdi 2016 (44)	Iran	Cohort	1 year	511 workers at rubber factory	38 (5.8)	Questionnaire (JCQ)	SR LBP >3 months
Sihawong 2016 (45)	Thailand	Cohort	1 year	609 office workers	36 (8.3)	Questionnaire (JCQ)	SR LBP >3 months
Sørensen 2011 (46)	Denmark	Cohort	32–33 years	3833 mixed workers	40–59	Questionnaire	Hospitalisation due to LDH.
Tubach 2004 (47)	France	Cohort	2 years	475 mixed workers	35–50	Questionnaire	Sciatica + visiting physician

^a^ Number of participants included in the analysis regarding occupational psychosocial exposures and chronic low back pain. ^b^ Categorical age and not a mean age.

### Risk of bias

The risk of bias of each included studies is presented in table 2. Based on the risk of bias assessments, 1 study was rated as having a low risk of bias, 7 as a moderate risk of bias, and 13 as a high risk of bias. Notably, the ‘enrolment’ domain was the most common source of bias due to the lack of drop-out analyses and the inclusion of participants with the outcome at baseline.

**Table 2:** Risk of bias assessment of all 20 studies. [✔=comply with criteria; **×**=does not comply with criteria; ?=no information was provided. Major domains comprised 1) Study design & selection, 2) Exposure, 3) Outcome, 4) Enrolment, and 5) Analysis method].

Studies	Risk of bias	Domains								
		Major						Minor		
		Study design & selection	Exposure	Outcome	Enrolment	Analysis method ^a^		Funding	Chronology	Conflict of interest
Aghilinejad 2015 (28)	High	✔	✔	✔	×	✔		×	×	**?**
Ahsan 2013 (29)	High	×	**?**	✔	×	×		×	**?**	**?**
Esquirol 2017 (30)	Moderate	×	✔	✔	✔	✔		✔	✔	✔
Gold 2017 (31)	Moderate	×	✔	✔	✔	✔		✔	✔	✔
Halonen 2018 (32)	Moderate	×	✔	✔	✔	✔		✔	✔	✔
Herin 2014 (33)	Moderate	×	✔	✔	✔	✔		✔	✔	✔
Jansen 2004 (34)	Low	✔	✔	✔	✔	✔		×	✔	**?**
Jørgensen 2013 (35)	Moderate	✔	×	✔	✔	✔		×	✔	**?**
Latza 2002 (36)	High	×	×	✔	×	✔		✔	×	×
Matsudaira 2014 (37)	High	×	✔	✔	×	✔		✔	×	✔
Matsudaira 2015 (38)	High	×	✔	✔	×	×		×	×	**?**
Matsudaira 2019 (39)	High	×	✔	✔	×	×		✔	×	**?**
Melloh 2013 (40)	High	×	✔	✔	×	✔		✔	×	**?**
Melloh 2013 (41)	High	×	✔	✔	×	✔		×	×	✔
Melloh 2013 (42)	High	×	✔	✔	×	✔		×	×	**?**
Seidler 2003 (43)	Moderate	✔	✔	✔	×	✔		✔	×	**?**
Seyedmehdi 2016 (44)	High	✔	✔	✔	×	✔		×	×	**?**
Sihawong 2016 (45)	High	×	✔	✔	×	✔		✔	×	✔
Sørensen 2001 (46)	Moderate	✔	×	✔	✔	✔		✔	✔	✔
Tubach 2004 (47)	High	✔	×	✔	×	✔		×	×	✔

^a^ Minor domains comprised 6) Funding, 7) Chronology, and 8) Conflict of interest.

### Meta-analysis

The measures of associations are presented in supplementary Appendix G, table S11. The occupational psychosocial exposures included in the meta-analysis comprised job control, demand, stain, support, stress, and job satisfaction. Occupational psychosocial exposures not fitting the preceding categories were categorized as “others” and were not statistically investigated due to large heterogeneity. Moreover, four studies used different measures of associations than OR (34–36, 46). None of the studies contained an incidence proportion of the outcome as >10% and the effect estimate was treated equally as an OR.

We noted variations in how job support, job control, and job satisfaction were assessed in four studies, differing from the rest (36, 37, 39, 47). Before calculating the pooled OR, we calculated the reciprocal value (effect sizes were reversed) of the effect estimates and the 95% CI to align effect sizes across exposure categories.

### Job control

**Figure 2:**
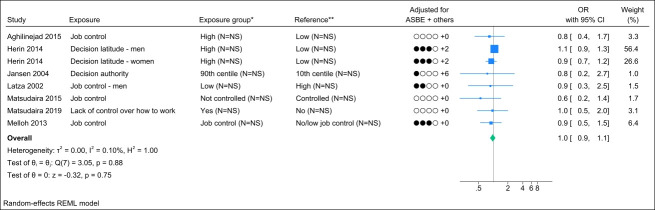
Forest plot of the association between job control and chronic low back pain. [NS=not stated; ASBE=adjusted for age, sex, BMI, and education. ‘+ others’ refer to adjustments of other confounding variables. * Number of exposed participants with chronic low back pain and number of exposed references. **Number of non-exposed participants with chronic low back pain and non-exposed references.

Seven studies assessed job control, and no identical study populations were identified. Figure 2 presents the forest plot, showing a pooled OR of 1.0 (95% CI 0.9–1.1) with a non-important heterogeneity (I^2^ value of 0.1%). The funnel plot could indicate publication bias (supplementary Appendix H, figure S1), but Egger’s test did not yield a significant P-value (0.20). Grading the level of evidence, the confidence of evidence concerning an association is very low (Appendix D, Table S7).

### Job demand

**Figure 3:**
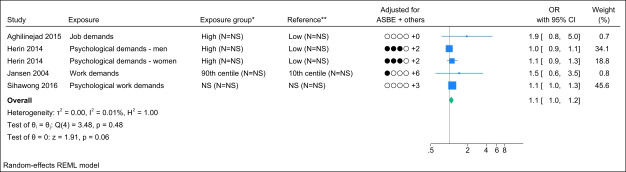
Forest plot of the association between job demand and chronic low back pain. NS=not stated; ASBE=adjusted for age, sex, BMI, and education. ‘+ others’ refer to adjustments of other confounding variables. * Number of exposed participants with chronic low back pain and number of exposed references. **Number of non-exposed participants with chronic low back pain and non-exposed references.

Four studies assessed job demand, and no identical study populations were identified. Figure 3 presents the forest plot, showing a pooled OR of 1.1 (95% CI 1.0–1.2) with a non-important heterogeneity (I^2^ value of 0.01%). Interpreting the funnel plot was challenging due to the limited number of studies (supplementary Appendix H, figure S2). However, Egger’s test yielded a non-significant P-value (0.20). Grading the level of evidence, the confidence of evidence concerning an association is very low (supplementary Appendix D, table S7).

### Job strain

**Figure 4:**
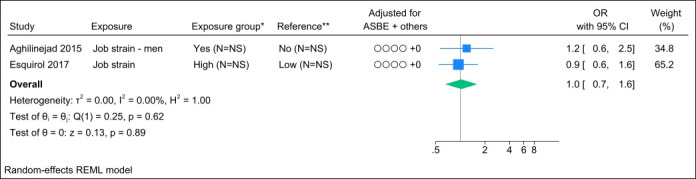
Forest plot of the association between job strain and chronic low back pain. NS=not stated; ASBE=adjusted for age, sex, BMI, and education. ‘+ others’ refer to adjustments of other confounding variables. *Number of exposed participants with chronic low back pain and number of exposed references. **Number of non-exposed participants with chronic low back pain and non-exposed references.

Two studies assessed job strain, and no identical study populations were identified. Figure 4 presents the forest plot and shows a pooled OR of 1.0 (95% CI 0.7–1.6). Statistical assessment of heterogeneity and publication bias was hindered by the limited number of studies. Grading the level of evidence, the confidence of evidence concerning an association is very low. The 95% CI is compatible with both a beneficial and harmful effect (supplementary Appendix D, table S7).

### Job support

**Figure 5:**
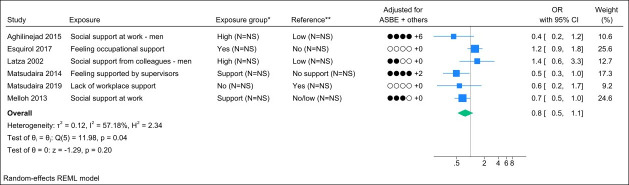
Forest plot of the association between job support and chronic low back pain. NS=not stated; ASBE=adjusted for age, sex, BMI, and education. ‘+ others’ refer to adjustments of other confounding variables. *Number of exposed participants with chronic low back pain and number of exposed references. **Number of non-exposed participants with chronic low back pain and non-exposed references.

Nine studies assessed job support, and three studies were excluded due to identical study populations (38, 41, 42). Figure 5 presents the forest plot and shows a pooled OR of 0.8 (95% CI 0.5–1.1) with a moderate degree of heterogeneity (I^2^ value of 57%). The funnel plot did not indicate publication bias (supplementary Appendix H, figure S3), and Egger’s test yielded a non-significant P-value (0.43). Grading the level of evidence, the confidence of evidence concerning an association is very low. The 95% CI is compatible with a beneficial effect (supplementary Appendix D, table S7).

### Job stress

**Figure 6:**
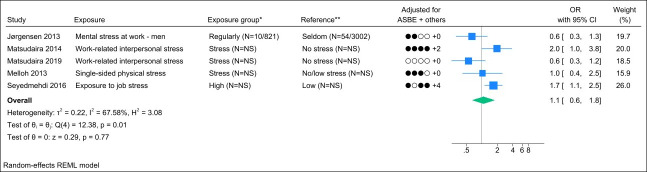
Forest plot of the association between job stress and chronic low back pain. NS=not stated; ASBE=adjusted for age, sex, BMI, and education. ‘+ others’ refer to adjustments of other confounding variables. *Number of exposed participants with chronic low back pain and number of exposed references. **Number of non-exposed participants with chronic low back pain and non-exposed references.

Seven studies assessed job stress, and two studies were excluded due to identical study populations (38, 46). Figure 6 presents the forest plot and shows a pooled OR of 1.1 (95% CI 0.6–1.8) with a substantial degree of heterogeneity (I^2^ value of 68%). The funnel plot could indicate publication bias (Appendix H, figure S4). However, Egger’s test yielded a non-significant p-value (0.29). Grading the level of evidence, the confidence of evidence concerning an association is very low. The 95% CI is compatible with both a beneficial and harmful effect (supplementary Appendix D, table S7).

### Job satisfaction

**Figure 7:**
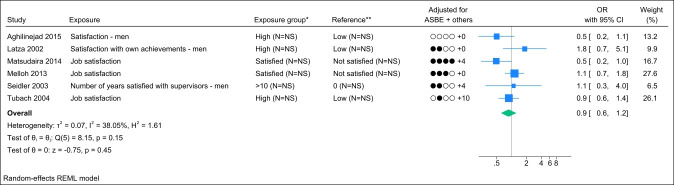
Forest plot of the association between job satisfaction and chronic low back pain. NS=not stated; ASBE= adjusted for age, sex, BMI, and education. ‘+ others’ refer to adjustments of other confounding variables. *Number of exposed participants with chronic low back pain and number of exposed references. **Number of non-exposed participants with chronic low back pain and non-exposed references.

Six studies assessed job satisfaction, and no identical study populations were identified. Figure 7 presents the forest plot, showing a pooled OR of 0.9 (95% CI 0.6–1.2) with a non-important to moderate degree of heterogeneity (I^2^ value of 38%). The funnel plot did not indicate publication bias (supplementary Appendix H, figure S5), and Egger’s test yielded a non-significant P-value (0.90). Grading the level of evidence, the confidence of evidence concerning an association is very low. The 95% CI is compatible with both a beneficial and harmful effect (supplementary Appendix D, table S7).

### Sensitivity analysis

Only two occupational psychosocial exposure categories had sufficient number of studies in each risk of bias category: job control and job demand. Concerning job control, studies with low to moderate risk of bias studies showed an OR of 1.0 (95% CI 0.9–1.2), while high risk of bias studies showed an OR of 0.9 (95% CI 0.6–1.2). For job demand, studies with low/moderate risk of bias studies compared to those with a high risk of bias demonstrated OR of 1.0 (95% CI 0.9–1.2) and 1.2 (95% CI 0.8–1.7), respectively.

## Discussion

### Summary of findings

In this systematic review and meta-analysis, we investigated the association between occupational psychosocial exposures (ie, job control, demand, strain, support, stress, and satisfaction) and chronic LBP defined as persistent pain ≥3 months. In total, 20 case-control and cohort studies were included. For the six occupational psychosocial exposures, pooled OR ranged from 0.8 to 1.1. In addition, the sensitivity analyses showed no notable differences between high risk and low/moderate risk of bias studies. Based on this systematic review, we found no association between occupational psychosocial exposures and chronic LBP. When grading the level of evidence of an association, very low level of evidence was found for all occupational psychosocial exposures.

### Methodological considerations

The strengths of the systematic review were the literature search conducted in several databases, and the exclusion of studies, data extraction, and risk of bias assessment conducted independently by two authors. A further strength was the general transparency of each step in our systematic review including the excluded studies, data extraction, risk of bias assessment, measures of association used in the meta-analysis, funnel plots, and level of evidence presented in the Appendices. Furthermore, by excluding cross-sectional studies, we aimed to evaluate causality, emphasizing the importance of the temporal relation between exposure and outcome. Finally, the level of evidence for the association between the occupational psychosocial exposures and chronic LBP was assessed using GRADE.

The study also has some limitations. Even though a systematic literature search was conducted, we might not have included all relevant studies, for instance, due to language restrictions. Also, only one out of 20 studies was assessed as having a low risk of bias. The meta-analyses and forest plots were conducted despite the low number of studies for each occupational psychosocial exposure and with heterogeneity between studies. Due to the few studies, we only conducted sensitivity analysis based on the risk of bias assessment. Stratified meta-analysis of low/moderate vs. high risk of bias studies showed similar results but leaves the analysis with limited power due to few studies in each group increasing the risk of type 2 error.

In line with the scientific literature on chronic pain, we defined chronic LBP as pain lasting for >3 months, ensuring consistency and reduced outcome variance. Therefore, studies lacking a clear definition of pain duration were excluded. We observed that multiple studies combined groups with varying pain durations into one group (pain in 30–90 days and >90 days) (48–50). These studies were excluded since it was not possible to distinguish the effect of occupational psychosocial exposures on chronic LBP from sub-acute LBP. Additionally, studies lacking a precise specification of pain location, such as those using the term ‘back problems’, were also excluded. Complying with these criteria has reduced the number of studies eligible for inclusion, possibly affecting the findings of our review compared to the existing literature on LBP. However, the criteria strengthen the accuracy of the outcome and the comparability between studies.

Quantifying pain is a complex task, as the trajectory patterns of pain often show considerable variability. This suggests that an episode of LBP may not only be a temporary occurrence but a part of a broader, ongoing chronic condition, challenging the categorization of chronic LBP cases. The time-dependent nature of pain makes it difficult to distinguish between temporary episodes of pain (acute LBP) and a more persistent, chronic condition (chronic LBP). The complexity of pain quantification and variability might lead to misclassification of the outcome, affecting the association between occupational psychosocial exposures and chronic LBP.

The assessment of occupational psychosocial exposures remained consistent across the included studies. Seventeen studies utilized questionnaires, while three employed interviews; therefore, all measurements relied on self-reports. Despite the utilization of self-report, objective measurements of occupational psychosocial exposures can be challenging to obtain and may be just as prone to inaccuracies as self-reported measurements.

Addressing this complexity of behavior and resilience is essential but imposes high methodological demands (51). Moreover, most studies utilized questionnaires considered reliable and valid to measure psychosocial exposures (Job Content Questionnaire or Brief Job Stress Questionnaire). This strengthens the accuracy and the comparability of the study findings. In contrast, the inclusion of confounding variables varied considerably across the included studies, with four studies presenting unadjusted estimates (28, 30, 38, 39). Conversely, no substantial differences were observed between the unadjusted and adjusted measures of associations.

The time frame from experiencing occupational psychosocial exposures to the measurement point is an important consideration. This latency can introduce bias if participants are prone to over- or underestimate the exposure, either due to difficulties in remembering accurately or, for instance, as a result of job rotation. Moreover, different stages of occupational psychosocial exposures can be experienced and are expected to fluctuate over time due to various job-related factors. In this context, we only identified one study assessing exposure-response relations, investigating different levels of experiences (43). This warrants the need for further investigation into the long-term effects of occupational psychosocial exposures, refinements of measures, and exploration of potential exposure-response relations.

### Comparison with the literature

Overall, we found no association between the six occupational psychosocial exposures and chronic LBP with pooled OR between 0.8 and 1.1. Our results do not support the small but significant association found in the newest systematic reviews for LBP (6, 13) and chronic LBP (14). A major distinction between the systematic reviews concerning chronic LBP comprise the inclusion of cross-sectional studies and the requirements of pain duration. Buruck et al (14) included eight cross-sectional studies, which accounted for 44% of all included studies. Generally, it seemed that the individual cross-sectional studies were in favor of an association and were more likely to find significant results compared to cohort and case-control studies. However, as indicated in their sensitivity analysis, prospective studies also yielded significant associations. The exclusion of cross-sectional studies in our systematic review could have reduced the measure of association compared to the review of Buruck et al. Despite similar outcome criteria in the two systematic reviews of chronic LBP, major differences exist when comparing the included studies in relation to pain duration. Buruck et al included studies combining different pain durations (30–90 days and >90 days) (48–50, 52, 53). These studies were excluded in our systematic review and strictly adhering to the pain duration criteria (>3 months of pain), the significant association found in the review of Buruck et al disappeared. Finally, discrepancies between the inclusion of relevant articles and/or the literature search could have caused differences in the respective findings.

### Implications and conclusion

When examining the association between occupational psychosocial exposures and chronic LBP based on case–control and cohort studies, we found no association. However, the level of evidence was very low, and therefore further high quality studies are warranted. Even though no association was found, occupational psychosocial exposures could still act as potential mediators (part of the causal pathway) or effect modifiers (potentially influencing an association between mechanical exposures and chronic LBP) rather than serving as independent risk factors. Also, occupational psychosocial exposures might have a prognostic value in increasing the duration of pain.

The understanding of any effect of occupational psychosocial exposures that contribute to the development of chronic LBP is crucial for researchers and practitioners in designing interventions or strategies to address occupational health issues effectively. By understanding and integrating this in a clinical and scientific setting, we can gain a broader perspective on the long-term impact and interplay.

## Supplementary material

Supplementary material

## Data Availability

All data are available from the corresponding author upon reasonable request.

## References

[1] Hoy D, Brooks P, Blyth F, Buchbinder R. The Epidemiology of low back pain. Best Pract Res Clin Rheumatol 2010 Dec;24(6):769–81. 10.1016/j.berh.2010.10.002 21665125

[2] Jahn A, Andersen JH, Christiansen DH, Seidler A, Dalbøge A. Occupational mechanical exposures as risk factor for chronic low-back pain: a systematic review and meta-analysis. Scand J Work Environ Health 2023 Oct;49(7):453–65. 10.5271/sjweh.4114 37581384 PMC10838064

[3] Coenen P, Gouttebarge V, van der Burght AS, van Dieën JH, Frings-Dresen MH, van der Beek AJ et al. The effect of lifting during work on low back pain: a health impact assessment based on a meta-analysis. Occup Environ Med 2014 Dec;71(12):871–7. 10.1136/oemed-2014-102346 25165395

[4] Kuijer PP, Verbeek JH, Seidler A, Ellegast R, Hulshof CT, Frings-Dresen MH et al. Work-relatedness of lumbosacral radiculopathy syndrome: review and dose-response meta-analysis. Neurology 2018 Sep;91(12):558–64. 10.1212/01.wnl.0000544322.26939.09 30120136 PMC6161552

[5] Burström L, Nilsson T, Wahlström J. Whole-body vibration and the risk of low back pain and sciatica: a systematic review and meta-analysis. Int Arch Occup Environ Health 2015 May;88(4):403–18. 10.1007/s00420-014-0971-4 25142739

[6] Niedhammer I, Bertrais S, Witt K. Psychosocial work exposures and health outcomes: a meta-review of 72 literature reviews with meta-analysis. Scand J Work Environ Health 2021 Oct;47(7):489–508. 10.5271/sjweh.3968 34042163 PMC8504166

[7] Karasek RA Jr. Job demands, job decision latitude, and mental strain: implications for job redesign. Adm Sci Q 1979;24(2):285–308. 10.2307/2392498

[8] Bongers PM, de Winter CR, Kompier MA, Hildebrandt VH. Psychosocial factors at work and musculoskeletal disease. Scand J Work Environ Health 1993 Oct;19(5):297–312. 10.5271/sjweh.1470 8296178

[9] Hauke A, Flintrop J, Brun E, Rugulies R. The impact of work-related psychosocial stressors on the onset of musculoskeletal disorders in specific body regions: A review and meta-analysis of 54 longitudinal studies. Work Stress 2011;25(3):243–56. 10.1080/02678373.2011.614069

[10] Theorell T, Hasselhorn HM. Music Norrtälje Study Group n. Endocrinological and immunological variables sensitive to psychosocial factors of possible relevance to work-related musculoskeletal disorders. Work Stress 2002;16(2):154–65. 10.1080/02678370210150837

[11] Bongers PM, Ijmker S, van den Heuvel S, Blatter BM. Epidemiology of work related neck and upper limb problems: psychosocial and personal risk factors (part I) and effective interventions from a bio behavioural perspective (part II). J Occup Rehabil 2006 Sep;16(3):279–302. 10.1007/s10926-006-9044-1 16850279

[12] Hartvigsen J, Lings S, Leboeuf-Yde C, Bakketeig L. Psychosocial factors at work in relation to low back pain and consequences of low back pain; a systematic, critical review of prospective cohort studies. Occup Environ Med 2004 Jan;61(1):e2. 14691283 PMC1757801

[13] SBU. Arbetsmiljöns betydelse för ryggproblem. En systematisk litteraturöversikt. [Occupational Exposures and Back Disorders. Swedish Council on Health Technology Assessment 2014.] Stockholm: Statens beredning för medicinsk utvärdering (SBU). 2014. ISBN 978-91-85413-68-3;SBU-rapport nr 227.

[14] Buruck G, Tomaschek A, Wendsche J, Ochsmann E, Dörfel D. Psychosocial areas of worklife and chronic low back pain: a systematic review and meta-analysis. BMC Musculoskelet Disord 2019 Oct;20(1):480. 10.1186/s12891-019-2826-3 31653249 PMC6814972

[15] Merskey H, Bogduk N. Classification of chronic pain: descriptions of chronic pain syndromes and definitions of pain terms. 2nd ed: Seattle: International Association for the Study of Pain; 1994.

[16] Shamliyan TA, Kane RL, Ansari MT, Raman G, Berkman ND, Grant M et al. Development quality criteria to evaluate nontherapeutic studies of incidence, prevalence, or risk factors of chronic diseases: pilot study of new checklists. J Clin Epidemiol 2011 Jun;64(6):637–57. 10.1016/j.jclinepi.2010.08.006 21071174

[17] Bolm-Audorff U, Hegewald J, Pretzsch A, Freiberg A, Nienhaus A, Seidler A. Occupational Noise and Hypertension Risk: A Systematic Review and Meta-Analysis. Int J Environ Res Public Health 2020 Aug;17(17):6281. 10.3390/ijerph17176281 32872306 PMC7504405

[18] Romero Starke K, Kofahl M, Freiberg A, Schubert M, Groß ML, Schmauder S et al. The risk of cytomegalovirus infection in daycare workers: a systematic review and meta-analysis. Int Arch Occup Environ Health 2020 Jan;93(1):11–28. 10.1007/s00420-019-01464-x 31359142

[19] Ijaz S, Verbeek J, Seidler A, Lindbohm ML, Ojajärvi A, Orsini N et al. Night-shift work and breast cancer--a systematic review and meta-analysis. Scand J Work Environ Health 2013 Sep;39(5):431–47. 10.5271/sjweh.3371 23804277

[20] Jahn A, Andersen JH, Christiansen DH, Seidler A, Dalbøge A. Occupational mechanical exposures as risk factor for chronic low-back pain: a systematic review and meta-analysis. Scand J Work Environ Health 2023 Oct;49(7):453–65. 10.5271/sjweh.4114 37581384 PMC10838064

[21] Jahn A, Nielsen ML, Kyndi M, Dalbøge A. Correction: Association between night work and prostate cancer: a systematic review and meta-analysis. Int Arch Occup Environ Health 2024 Mar;97(2):217. 10.1007/s00420-024-02051-5 38315196

[22] Zhang J, Yu KF. What’s the relative risk? A method of correcting the odds ratio in cohort studies of common outcomes. JAMA 1998 Nov;280(19):1690–1. 10.1001/jama.280.19.1690 9832001

[23] Borenstein M, Hedges LV, Higgins JP, Rothstein HR. Introduction to Meta-Analysis: John Wiley & Sons, Ltd; 2009.

[24] Langan D, Higgins JP, Jackson D, Bowden J, Veroniki AA, Kontopantelis E et al. A comparison of heterogeneity variance estimators in simulated random-effects meta-analyses. Res Synth Methods 2019 Mar;10(1):83–98. 10.1002/jrsm.1316 30067315

[25] Deeks JJ. Altman DG (editors). Chapter 10: Analysing data and undertaking meta-analyses. In: Higgins JPT, Thomas J, Chandler J, Cumpston M, Li T, Page MJ, Welch VA (editors). Cochrane, 2022. Updated February 2022;Cochrane Handbook for Systematic Reviews of Interventions version 6.3. Available from: www.training.cochrane.org/handbook

[26] Woodruff TJ, Sutton P. The Navigation Guide systematic review methodology: a rigorous and transparent method for translating environmental health science into better health outcomes. Environ Health Perspect 2014 Oct;122(10):1007–14. 10.1289/ehp.1307175 24968373 PMC4181919

[27] Guyatt G, Oxman AD, Akl EA, Kunz R, Vist G, Brozek J et al. GRADE guidelines: 1. Introduction-GRADE evidence profiles and summary of findings tables. J Clin Epidemiol 2011 Apr;64(4):383–94. 10.1016/j.jclinepi.2010.04.026 21195583

[28] Aghilinejad M, Tavakolifard N, Mortazavi SA, Kabir Mokamelkhah E, Sotudehmanesh A, Mortazavi SA. The effect of physical and psychosocial occupational factors on the chronicity of low back pain in the workers of Iranian metal industry: a cohort study. Med J Islam Repub Iran 2015 Jul;29:242. 26793633 PMC4715412

[29] Ahsan MK, Matin T, Ali MI, Ali MY, Awwal MA, Sakeb N. Relationship between physical work load and lumbar disc herniation. Mymensingh Med J 2013 Jul;22(3):533–40. 23982545

[30] Esquirol Y, Niezborala M, Visentin M, Leguevel A, Gonzalez I, Marquié JC. Contribution of occupational factors to the incidence and persistence of chronic low back pain among workers: results from the longitudinal VISAT study. Occup Environ Med 2017 Mar;74(4):243–51. 10.1136/oemed-2015-103443 27672181

[31] Gold JE, Punnett L, Gore RJ; ProCare Research Team. Predictors of low back pain in nursing home workers after implementation of a safe resident handling programme. Occup Environ Med 2017 Jun;74(6):389–95. 10.1136/oemed-2016-103930 27919063 PMC5860804

[32] Halonen JI, Virtanen M, Leineweber C, Rod NH, Westerlund H, Magnusson Hanson LL. Associations between onset of effort-reward imbalance at work and onset of musculoskeletal pain: analyzing observational longitudinal data as pseudo-trials. Pain 2018 Aug;159(8):1477–83. 10.1097/j.pain.0000000000001230 29596159

[33] Herin F, Vézina M, Thaon I, Soulat JM, Paris C; ESTEV group. Predictive risk factors for chronic regional and multisite musculoskeletal pain: a 5-year prospective study in a working population. Pain 2014 May;155(5):937–43. 10.1016/j.pain.2014.01.033 24561229

[34] Jansen JP, Morgenstern H, Burdorf A. Dose-response relations between occupational exposures to physical and psychosocial factors and the risk of low back pain. Occup Environ Med 2004 Dec;61(12):972–9. 10.1136/oem.2003.012245 15550602 PMC1740687

[35] Jørgensen MB, Holtermann A, Gyntelberg F, Suadicani P. Physical fitness as a predictor of herniated lumbar disc disease - a 33-year follow-up in the Copenhagen male study. BMC Musculoskelet Disord 2013 Mar;14:86. 10.1186/1471-2474-14-86 23497269 PMC3599998

[36] Latza U, Pfahlberg A, Gefeller O. Impact of repetitive manual materials handling and psychosocial work factors on the future prevalence of chronic low-back pain among construction workers. Scand J Work Environ Health 2002 Oct;28(5):314–23. 10.5271/sjweh.680 12432984

[37] Matsudaira K, Konishi H, Miyoshi K, Isomura T, Inuzuka K. Potential risk factors of persistent low back pain developing from mild low back pain in urban Japanese workers. PLoS One 2014 Apr;9(4):e93924. 10.1371/journal.pone.0093924 24714616 PMC3979726

[38] Matsudaira K, Kawaguchi M, Isomura T, Inuzuka K, Koga T, Miyoshi K et al. Assessment of psychosocial risk factors for the development of non-specific chronic disabling low back pain in Japanese workers-findings from the Japan Epidemiological Research of Occupation-related Back Pain (JOB) study. Ind Health 2015;53(4):368–77. 10.2486/indhealth.2014-0260 26051289 PMC4551067

[39] Matsudaira K, Takahashi M, Kawaguchi M, Hamaguchi A, Haga Y, Koga T. Assessment of risk factors for non-specific chronic disabling low back pain in Japanese workers-findings from the CUPID (Cultural and Psychosocial Influences on Disability) study. Ind Health 2019 Aug;57(4):503–10. 10.2486/indhealth.2018-0157 30344231 PMC6685796

[40] Melloh M, Elfering A, Stanton TR, Käser A, Salathé CR, Barz T et al. Who is likely to develop persistent low back pain? A longitudinal analysis of prognostic occupational factors. Work 2013 Jan;46(3):297–311. 10.3233/WOR-131672 24004738

[41] Melloh M, Elfering A, Chapple CM, Käser A, Rolli Salathé C, Barz T et al. Prognostic occupational factors for persistent low back pain in primary care. Int Arch Occup Environ Health 2013 Apr;86(3):261–9. 10.1007/s00420-012-0761-9 22434236

[42] Melloh M, Salathé CR, Elfering A, Käser A, Barz T, Aghayev E et al. Occupational, personal and psychosocial resources for preventing persistent low back pain. Int J Occup Saf Ergon 2013;19(1):29–40. 10.1080/10803548.2013.11076964 23498709

[43] Seidler A, Bolm-Audorff U, Siol T, Henkel N, Fuchs C, Schug H et al. Occupational risk factors for symptomatic lumbar disc herniation; a case-control study. Occup Environ Med 2003 Nov;60(11):821–30. 10.1136/oem.60.11.821 14573712 PMC1740425

[44] Seyedmehdi SM, Dehghan F, Ghaffari M, Attarchi M, Khansari B, Heidari B et al. Effect of General Health Status on Chronicity of Low Back Pain in Industrial Workers. Acta Med Iran 2016 Mar;54(3):211–7. 27107527

[45] Sihawong R, Sitthipornvorakul E, Paksaichol A, Janwantanakul P. Predictors for chronic neck and low back pain in office workers: a 1-year prospective cohort study. J Occup Health 2016;58(1):16–24. 10.1539/joh.15-0168-OA 26498979

[46] Sørensen IG, Jacobsen P, Gyntelberg F, Suadicani P. Occupational and other predictors of herniated lumbar disc disease-a 33-year follow-up in the Copenhagen male study. Spine 2011 Sep;36(19):1541–6. 10.1097/BRS.0b013e3181f9b8d4 21270695

[47] Tubach F, Beauté J, Leclerc A. Natural history and prognostic indicators of sciatica. J Clin Epidemiol 2004 Feb;57(2):174–9. 10.1016/S0895-4356(03)00257-9 15125627

[48] van den Heuvel SG, Ariëns GA, Boshuizen HC, Hoogendoorn WE, Bongers PM. Prognostic factors related to recurrent low-back pain and sickness absence. Scand J Work Environ Health 2004 Dec;30(6):459–67. 10.5271/sjweh.835 15633597

[49] Hoogendoorn WE, Bongers PM, de Vet HC, Houtman IL, Ariëns GA, van Mechelen W et al. Psychosocial work characteristics and psychological strain in relation to low-back pain. Scand J Work Environ Health 2001 Aug;27(4):258–67. 10.5271/sjweh.613 11560340

[50] Hooftman WE, van der Beek AJ, Bongers PM, van Mechelen W. Is there a gender difference in the effect of work-related physical and psychosocial risk factors on musculoskeletal symptoms and related sickness absence? Scand J Work Environ Health 2009 Mar;35(2):85–95. 10.5271/sjweh.1316 19337673

[51] Crosswell AD, Lockwood KG. Best practices for stress measurement: how to measure psychological stress in health research. Health Psychol Open 2020 Jul;7(2):2055102920933072. 10.1177/2055102920933072 32704379 PMC7359652

[52] Brage S, Sandanger I, Nygård JF. Emotional distress as a predictor for low back disability: a prospective 12-year population-based study. Spine 2007 Jan;32(2):269–74. 10.1097/01.brs.0000251883.20205.26 17224825

[53] Eriksen W, Bruusgaard D, Knardahl S. Work factors as predictors of intense or disabling low back pain; a prospective study of nurses’ aides. Occup Environ Med 2004 May;61(5):398–404. 10.1136/oem.2003.008482 15090659 PMC1740782

