# Supplementary material for: Occupational psychosocial exposures and chronic low-back pain: a systematic review and meta-analysis
Source: Scand J Work Environ Health. 2024 Jun 27;50(5):329–40. doi: 10.5271/sjweh.4165 (PMC11214847; doi:10.5271/sjweh.4165)

# Occupational psychosocial exposures and chronic low back pain: a systematic review and meta-analysis

Alexander Jahn[1], Johan H. Andersen[2,3], Andreas Seidler[4], David H. Christiansen[2,5,6], Annett Dalbøge[1,2].

[1] Danish Ramazzini Centre, Department of Occupational Medicine, Aarhus University Hospital, Aarhus, Denmark.

[2] Department of Clinical Medicine, Aarhus University, Aarhus, Denmark.

[3] Department of Occupational Medicine - University Research Clinic, Danish Ramazzini Centre, Goedstrup Hospital, Herning, Denmark.

[4] Institute and Policlinic of Occupational and Social Medicine (IPAS), Faculty of Medicine, Technische Universität Dresden, Fetscherstr, 74, 01307 Dresden, Germany.

[5] Elective Surgery Centre, Silkeborg Regional Hospital, Silkeborg, Denmark.

[6] Centre of Health and Nursing Research, Regional Hospital Central Jutland, Viborg, Denmark.

**Corresponding author**

Alexander Jahn, Danish Ramazzini Centre, Department of Occupational Medicine, Palle Juul-Jensens Boulevard 99, Aarhus University Hospital, Aarhus N, Denmark.

E-mail: alexjn@rm.dk, phone number: +0045 78450900.

# Content

**Appendix A**. Literature search ... 3
**Table 1**. Literature search ... 3
**Appendix B**. PECOS ... 8
**Table 2**. Inclusion and exclusion criteria for the systematic review. ... 8
**Appendix C**. Risk of bias assessment tool ... 9
**Table 3**. Risk of bias assessment tool, case-control studies. ... 9
**Table 4**. Risk of bias assessment tool, cohort studies. ... 10
**Appendix D**. Grading the evidence of an association ... 11
**Table 5.** Quality level - four levels of evidence. ... 11
**Table 6.** Evaluating the quality of evidence - down- and upgrading factors. ... 11
**Table 7**. Results of GRADE. ... 13
**Appendix E**. Excluded articles. ... 14
**Table 8**. Excluded articles based on full-text read and reason for exclusion. ... 14
**Table 9.** Excluded articles based on full-text read and reason for exclusion. ... 21
**Appendix F**. Study characteristics ... 30
**Table 10**. Description of each included study ... 30
**Appendix G**. Measure of associations ... 35
**Table 11**. Measures of associations of each included study ... 35
**Appendix H**. Funnel plots ... 44
**Figure 1**. Job control ... 44
**Figure 2.** Job demand ... 44
**Figure 3**. Job support ... 45
**Figure 4**. Job stress ... 45
**Figure 5**. Job satisfaction ... 46

## **Appendix A**. Literature search

Our literature search consisted of a block search containing four blocks combining them with the Boolean operator "AND" between blocks and "OR" within blocks.

**Table S1**. Literature search

| **Block 1.** Work. | **Free text search in title and abstract** |
|---|---|
| **Mesh** | |
| "Occupations"[Mesh] | occupation*, employ*, job*, working condition*, work-related work-load*, work-place*, work environment* |
| "Occupational Health"[Mesh] | |
| "Occupational Diseases"[Mesh] | |
| "Occupational Exposure"[Mesh] | |
| "Occupational Groups"[Mesh] | |
| "Work"[Mesh] | |
| "Workplace"[Mesh] | |
| "Workload"[Mesh] | |
| "Women, Working"[Mesh] | |
| "Employment"[Mesh] | |

| **Block 2**. Exposure. | **Free text search in title and abstract** |
|---|---|
| **Mesh** | |
| **Mechanical stress** | |
| "Stress, Mechanical"[Mesh] | |
| **Organisational and psychosocial factors** | |
| "Stress, Psychological"[Mesh] | |
| **Development of force** | lift*, carry*, hold*, pull*, drag*, push*, manual handling, force*, biomechanic*, physical demand*, physically demand*. |
| "Lifting"[Mesh] | |
| "Weight-Bearing"[Mesh] | |
| "Biomechanics"[Mesh] | |
| "Moving and Lifting Patients"[Mesh] | |
| "Physical Exertion"[Mesh] | |
| **Working postures** | flexion*, extension*, turning*, sitting*, kneeling*, twisting*, bending, sedentary, walking*, reaching, squatting, standing, postural balance, static AND posture, awkward AND posture. |
| "Torsion, Mechanical"[Mesh] | |
| "Postural Balance"[Mesh] | |
| "Walking"[Mesh] | |
| **Working movement** | repetitive movement*, monotonous work, dynamic AND posture, relaxation, recovery of function, static work, dynamic load. |
| "Recovery of Function"[Mesh] | |

| | |
|---|---|
| ”Relaxation”[Mesh] | |
| **Influence and demand** | decision latitude, work demand*, job demand*, high demand*, low control, work control, job control, work influence*, demand resource*, lack of control, job strain, work strain. |
| **Effort and reward** | effort reward*, time pressure*, work overload*, recuperation*, recovery. |
| **Social support and relations in the workplace** | support system*, social network*, emotional support, justice*, injustice*, interaction*, interpersonal relation*. |
| ”Social Support”[Mesh] | |
| ”Employee Performance "Appraisal”[Mesh] | |
| ”Organizational Culture”[Mesh] | |
| "Justice/psychology”[Mesh] | |
| ”Communication/psychology”[Mesh] | |
| ”Interpersonal Relations”[Mesh] | |
| **Job satisfaction** | Boredom, job satisfaction, work satisfaction, coping, work ability. |
| ”Job Satisfaction”[Mesh] | |
| ”Employee Grievances”[Mesh] | |
| **Education and learning:** | skill discretion*, staff development. |
| ”Staff Development”[Mesh] | |
| **Conflict, violence or harassment** | harass*, workplace conflict*, workplace violen*, silent workplace*, victimization*, bullying, role ambiguity, role conflict*, work role*, discrimination. |
| ”Bullying”[Mesh] | |
| ”Prejudice”[Mesh] | |
| ”Social Discrimination”[Mesh] | |
| **Working time** | working hour*, working time, shift work*, work shift*, day-time, night-time, temporary work, full-time, part-time, flexible work*, lean production. |
| ”Work Schedule Tolerance”[Mesh] | |
| **Job insecurity** | organizational change, job security, job insecurity. |
| ”Personnel Downsizing”[Mesh] | |
| **Chemical and biological substances** | pollut*, indoor air*, airborne, passive smok*, solvent*, smok* AND pollute*. |
| ”Air Pollution”[Mesh] | |
| ”Air Pollutants”[Mesh] | |
| ”Solvents”[Mesh] | |
| ”Fluids and Secretions”[Mesh] | |
| **Contact with chemicals** | hazardous chemical*, hazardous material*, hazardous substance*, toxic action*, pesticide*, poison*. |
| ”Toxic Actions”[Mesh] | |
| **Noise** | noise* |

| | |
|---|---|
| ”Noise”[Mesh] | |
| **Vibrations** | vibrat*, driving, vehicle*, truck*, lorry/lorries, automobile*, car/cars, buses, hand tool*, hand-held tool*, power tool*. |
| ”Vibration”[Mesh] | |
| ”Automobile Driving”[Mesh] | |
| ”Motor Vehicles”[Mesh] | |
| **Radiation** | radiation* |
| ”Radiation”[Mesh] | |
| ”Air Pollution, Radioactive”[Mesh] | |
| **Temperature** | climate*, cold temperature*, hot temperature*. |
| ”Hot Temperature”[Mesh] | |
| ”Cold Temperature”[Mesh] | |
| ”Climate”[Mesh] | |
| **Infected materials** | contagious* communicable disease* |
| ”Communicable Diseases”[Mesh] | |

| **Block 3.** Back problems | **Free text search in title and abstract** |
|---|---|
| **Mesh** | |
| **Back problems** | |
| "Back" [Mesh] | back, spine*, spinal*, trunk*, lumbar*, pelvis*, sacrum, lumbo-sacral*, lumbosacral*, intervertebral disk*, intervertebral disc*, thoracic vertebrae, thoracic vertebra. |
| "Spine” [Mesh] | |
| "Pelvis" [Mesh] | |
| "Pain" [Mesh] | pain, ache*, musculoskeletal disease*, musculoskeletal disorder*, cumulative trauma disorder*, nerve entrapment. |
| "Pain Measurement" [Mesh] | |
| "Cumulative Trauma Disorders" [Mesh] | |
| "Musculoskeletal Diseases" [Mesh] | |
| "Back Pain" [Mesh] | back pain, backache*, back injur*, spinal disease*, spine disease*, spinal injur* OR spine injur*, intervertebral disk degeneration, intervertebral disc degeneration, spinal osteochondros*, spine osteochondros*, Scheuermann*, spinal stenos*, spondylitis, spondylarthritis, spondylosis, lumbago, sciatica, pelvic pain. |
| "Back Injuries" [Mesh] | |
| "Spinal Diseases" [Mesh] | |
| "Pelvic Pain" [Mesh] | |
| "Sciatica" [Mesh] | |

| **Block 4** Language restrictions and publication date. | |
|---|---|
| **Language restrictions** | |

| | |
|---|---|
| English, Danish, Norwegian, or Swedish. | |
| **Publication date** | |
| From 2014/01/01 to 2021/09/21.<br><br>The updated search was done including articles from 2021 until September 28, 2022, and adding the following to, e.g., Medline: (2021/9/2:2022/10/1[pdat]) | |

**Aggregated search string from Medline:**
((("Back"[MeSH Terms] OR "spine"[MeSH Terms] OR "Pelvis"[MeSH Terms] OR ("Back"[Title/Abstract] OR "spine*"[Title/Abstract] OR "spinal*"[Title/Abstract] OR "trunk*"[Title/Abstract] OR "lumbar*"[Title/Abstract] OR "pelvis*"[Title/Abstract] OR "sacrum"[Title/Abstract] OR "lumbo sacral*"[Title/Abstract] OR "lumbosacral*"[Title/Abstract] OR "intervertebral disk*"[Title/Abstract] OR "intervertebral disc*"[Title/Abstract] OR "thoracic vertebrae"[Title/Abstract] OR "thoracic vertebra"[Title/Abstract])) AND ("Pain"[MeSH Terms] OR "Pain Measurement"[MeSH Terms] OR "Cumulative Trauma Disorders"[MeSH Terms] OR "Musculoskeletal Diseases"[MeSH Terms] OR ("Pain"[Title/Abstract] OR "ache*"[Title/Abstract] OR "musculoskeletal disease*"[Title/Abstract] OR "musculoskeletal disorder*"[Title/Abstract] OR "cumulative trauma disorder*"[Title/Abstract] OR "nerve entrapment"[Title/Abstract]))) OR ("Back Pain"[MeSH Terms] OR "Back Injuries"[MeSH Terms] OR "Spinal Diseases"[MeSH Terms] OR "Pelvic Pain"[MeSH Terms] OR "Sciatica"[MeSH Terms] OR ("Back Pain"[Title/Abstract] OR "backache*"[Title/Abstract] OR "back injur*"[Title/Abstract] OR "spinal disease*"[Title/Abstract] OR "spine disease*"[Title/Abstract] OR "spinal injur*"[Title/Abstract] OR "spine injur*"[Title/Abstract] OR "intervertebral disk degeneration"[Title/Abstract] OR "intervertebral disc degeneration"[Title/Abstract] OR "spinal osteochondros*"[Title/Abstract] OR "spine osteochondros*"[Title/Abstract] OR "scheuermann*"[Title/Abstract] OR "spinal stenos*"[Title/Abstract] OR "spondylitis"[Title/Abstract] OR "spondylarthritis"[Title/Abstract] OR "spondylosis"[Title/Abstract] OR "lumbago"[Title/Abstract] OR "Sciatica"[Title/Abstract] OR "Pelvic Pain"[Title/Abstract]))) AND ("Work"[MeSH Terms] OR "Workload"[MeSH Terms] OR "Workplace"[MeSH Terms] OR "Occupations"[MeSH Terms] OR "Occupational Health"[MeSH Terms] OR "Occupational Diseases"[MeSH Terms] OR "Occupational Groups"[MeSH Terms] OR "Occupational Exposure"[MeSH Terms] OR "women, working"[MeSH Terms] OR "Employment"[MeSH Terms:noexp] OR (("work-related"[Title/Abstract] OR "work load*"[Title/Abstract] OR "workload*"[Title/Abstract] OR "workplace*"[Title/Abstract] OR "workplace*"[Title/Abstract] OR "work environment*"[Title/Abstract] OR "working condition*"[Title/Abstract] OR "occupation*"[Title/Abstract] OR "job"[Title/Abstract] OR "employ*"[Title/Abstract]) NOT ("medline"[Filter] OR "oldmedline"[Filter]))) AND ("stress, mechanical"[MeSH Terms] OR "Lifting"[MeSH Terms] OR "Moving and Lifting Patients"[MeSH Terms] OR "Weight-Bearing"[MeSH Terms] OR "Physical Exertion"[MeSH Terms] OR "torsion, mechanical"[MeSH Terms] OR "Postural Balance"[MeSH Terms] OR "Walking"[MeSH Terms] OR "recovery of function"[MeSH Terms] OR "Relaxation"[MeSH Terms] OR ("static"[Title/Abstract] AND ("postural"[All Fields] OR "posturally"[All Fields] OR "posture"[MeSH Terms] OR "posture"[All Fields] OR "postures"[All Fields] OR "postured"[All Fields] OR "posturing"[All Fields])) OR ("awkward"[Title/Abstract] AND ("postural"[All Fields] OR "posturally"[All Fields] OR "posture"[MeSH Terms] OR "posture"[All Fields] OR "postures"[All Fields] OR "postured"[All Fields] OR "posturing"[All Fields])) OR ("dynamic"[Title/Abstract] AND ("postural"[All Fields] OR "posturally"[All Fields] OR "posture"[MeSH Terms] OR "posture"[All Fields] OR "postures"[All Fields] OR "postured"[All Fields] OR "posturing"[All Fields])) OR "static work"[Title/Abstract] OR "dynamic load*"[Title/Abstract] OR "lift*"[Title/Abstract] OR "carry*"[Title/Abstract] OR "hold*"[Title/Abstract] OR "pull*"[Title/Abstract] OR "drag*"[Title/Abstract] OR "push*"[Title/Abstract] OR "manual handling"[Title/Abstract] OR "force*"[Title/Abstract] OR "biomechanic*"[Title/Abstract] OR "walking*"[Title/Abstract] OR "Postural Balance"[Title/Abstract] OR "flexion*"[Title/Abstract] OR "extension*"[Title/Abstract] OR "turning"[Title/Abstract] OR "sitting"[Title/Abstract] OR "kneeling"[Title/Abstract] OR "squatting"[Title/Abstract] OR "twisting"[Title/Abstract] OR "bending"[Title/Abstract] OR "reaching"[Title/Abstract] OR "standing"[Title/Abstract] OR "sedentary"[Title/Abstract] OR "repetitive movement*"[Title/Abstract] OR "monotonous work"[Title/Abstract] OR "Relaxation"[Title/Abstract] OR "recovery of function"[Title/Abstract] OR "physical demand*"[Title/Abstract] OR "physically demand*"[Title/Abstract] OR ("stress, psychological"[MeSH Terms] OR "Social Support"[MeSH Terms] OR "Job Satisfaction"[MeSH Terms] OR "Work Schedule Tolerance"[MeSH Terms] OR "Employee Performance Appraisal"[MeSH Terms] OR "Employee Grievances"[MeSH Terms] OR "social justice/psychology"[MeSH Terms] OR "Personnel Downsizing"[MeSH Terms] OR "Staff Development"[MeSH Terms] OR "Organizational Culture"[MeSH Terms] OR "Bullying"[MeSH Terms] OR "Prejudice"[MeSH Terms] OR "Social Discrimination"[MeSH Terms] OR "Interpersonal Relations"[MeSH Terms] OR "communication/psychology"[MeSH Terms] OR "psychosocial"[Title/Abstract] OR "job strain"[Title/Abstract] OR "work strain"[Title/Abstract] OR "work demand*"[Title/Abstract] OR "job demand*"[Title/Abstract] OR "high demand*"[Title/Abstract] OR "low control"[Title/Abstract] OR "lack of control"[Title/Abstract] OR "work control"[Title/Abstract] OR "job control"[Title/Abstract] OR "decision latitude"[Title/Abstract] OR "work influence*"[Title/Abstract] OR "demand resource*"[Title/Abstract] OR "effort reward*"[Title/Abstract] OR "time pressure*"[Title/Abstract] OR "recuperation*"[Title/Abstract] OR "work overload*"[Title/Abstract] OR "work over load*"[Title/Abstract] OR "recovery"[Title/Abstract] OR "coping"[Title/Abstract] OR "work ability"[Title/Abstract] OR "Social Support"[Title/Abstract] OR "support system*"[Title/Abstract] OR "social network*"[Title/Abstract] OR "emotional support"[Title/Abstract] OR "interpersonal relation*"[Title/Abstract] OR "interaction*"[Title/Abstract] OR "justice*"[Title/Abstract] OR "injustice*"[Title/Abstract] OR "Job Satisfaction"[Title/Abstract] OR "work satisfaction"[Title/Abstract] OR "boredom"[Title/Abstract] OR "skill discretion*"[Title/Abstract] OR "Staff Development"[Title/Abstract] OR "discrimination"[Title/Abstract] OR "harass*"[Title/Abstract] OR (("workplace"[MeSH Terms] OR "workplace"[All Fields] OR ("work"[All Fields] AND "place"[All Fields]) OR "work-place"[All Fields]) AND "conflict*"[Title/Abstract]) OR "workplace violen*"[Title/Abstract] OR "work place violen*"[Title/Abstract] OR "Bullying"[Title/Abstract] OR "victimization*"[Title/Abstract] OR (("silent"[All Fields] OR "silently"[All Fields] OR "silents"[All Fields]) AND "workplace*"[Title/Abstract]) OR "role ambiguity"[Title/Abstract] OR "roleconflict*"[Title/Abstract] OR "work role*"[Title/Abstract] OR "working hour*"[Title/Abstract] OR "working time"[Title/Abstract] OR "daytime"[Title/Abstract] OR "night-time"[Title/Abstract] OR "shift work*"[Title/Abstract] OR "work shift*"[Title/Abstract] OR "temporary work*"[Title/Abstract] OR "full-time"[Title/Abstract] OR "part-time"[Title/Abstract] OR "flexible work*"[Title/Abstract] OR "organizational change"[Title/Abstract] OR "organisational change"[Title/Abstract] OR "lean production"[Title/Abstract] OR "job security"[Title/Abstract] OR "job insecurity"[Title/Abstract]) OR ("Air Pollutants"[MeSH Terms] OR "Air Pollution"[MeSH Terms] OR "Fluids and Secretions"[MeSH Terms] OR "Toxic Actions"[MeSH Terms] OR "Solvents"[MeSH Terms] OR "pollut*"[Title/Abstract] OR "indoor air*"[Title/Abstract] OR "airborne"[Title/Abstract] OR ("smok*"[Title/Abstract] AND "pollut*"[Title/Abstract]) OR "passive smok*"[Title/Abstract] OR "hazardous chemical*"[Title/Abstract] OR "hazardous material*"[Title/Abstract] OR "hazardous substance*"[Title/Abstract] OR "toxic action*"[Title/Abstract] OR "pesticide*"[Title/Abstract] OR "poison*"[Title/Abstract] OR

"solvent*"[Title/Abstract]) OR ("Radiation"[MeSH Terms] OR "air pollution, radioactive"[MeSH Terms] OR "Hot Temperature"[MeSH Terms] OR "Cold Temperature"[MeSH Terms] OR "Climate"[MeSH Terms] OR "radiation*"[Title/Abstract] OR "climate*"[Title/Abstract] OR "cold temperature*"[Title/Abstract] OR "hot temperature*"[Title/Abstract]) OR ("Communicable Diseases"[MeSH Terms] OR "communicable disease*"[Title/Abstract] OR "contagious*"[Title/Abstract]) OR ("Noise"[MeSH Terms] OR "noise*"[Title/Abstract]) OR ("Vibration"[MeSH Terms] OR "Motor Vehicles"[MeSH Terms] OR "Automobile Driving"[MeSH Terms] OR "driving"[Title/Abstract] OR "automobile*"[Title/Abstract] OR "car"[Title/Abstract] OR "cars"[Title/Abstract] OR "vibrat*"[Title/Abstract] OR "vehicle*"[Title/Abstract] OR "truck*"[Title/Abstract] OR "lorry"[Title/Abstract] OR "lorries"[Title/Abstract] OR "buses"[Title/Abstract] OR "hand held tool*"[Title/Abstract] OR "hand tool*"[Title/Abstract] OR "power tool*"[Title/Abstract])) AND ("english"[Language] OR "danish"[Language] OR "norwegian"[Language] OR "swedish"[Language])

# **Appendix B**. PECOS

**Table S2**. Inclusion and exclusion criteria for the systematic review.

| | **Inclusion criteria** | **Exclusion criteria** |
|---|---|---|
| **Population** | Adults in or above working age. | Adults never in work or students. |
| **Exposure** | Occupational psychosocial exposures quantified through self-report, interview, observation, or job-exposure-matrix. | Non-related occupational exposures. |
| **Comparison** | A measure of association between occupational exposures and chronic LBP expressed in an appropriate risk estimate, e.g., odds ratio, relative risk, hazard ratio, or prevalence ratio with corresponding 95% confidence interval. | If no appropriate risk estimate is provided or not possible to calculate based on information eligible in the study. |
| **Outcome** | Non-specific chronic low back pain lasting $\geq$3 months.<br><br>Specific chronic low back pain including lumbago, sciatica, lumbar herniated disc and lumbosacral degenerative changes with or without radiculopathy.<br><br>Outcome measured with self-report, interview, clinical diagnosis (e.g., ICD code), imaging modalities (e.g., computed tomography, x-ray, or magnetic resonance imaging), or compensation/insurance claim. | Injuries based on accidents, inherent pain, pain caused by other diseases or conditions such as cancer, fractures, or inflammation.<br><br>Proxy measures to chronic LBP, e.g., sickness absenteeism.<br><br>Studies not reporting on chronic LBP according to our definition as pain lasting $\geq$3 months. |
| **Study design** | Randomised control trials; non-randomised control trials; prospective cohort; retrospective cohort; case-control; case-cohort; nested case-control.<br><br>Original study in full text and peer reviewed.<br><br>In English, Danish, Swedish or Norwegian.<br><br>Published from January the 10$^{th}$ 2014. | Cross-sectional studies, systematic reviews, in vitro studies, studies on health economics, studies not addressing any risk factors related to work, studies with less than 30 participants, and Studies in other languages than in the inclusion criteria.<br><br>Animal trials.<br><br>Conference notes, books, letters to editor, editorial pages, protocols, reports and abstracts. |

LBP=low back pain; ICD=International Classification of Diseases.

## **Appendix C**. Risk of bias assessment tool.

**Table S3**. Risk of bias assessment tool, case-control studies.

| **Case-Control Study** | **Yes** | **No** | **Unclear** |
|---|---|---|---|
| **Major domain 1 – study design and selection** | | | |
| **Were the cases recruited in an acceptable way?** Consider the following:<br>● Are the cases representative of a population, clearly defined and differentiated from controls?<br>● Was there an established reliable system for selecting all the cases?<br>● Were inclusion and exclusion criteria explicit and applied similarly to all eligible cases? | | | |
| **Were the controls selected in an acceptable way?** Consider the following:<br>● Are the controls representative of a population and clearly defined?<br>● Are the same inclusion and exclusion criteria for cases used to select controls (equally applied) and matched appropriately?<br>● Is it clearly established that controls are non-cases? | | | |
| **Is the participation rate satisfactory?** Consider the following:<br>● Are there large differences between the two groups?<br>● Is the participation rate low? | | | |
| **Major domain 2 – Exposure** | | | |
| **Was the exposure accurately measured to minimise bias?** Consider the following:<br>● Is the exposure clearly defined?<br>● Do measurements truly reflect what it is supposed to measure (have they been validated?).<br>● Is the method of assessment reliable? | | | |
| **Major domain 3 – Outcome** | | | |
| **Was the outcome accurately measured to minimise bias?** Consider the following:<br>● Is the outcome clearly defined?<br>● Do measurements truly reflect what it is supposed to measure (have they been validated?).<br>● Is the method of assessment reliable? | | | |
| **Major domain 4 – Non-participants** | | | |
| **Is comparison made between participants and non-participants?**<br>Consider the following:<br>● Is similarities or differences established? | | | |
| **Major domain 5 – Analysis method** | | | |
| **Was the analysis method adequate?** Consider the following:<br>● Are the main potential confounders identified and taken into account in the analysis?<br>● Were adequate statistical models used to reduce bias? | | | |
| **Minor domain 1 – Funding** | | | |
| **Was the source of funding provided?** Consider the following:<br>● Was the study affected by sponsors?<br>● Did sponsoring organisation participate in the analysis? | | | |
| **Minor domain 2 – Chronology** | | | |
| **Could chronology be established?** Consider the following:<br>● Was the timeframe sufficient to see an association between the exposure and outcome? | | | |
| **Minor domain 3 – Conflict of interest** | | | |
| **Was the study without any conflict of interest?** Consider the following:<br>● Was the study affected by the authors affiliations or interests? | | | |

**Table S4**. Risk of bias assessment tool, cohort studies.

| Cohort Study | Yes | No | Unclear |
|---|---|---|---|
| **Major domain 1 – study design and selection** | | | |
| **Was the cohort recruited in an acceptable way?** Consider the following:<br>● Is it representative of a defined population and clearly specified?<br>● Are groups comparable in all respects other than the factor under investigation?<br>● Was everybody included who should have been? | | | |
| **Was the follow-up of subjects acceptable?** Consider the following:<br>● Conventionally, a 20% drop out rate is acceptable, but observational studies conducted over longer periods, a higher drop-out rate is to be expected.<br>● Were losses to follow-up taken into account in the analysis (sensitivity analysis, described etc.)? | | | |
| **Major domain 2 – Exposure** | | | |
| **Was the exposure accurately measured to minimise bias?** Consider the following:<br>● Is the exposure clearly defined?<br>● Do measurements truly reflect what it is supposed to measure (have they been validated?).<br>● Is the method of assessment reliable?<br>● Were all the subjects classified into exposure groups using the same procedure? | | | |
| **Major domain 3 – Outcome** | | | |
| **Was the outcome accurately measured to minimise bias?** Consider the following:<br>● Is the outcome clearly defined?<br>● Do measurements truly reflect what it is supposed to measure (have they been validated?).<br>● Is the method of assessment reliable?<br>● Were the measurement methods similar in the different groups?<br>● If blinding is not possible, is there some recognition that knowledge of exposure status could influence the assessment of the outcome? | | | |
| **Major domain 4 – Enrolment** | | | |
| **Was the outcome taken into account at enrolment?** Consider the following:<br>● Some participants might have the outcome at the time of enrolment. Is it assessed at baseline in the analysis? | | | |
| **Major domain 5 – Analysis method** | | | |
| **Was the analysis method adequate?** Consider the following:<br>● Are the main potential confounders identified and taken into account in the analysis?<br>● Were adequate statistical models used to reduce bias? | | | |
| **Minor domain 1 – Funding** | | | |
| **Was the source of funding provided?** Consider the following:<br>● Was the study affected by sponsors?<br>● Did sponsoring organization participate in the analysis? | | | |
| **Minor domain 2 – Chronology** | | | |
| **Could chronology be established?** Consider the following:<br>● Was the timeframe sufficient to see an association between the exposure and outcome?<br>● Was the follow-up long enough for the outcome to occur? | | | |
| **Minor domain 3 – Conflict of interest** | | | |
| **Was the study without any conflict of interest?** Consider the following:<br>● Was the study affected by the authors affiliations or interests? | | | |

## **Appendix D**. Grading the evidence of an association

The quality of evidence was evaluated in accordance with the GRADE guidelines, complying with guidelines stated by the Navigation Guide. Therefore, the evidence starts at moderate quality when evaluating observational studies.

The criteria and definitions stated in our instructions has been adopted from the Navigation Guide (Woodruff and Sutton 2014) (1) and Hulshof 2019 (2).

**Table S5.** Quality level - four levels of evidence.

| **Quality level** | **Definition** |
|---|---|
| High ++++ | We are confident that the true effect lies close to that of the estimate of the effect. |
| Moderate +++ | We are moderately confident in the effect estimate: The true effect is likely to be close to the estimate of the effect, but there is a possibility that it is substantially different. |
| Low ++ | Our confidence in the effect estimate is limited: The true effect may be substantially different from the estimate of the effect. |
| Very low + | We have very little confidence in the effect estimate: The true effect is likely to be substantially different from the estimate of effect. |

**Table S6.** Evaluating the quality of evidence - down- and upgrading factors.

| **Downgrading factors** | **Criteria for downgrading.**<br>(0= no change; -1 (serious) or -2 (very serious) = downgrading 1 or 2 levels). |
|---|---|
| Risk of bias | Criteria:<br>Judicious considerations regarding each study's contribution towards the measure of association is warranted. One should be confident that there is a substantial risk of bias across most studies included and not downgrade based on an average count. |
| Inconsistency | Criteria:<br>Consider if:<br>* Effect estimates of the included studies vary widely<br>* Confidence intervals show minimal or no overlap<br>* Heterogeneity (I-squared) is high<br><br>Inconsistency is important when it reduces the confidence in results. Even if inconsistency is large, estimates that, overall, points towards an increased risk would not be downgraded. For instance, if a protective or harmful effect cannot be determined, GRADE suggests downgrading. |
| Indirectness | Criteria:<br>* Do differences occur between populations of interest?<br>* Do differences occur between outcome measures of interest?<br>* Do differences occur between exposure measures of interest? |

| | |
|---|---|
| | * Are differences substantial so that it decreases our confidence in comparisons or make difference in the outcome likely? |
| Imprecision | Criteria:<br>* Do studies have few participants or few events?<br>* Are the confidence intervals wide?<br>* Are few studies included in the pooled estimate?<br>* Confidence interval includes OR/RR of 1.0 |
| Publication bias | Criteria:<br>* Consider Funnel plot and Egger's test<br>* Are there sufficient studies included to interpret the funnel plot accordingly?<br>* In environmental research, consider the likelihood of publishing studies who finds no associations (underestimations might occur). |
| **Upgrading factors** | **Criteria for upgrading.**<br>(0= no change; +1 or +2 = upgrading 1 or 2 levels). |
| Magnitude of effect | Criteria:<br>* Upgrade if a large magnitude of effect exists (GRADE defines it as OR/RR of >2.0)<br>* Assessments should be based on the knowledge of occupational health and expert judgement. |
| Dose response | Criteria:<br>*Do one or more studies show or test and dose(exposure)-response relation? |
| Residual confounding | Criteria:<br>* Upgrade if consideration of all plausible residual confounders, biases, or effect modifications would underestimate the effect when results show no effect. |

(1) Woodruff TJ, Sutton P. The Navigation Guide systematic review methodology: a rigorous and transparent method for translating environmental health science into better health outcomes. Environ Health Perspect. 2014;122(10):1007-14.

(2) Hulshof CTJ, Colosio C, Daams JG, Ivanov ID, Prakash KC, Kuijer PPFM, Leppink N, Mandic-Rajcevic S, Masci F, van der Molen HF, Neupane S, Nygård CH, Oakman J, Pega F, Proper K, Prüss-Üstün AM, Ujita Y, Frings-Dresen MHW. WHO/ILO work-related burden of disease and injury: Protocol for systematic reviews of exposure to occupational ergonomic risk factors and of the effect of exposure to occupational ergonomic risk factors on osteoarthritis of hip or knee and selected other musculoskeletal diseases. Environ Int. 2019 Apr;125:554-566. doi: 10.1016/j.envint.2018.09.053. Epub 2018 Dec 22. PMID: 30583853; PMCID: PMC7794864.

**Table S7**. Results of GRADE.

| No of studies* | Certainty assessment | | | | | | Effect estimate (95% CI) | Certainty |
|---|---|---|---|---|---|---|---|---|
| | Study design | Risk of Bias | Inconsistency | Indirectness | Imprecision | Other considerations | | |
| **Job control** | | | | | | | | |
| 7 | Observational studies | Serious (-1) | Not serious (0) | Not serious (0) | Serious (-1) | - Indication of publication bias (0)<br>- Plausible confounding (0)<br>- Dose-response gradient (0)<br>- Magnitude of effect (0) | OR 1.0 (0.9 to 1.1) | ⊕○○○ Very low |
| **Job demand** | | | | | | | | |
| 4 | Observational studies | Serious (-1) | Not serious (0) | Not serious (0) | Serious (-1) | - Indication of publication bias (0)<br>- Plausible confounding (0)<br>- Dose-response gradient (0)<br>- Magnitude of effect (0) | OR 1.1 (1.0 to 1.2) | ⊕○○○ Very low |
| **Job strain** | | | | | | | | |
| 2 | Observational studies | Serious (-1) | Not serious (0) | Not serious (0) | Very serious (-2) | - Indication of publication bias (0)<br>- Plausible confounding (0)<br>- Dose-response gradient (0)<br>- Magnitude of effect (0) | OR 1.0 (0.7 to 1.6) | ⊕○○○ Very low |
| **Job support** | | | | | | | | |
| 6 | Observational studies | Serious (-1) | Not serious (0) | Not serious (0) | Serious (-1) | - Indication of publication bias (0)<br>- Plausible confounding (0)<br>- Dose-response gradient (0)<br>- Magnitude of effect (0) | OR 0.8 (0.5 to 1.1) | ⊕○○○ Very low |
| **Job stress** | | | | | | | | |
| 5 | Observational studies | Serious (-1) | Serious (-1) | Not serious (0) | Serious (-1) | - Indication of publication bias (0)<br>- Plausible confounding (0)<br>- Dose-response gradient (0)<br>- Magnitude of effect (0) | OR 1.1 (0.6 to 1.8) | ⊕○○○ Very low |
| **Job satisfaction** | | | | | | | | |
| 6 | Observational studies | Serious (-1) | Serious (-1) | Not serious (0) | Serious (-1) | - Indication of publication bias (0)<br>- Plausible confounding (0)<br>- Dose-response gradient (0)<br>- Magnitude of effect (0) | OR 0.9 (0.6 to 1.2) | ⊕○○○ Very low |

*Number of studies included in the meta-analysis after removal of double-counting data.

## **Appendix E**. Excluded articles.

**Table S8**. Excluded articles based on full-text read and reason for exclusion.

| **References: Studies published before January 10, 2014** | | **Explanation for exclusion** |
|---|---|---|
| 1 | Albert HB, Godskesen M, Korsholm L, Westergaard JG. Risk factors in developing pregnancy-related pelvic girdle pain. Acta Obstet Gynecol Scand. 2006;85(5):539-44. | Outcome criteria not fulfilled. |
| 2 | Andersen JH, Haahr JP, Frost P. Risk factors for more severe regional musculoskeletal symptoms: a two-year prospective study of a general working population. Arthritis Rheum 2007;56:1355-64. | Outcome criteria not fulfilled. |
| 3 | Battie MC, Videman T, Gibbons LE, Fisher LD, Manninen H, Gill K. 1995 Volvo Award in clinical sciences. Determinants of lumbar disc degeneration. A study relating lifetime exposures and magnetic resonance imaging findings in identical twins. Spine (Phila Pa 1976) 1995;20:2601-12. | Outcome criteria not fulfilled. |
| 4 | Battie MC, Videman T, Gibbons LE, Manninen H, Gill K, Pope M, et al. Occupational driving and lumbar disc degeneration: a case-control study. Lancet 2002;360:1369-74. | Outcome criteria not fulfilled. |
| 5 | Bergenudd H, Johnell O. Somatic versus nonsomatic shoulder and back pain experience in middle age in relation to body build, physical fitness, bone mineral content, gammaglutamyltransferase, occupational workload, and psychosocial factors. Spine (Phila Pa 1976). 1991;16(9):1051-5. | Outcome criteria not fulfilled. |
| 6 | Biering-Sorensen F. A prospective study of low back pain in a general population. I. Occurrence, recurrence and aetiology. Scand J Rehabil Med. 1983;15(2):71-9. | Outcome criteria not fulfilled. |
| 7 | Biering-Sorensen F, Thomsen C. Medical, social and occupational history as risk indicators for low-back trouble in a general population. Spine (Phila Pa 1976). 1986;11(7):720-5. | Outcome criteria not fulfilled. |
| 8 | Bigos SJ, Battie MC, Spengler DM, Fisher LD, Fordyce WE, Hansson T, et al. A longitudinal, prospective study of industrial back injury reporting. Clinical orthopaedics and related research. 1992(279):21-34. | Outcome criteria not fulfilled. |
| 9 | Bigos SJ, Battie MC, Spengler DM, Fisher LD, Fordyce WE, Hansson TH, et al. A prospective study of work perceptions and psychosocial factors affecting the report of back injury. Spine (Phila Pa 1976). 1991;16(1):1-6. | Outcome criteria not fulfilled. |
| 10 | Bildt C, Alfredsson L, Michélsen H, Punnett L, Vingård E, Torgén M, et al. Occupational and nonoccupational risk indicators for incident and chronic low back pain in a sample of the Swedish general population during a 4-year period: an influence of depression? International Journal of Behavioral Medicine 2000;7:372-92. | Outcome criteria not fulfilled. |
| 11 | Bildt C, Alfredsson L, Punnett L, Theobald H, Torgen M, Wikman A. Effects of drop out in a longitudinal study of musculoskeletal disorders. Occup Environ Med. 2001;58(3):194-9. | Outcome criteria not fulfilled. |
| 12 | Bjorksten MG, Talback M. A follow-up study of psychosocial factors and musculoskeletal problems among unskilled female workers with monotonous work. Eur J Public Health. 2001;11(1):102-8. | Outcome criteria not fulfilled. |
| 13 | Boos N, Rieder R, Schade V, Spratt KF, Semmer N, Aebi M. 1995 Volvo Award in clinical sciences. The diagnostic accuracy of magnetic resonance imaging, work perception, and psychosocial factors in identifying symptomatic disc herniations. Spine (Phila Pa 1976). 1995;20(24):2613-25. | Outcome criteria not fulfilled. |
| 14 | Boos N, Semmer N, Elfering A, Schade V, Gal I, Zanetti M, et al. Natural history of individuals with asymptomatic disc abnormalities in magnetic resonance imaging: predictors of low back pain-related medical consultation and work incapacity. Spine (Phila Pa 1976). 2000;25(12):1484-92. | Outcome criteria not fulfilled. |
| 15 | Boshuizen HC, Bongers PM, Hulshof CT. Self-reported back pain in tractor drivers exposed to whole-body vibration. Int Arch Occup Environ Health 1990;62:109-15. | Outcome criteria not fulfilled. |
| 16 | Bovenzi M. Metrics of whole-body vibration and exposure-response relationship for low back pain in professional drivers: a prospective cohort study. Int Arch Occup Environ Health 2009;82:893-917. | Outcome criteria not fulfilled. |
| 17 | Bovenzi M. A longitudinal study of low back pain and daily vibration exposure in professional drivers. Ind Health 2010;48:584-95. | Outcome criteria not fulfilled. |
| 18 | Bridger RS, Brasher K, Bennett A. Sustaining person-environment fit with a changing workforce. Ergonomics. 2012. | Outcome criteria not fulfilled. |
| 19 | Brynhildsen J, Hansson A, Persson A, Hammar M. Follow-up of patients with low back pain during pregnancy. Obstet Gynecol. 1998;91(2):182-6. | Outcome criteria not fulfilled. |
| 20 | Bugajska J, Zolnierczyk-Zreda D, Jedryka-Goral A, Gasik R, Hildt-Ciupinska K, Malinska M, et al. Psychological factors at work and musculoskeletal disorders: a one year prospective study. Rheumatol Int 2013;33:2975-83. | Outcome criteria not fulfilled. |
| 21 | Burdorf A, Jansen JP. Predicting the long term course of low back pain and its consequences for sickness absence and associated work disability. Occup Environ Med 2006;63:522-9. | Outcome criteria not fulfilled. |
| 22 | Canivet C, Ostergren PO, Choi B, Nilsson P, af Sillen U, Moghadassi M, et al. Sleeping problems as a risk factor for subsequent musculoskeletal pain and the role of job strain: results from a one-year follow-up of the Malmo Shoulder Neck Study Cohort. International journal of behavioral medicine. 2008;15(4):254-62. | Outcome criteria not fulfilled. |
| 23 | Christensen JO, Knardahl S. Work and back pain: a prospective study of psychological, social and mechanical predictors of back pain severity. Eur J Pain 2012;16:921-33. | Outcome criteria not fulfilled. |

| | | |
|---|---|---|
| 24 | Clausen T, Andersen LL, Holtermann A, Jorgensen AF, Aust B, Rugulies R. Do self-reported psychosocial working conditions predict low back pain after adjustment for both physical work load and depressive symptoms? A prospective study among female eldercare workers. Occup Environ Med 2013; 70:538-44. | Outcome criteria not fulfilled. |
| 25 | Clays E, De Bacquer D, Leynen F, Kornitzer M, Kittel F, De Backer G. The impact of psychosocial factors on low back pain: longitudinal results from the Belstress study. Spine (Phila Pa 1976). 2007;32(2):262-8. | Outcome criteria not fulfilled. |
| 26 | Coenen P, Kingma I, Boot CR, Twisk JW, Bongers PM, van Dieen JH. Cumulative low back load at work as a risk factor of low back pain: a prospective cohort study. J Occup Rehabil 2013;23:11-8. | Outcome criteria not fulfilled. |
| 27 | Coupland CA, Grainge MJ, Cliffe SJ, Hosking DJ, Chilvers CE. Occupational activity and bone mineral density in postmenopausal women in England. Osteoporos Int. 2000;11(4):310-5. | Outcome criteria not fulfilled. |
| 28 | Daltroy LH, Larson MG, Wright EA, Malspeis S, Fossel AH, Ryan J, et al. A casecontrol study of risk factors for industrial low back injury: implications for primary and secondary prevention programs. Am J Ind Med. 1991;20(4):505-15. | Outcome criteria not fulfilled. |
| 29 | de Zwart BC, Broersen JP, van der Beek AJ, Frings-Dresen MH, Van Dijk FJ. Selection related to musculoskeletal complaints among employees. Occup Environ Med. 1997;54(11):800-6. | Outcome criteria not fulfilled. |
| 30 | de Zwart BC, Broersen JP, Frings-Dresen MH, van Dijk FJ. Repeated survey on changes in musculoskeletal complaints relative to age and work demands. Occup Environ Med. 1997;54(11):793-9. | Outcome criteria not fulfilled. |
| 31 | Elders LA, Burdorf A. Prevalence, incidence, and recurrence of low back pain in scaffolders during a 3-year follow-up study. Spine (Phila Pa 1976) 2004;29:E101-6. | Outcome criteria not fulfilled. |
| 32 | Elfering A, Grebner S, Semmer NK, Gerber H. Time control, catecholamines and back pain among young nurses. Scand J Work Environ Health 2002;28:386-93. | Outcome criteria not fulfilled. |
| 33 | Elfering A, Mannion AF, Jacobshagen N, Tamcan O, Muller U. Beliefs about back pain predict the recovery rate over 52 consecutive weeks. Scand J Work Environ Health. 2009;35(6):437-45. | Outcome criteria not fulfilled. |
| 34 | Elfering A, Semmer N, Birkhofer D, Zanetti M, Hodler J, Boos N. Risk factors for lumbar disc degeneration: a 5-year prospective MRI study in asymptomatic individuals. Spine (Phila Pa 1976). 2002;27(2):125-34. | No measure of an association. |
| 35 | Elfering A, Semmer NK, Schade V, Grund S, Boos N. Supportive colleague, unsupportive supervisor: the role of provider-specific constellations of social support at work in the development of low back pain. Journal of occupational health psychology. 2002;7(2):130-40. | Outcome criteria not fulfilled. |
| 36 | Engkvist IL. Back injuries among nurses - A comparison of the accident processes after a 10-year follow-up. Safety Science. 2008;46(2):291-301. | Outcome criteria not fulfilled. |
| 37 | Engkvist IL, Hagberg M, Hjelm EW, Menckel E, Ekenvall L. The accident process preceding overexertion back injuries in nursing personnel. PROSA study group. Scand J Work Environ Health. 1998;24(5):367-75. | Outcome criteria not fulfilled. |
| 38 | Engkvist IL, Hjelm EW, Hagberg M, Menckel E, Ekenvall L. Risk indicators for reported over-exertion back injuries among female nursing personnel. Epidemiology (Cambridge, Mass). 2000;11(5):519-22. | Outcome criteria not fulfilled. |
| 39 | Eriksen W, Natvig B, Bruusgaard D. Smoking, heavy physical work and low back pain: a four-year prospective study. Occup Med (Lond). 1999;49(3):155-60. | Outcome criteria not fulfilled. |
| 40 | Eriksen W, Bruusgaard D, Knardahl S. Work factors as predictors of intense or disabling low back pain; a prospective study of nurses' aides. Occup Environ Med 2004;61:398-404. | Outcome criteria not fulfilled. |
| 41 | Ferguson SA, Allread WG, Burr DL, Heaney C, Marras WS. Biomechanical, psychosocial and individual risk factors predicting low back functional impairment among furniture distribution employees. Clinical Biomechanics. 2012;27(2):117-23. | Outcome criteria not fulfilled. |
| 42 | Ferguson SA, Marras WS, Burr DL. The influence of individual low back health status on workplace trunk kinematics and risk of low back disorder. Ergonomics. 2004;47(11):1226-37. | Outcome criteria not fulfilled. |
| 43 | Feyer AM, Herbison P, Williamson AM, de Silva I, Mandryk J, Hendrie L, et al. The role of physical and psychological factors in occupational low back pain: a prospective cohort study. Occup Environ Med 2000;57:116-20. | Outcome criteria not fulfilled. |
| 44 | Gatchel RJ, Polatin PB, Mayer TG. The dominant role of psychosocial risk factors in the development of chronic low back pain disability. Spine (Phila Pa 1976). 1995;20(24):2702-9. | Outcome criteria not fulfilled. |
| 45 | Gheldof EL, Vinck J, Vlaeyen JW, Hidding A, Crombez G. Development of and recovery from short- and long-term low back pain in occupational settings: a prospective cohort study. Eur J Pain 2007;11:841-54. | Outcome criteria not fulfilled. |
| 46 | Gnudi S, Sitta E, Gnudi F, Pignotti E. Relationship of a lifelong physical workload with physical function and low back pain in retired women. Aging Clinical & Experimental Research. 2009;21(1):55-61. | Outcome criteria not fulfilled. |
| 47 | Gonge H, Jensen LD, Bonde JP. Do psychosocial strain and physical exertion predict onset of low-back pain among nursing aides? Scand J Work Environ Health 2001;27:388-94. | Outcome criteria not fulfilled. |
| 48 | Gonge H, Jensen LD, Bonde JP. Are psychosocial factors associated with low-back pain among nursing personnel? Work & Stress 2002;16:79-87. | Outcome criteria not fulfilled. |
| 49 | Hagberg M, Vilhemsson R, Tornqvist EW, Toomingas A. Incidence of self-reported reduced productivity owing to musculoskeletal symptoms: association with workplace and individual factors among computer users. Ergonomics 2007;50:1820-34. | Outcome criteria not fulfilled. |
| 50 | Hakkanen M, Viikari-Juntura E, Martikainen R. Incidence of musculo-skeletal disorders among newly em-ployed manufacturing workers. Scand J Work Environ Health 2001;27:381-7. | Outcome criteria not fulfilled. |

| | | |
|---|---|---|
| 51 | Hamberg-van Reenen HH, Ariens GA, Blatter BM, van der Beek AJ, Twisk JW, van Mechelen W, et al. Is an imbalance between physical capacity and exposure to work-related physical factors associated with low-back, neck or shoulder pain? Scand J Work Environ Health 2006;32:190-7. | Outcome criteria not fulfilled. |
| 52 | Harkness EF, Macfarlane GJ, Nahit ES, Silman AJ, McBeth J. Risk factors for new-onset low back pain amongst cohorts of newly employed workers. Rheumatology (Oxford) 2003;42: 959-68. | Outcome criteria not fulfilled. |
| 53 | Hartvigsen J, Bakketeig LS, Leboeuf- Yde C, Engberg M, Lauritzen T. The association between physical workload and low back pain clouded by the ”healthy worker” effect: population-based cross-sectional and 5-year pro-spective questionnaire study. Spine (Phila Pa 1976) 2001;26:1788-92; discussion 1792-3. | Outcome criteria not fulfilled. |
| 54 | Hartvigsen J, Bakketeig LS, Leboeuf-Yde C, Engberg M, Lauritzen T. [The association between physical workload and low back pain clouded by the "healthy worker" effect]. Ugeskr Laeger. 2002;164(21):2765-8. | Outcome criteria not fulfilled. |
| 55 | Hellsing A, Bryngelsson I. Predictors of musculoskeletal pain in men: a twenty-year follow-up from examination at enlistment. Spine (Phila Pa 1976). 2000;25(23):3080-6. | Outcome criteria not fulfilled. |
| 56 | Holmberg S, Thelin A, Stiernstrom EL, Svardsudd K. The impact of physical work exposure on musculoskeletal symptoms among farmers and rural non-farmers. A population-based study. Annals of Agricultural and Environmental Medicine. 2003;10(2):179-84. | Outcome criteria not fulfilled. |
| 57 | Holmberg S, Thelin A, Stiernstrom EL, Svardsudd K. Low back pain comorbidity among male farmers and rural referents: a population-based study. Ann Agric Environ Med. 2005;12(2):261-8. | Outcome criteria not fulfilled. |
| 58 | Holtermann A, Blangsted AK, Hansen K, Christensen H, Sogaard K. What characterizes cleaners sustaining good musculoskeletal health after years with physically heavy work? International Archives of Occupational and Environmental Health. 2009;82(8):1015-22. | Outcome criteria not fulfilled. |
| 59 | Holtermann A, Clausen T, Aust B, Mortensen OS, Andersen LL. Risk for low back pain from different frequencies, load mass and trunk postures of lifting and carrying among female healthcare workers. Int Arch Occup Environ Health. 2012. | Outcome criteria not fulfilled. |
| 60 | Holtermann A, Clausen T, Aust B, Mortensen OS, Andersen LL. Does occupational lifting and carrying among female health care workers contribute to an escalation of pain-day frequency? Eur J Pain. 2013;17(2):290-6. | Outcome criteria not fulfilled. |
| 61 | Holtermann A, Clausen T, Jørgensen MB, Burdorf A, Andersen LL. Patient handling and risk for developing persistent low-back pain among female healthcare workers. Scand J Work Environ Health 2013;39:164-9. | Outcome criteria not fulfilled. |
| 62 | Hooftman WE, van der Beek AJ, Bongers PM, van Mechelen W. Is there a gender difference in the effect of work-related physical and psycho-social risk factors on musculoskeletal symptoms and related sickness absence? Scand J Work Environ Health 2009;35:85-95. | Outcome criteria not fulfilled. |
| 63 | Hoogendoorn WE, Bongers PM, de Vet HC, Douwes M, Koes BW, Miedema MC, et al. Flexion and rotation of the trunk and lifting at work are risk factors for low back pain: results of a prospective cohort study. Spine (Phila Pa 1976) 2000;25:3087-92. | Outcome criteria not fulfilled. |
| 64 | Hoogendoorn WE, Bongers PM, de Vet HC, Houtman IL, Ariens GA, van Mechelen W, et al. Psychosocial work characteristics and psychological strain in relation to low-back pain. Scand J Work Environ Health 2001;27:258-67. | Outcome criteria not fulfilled. |
| 65 | Hoogendoorn WE, Bongers PM, de Vet HC, Twisk JW, van Mechelen W, Bouter LM. Comparison of two different approaches for the analysis of data from a prospective cohort study: an application to work related risk factors for low back pain. Occup Environ Med 2002;59:459-65. | Outcome criteria not fulfilled. |
| 66 | Hoozemans MJ, van der Beek AJ, Fring-Dresen MH, van der Woude LH, van Dijk FJ. Low-back and shoulder complaints among workers with pushing and pulling tasks. Scand J Work Environ Health 2002;28:293-303. | Outcome criteria not fulfilled. |
| 67 | Hultman G, Nordin M, Saraste H. Physical and psychological workload in men with and without low back pain. Scand J Rehabil Med 1995;27:11-7. | Study design criteria not fulfilled. |
| 68 | Hägg O, Fritzell P, Nordwall A. Characteristics of patients with chronic low back pain selected for surgery: a comparison with the general population reported from the Swedish Lumbar Spine Study including commentary by Fanuele J. Spine (Phila Pa 1976). 2002;27(11):1223-31. | Exposure criteria not fulfilled. |
| 69 | Jensen A, Kaerlev L, Tuchsen F, Hannerz H, Dahl S, Nielsen PS, et al. Locomotor diseases among male long-haul truck drivers and other professional drivers. Int Arch Occup Environ Health. 2008;81(7):821-7. | Outcome criteria not fulfilled. |
| 70 | Jensen JC, Haahr JP, Frost P, Andersen JH. Do work-related factors affect care-seeking in general practice for back pain or upper extremity pain? Int Arch Occup Environ Health. 2012. | Outcome criteria not fulfilled. |
| 71 | Jensen JN, Holtermann A, Clausen T, Mortensen OS, Carneiro IG, Andersen LL. The greatest risk for low-back pain among newly educated female health care workers; body weight or physical work load? BMC Musculoskelet Disord 2012;13:87. | Outcome criteria not fulfilled. |
| 72 | Johnston JM, Landsittel DP, Nelson NA, Gardner LI, Wassell JT. Stressful psychosocial work environment in-creases risk for back pain among retail material handlers. Am J Ind Med 2003;43:179-87. | Outcome criteria not fulfilled. |
| 73 | Jorgensen MB, Nabe-Nielsen K, Clausen T, Holtermann A. Independent effect of physical workload and childhood socioeconomic status on low back pain among health care workers in Denmark. Spine (Phila Pa 1976) 2013;38:E359-66. | Outcome criteria not fulfilled. |
| 74 | Josephson M, Ahlberg G, Harenstam A, Svensson H, Theorell T, Wiktorin C, et al. Paid and unpaid work, and its relation to low back and neck/shoulder disorders among women. Women & health. 2003;37(2):17-30. | Outcome criteria not fulfilled. |
| 75 | Josephson M, Hagberg M, Hjelm EW. Self-reported physical exertion in geriatric care. A risk indicator for low back symptoms? Spine (Phila Pa 1976) 1996;21:2781-5. | Outcome criteria not fulfilled. |
| 76 | Josephson M, Lagerstrom M, Hagberg M, Wigaeus Hjelm E. Musculoskeletal symptoms and job strain among nursing personnel: a study over a three year period. Occup Environ Med. 1997;54(9):681-5. | Outcome criteria not fulfilled. |

| | | |
|---|---|---|
| 77 | Josephson M, Vingard E. Workplace factors and care seeking for low-back pain among female nursing personnel. MUSIC-Norrtalje Study Group. Scand J Work Environ Health 1998;24: 465-72. | Outcome criteria not fulfilled. |
| 78 | Juhl M, Andersen PK, Olsen J, Andersen AM. Psychosocial and physical work environment, and risk of pelvic pain in pregnancy. A study within the Danish national birth cohort. J Epidemiol Community Health. 2005;59(7):580-5. | Outcome criteria not fulfilled. |
| 79 | Juul-Kristensen B, Sogaard K, Stroyer J, Jensen C. Computer users' risk factors for developing shoulder, elbow and back symptoms. Scand J Work Environ Health 2004;30:390-8. | Outcome criteria not fulfilled. |
| 80 | Kaaria SM, Malkia EA, Luukkonen RA, Leino-Arjas PI. Pain and clinical findings in the low back: a study of industrial employees with 5-, 10-, and 28-year follow-ups. Eur J Pain. 2010;14(7):759-63. | Outcome criteria not fulfilled. |
| 81 | Kaaria S, Leino-Arjas P, Rahkonen O, Lahti J, Lahelma E, Laaksonen M. Risk factors of sciatic pain: a prospective study among middle-aged employees. Eur J Pain 2011;15:584-90. | Study design criteria not fulfilled. |
| 82 | Kaila-Kangas L, Kivimaki M, Riihimaki H, Luukkonen R, Kirjonen J, Leino-Arjas P. Psychosocial factors at work as predictors of hospitalization for back disorders: a 28-year follow-up of industrial employees. Spine (Phila Pa 1976) 2004;29:1823-30. | Outcome criteria not fulfilled. |
| 83 | Keeney BJ, Turner JA, Fulton-Kehoe D, Wickizer TM, Chan KC, Franklin GM. Early predictors of occupational back reinjury: results from a prospective study of workers in Washington State. Spine (Phila Pa 1976). 2013;38(2):178-87. | Outcome criteria not fulfilled. |
| 84 | Kelsey JL, Githens PB, White AA, 3rd, Holford TR, Walter SD, O'Connor T, et al. An epidemiologic study of lifting and twisting on the job and risk for acute prolapsed lumbar intervertebral disc. Journal of orthopaedic research : official publication of the Orthopaedic Research Society. 1984;2(1):61-6. | Outcome criteria not fulfilled. |
| 85 | Kerr MS, Frank JW, Shannon HS, Norman RW, Wells RP, Neumann WP, et al. Biomechanical and psycho-social risk factors for low back pain at work. Am J Public Health 2001;91: 1069-75. | Outcome criteria not fulfilled. |
| 86 | Kim IH, Geiger-Brown J, Trinkoff A, Muntaner C. Physically demanding workloads and the risks of musculo-skeletal disorders in homecare workers in the USA. Health Soc Care Community 2010;18:445-55. | Outcome criteria not fulfilled. |
| 87 | Kishi R, Doi R, Fukuchi Y, Satoh H, Satoh T, Ono A, et al. Subjective symptoms and neurobehavioral performances of ex-mercury miners at an average of 18 years after the cessation of chronic exposure to mercury vapor. Mercury Workers Study Group. Environmental research. 1993;62(2):289-302. | Outcome criteria not fulfilled. |
| 88 | Knox JB, Orchowski JR, Scher DL, Owens BD, Burks R, Belmont PJ, Jr. Occupational driving as a risk factor for low back pain in active-duty military service members. Spine J. 2013. | Outcome criteria not fulfilled. |
| 89 | Kopec JA, Sayre EC. Work-related psychosocial factors and chronic pain: a prospective cohort study in Canadian workers. J Occup Environ Med 2004; 46:1263-71. | Outcome criteria not fulfilled. |
| 90 | Kopec JA, Sayre EC, Esdaile JM. Predictors of back pain in a general population cohort. Spine (Phila Pa 1976) 2003;29:70-7; discussion 77-8. | Outcome criteria not fulfilled. |
| 91 | Kraus JF, Schaffer KB, McArthur DL, Peek-Asa C. Epidemiology of acute low back injury in employees of a large home improvement retail company. Am J Epidemiol 1997;146:637-45. | Outcome criteria not fulfilled. |
| 92 | Krause N, Ragland DR, Fisher JM, Syme SL. Psychosocial job factors, physical workload, and incidence of work-related spinal injury: a 5-year prospective study of urban transit operators. Spine (Phila Pa 1976) 1998;23:2507-16. | Outcome criteria not fulfilled. |
| 93 | Krause N, Rugulies R, Ragland DR, Syme, SL. Physical workload, ergonomic problems, and incidence of low back injury: a 7.5-year prospective study of San Francisco transit operators. Am J Ind Med 2004 Vol. 46 Issue 6 Pages 570-85. | Exposure criteria not fulfilled. |
| 94 | Kucera KL, Loomis D, Lipscomb HJ, Marshall SW, Mirka GA, Daniels JL. Ergonomic risk factors for low back pain in North Carolina crab pot and gill net commercial fishermen. Am J Ind Med 2009;52:311-21. | Outcome criteria not fulfilled. |
| 95 | Kuh DJ, Coggan D, Mann S, Cooper C, Yusuf E. Height, occupation and back pain in a national prospective study. Br J Rheumatol. 1993;32(10):911-6. | No measure of an association. |
| 96 | Kuijer PP, van der Beek AJ, van Dieen JH, Visser B, Frings-Dresen MH. Effect of job rotation on need for recovery, musculoskeletal complaints, and sick leave due to musculoskeletal complaints: a prospective study among refuse collectors. Am J Ind Med. 2005;47(5):394-402. | Outcome criteria not fulfilled. |
| 97 | Kujala UM, Taimela S, Viljanen T, Jutila H, Viitasalo JT, Videman T, et al. Physical loading and performance as predictors of back pain in healthy adults. A 5-year prospective study. Eur J Appl Physiol Occup Physiol 1996;73:452-8. | Outcome criteria not fulfilled. |
| 98 | Lapointe J, Dionne CE, Brisson C, Montreuil S. Interaction between postural risk factors and job strain on self-reported musculoskeletal symptoms among users of video display units: a three-year prospective study. Scand J Work Environ Health 2009;35:134-44. | Outcome criteria not fulfilled. |
| 99 | Larsman P, Johansson Hanse J. The impact of decision latitude, psychological load and social support at work on the development of neck, shoulder and low back symptoms among female human service organization workers. International Journal of Industrial Ergonomics 2009;39:442-6. | Outcome criteria not fulfilled. |
| 100 | Latza U, Karmaus W, Sturmer T, Steiner M, Neth A, Rehder U. Cohort study of occupational risk factors of low back pain in construction workers. Occup Environ Med 2000;57:28-34. | Outcome criteria not fulfilled. |
| 101 | Lau B, Knardahl S. Perceived job insecurity, job predictability, personality, and health. J Occup Environ Med. 2008;50(2):172-81. | Outcome criteria not fulfilled. |
| 102 | Leclerc A, Tubach F, Landre MF, Ozguler A. Personal and occupational predictors of sciatica in the GAZEL cohort. Occup Med (Lond) 2003;53:384-91. | Outcome criteria not fulfilled. |

| | | |
|---|---|---|
| 103 | Leino PI, Hanninen V. Psychosocial factors at work in relation to back and limb disorders. Scand J Work Environ Health 1995;21:134-42. | Outcome criteria not fulfilled. |
| 104 | Lin MY, Ahern JE, Gershon RR, Grimes M. The use of total quality improvement techniques to determine risk factors for back injuries in hospital workers. Clinical performance and quality health care. 1998;6(1):23-7. | Outcome criteria not fulfilled. |
| 105 | Linton SJ. Do psychological factors increase the risk for back pain in the general population in both a cross-sectional and prospective analysis? Eur J Pain 2005;9:355-61. | Outcome criteria not fulfilled. |
| 106 | Lipscomb HJ, Loomis D, McDonald MA, Kucera K, Marshall S, Li L. Musculoskeletal symptoms among commercial fishers in North Carolina. Appl Ergon. 2004;35(5):417- 26. | Outcome criteria not fulfilled. |
| 107 | Lonnberg F, Pedersen PA, Siersma V. Early predictors of the long-term outcome of low back pain--results of a 22-year prospective cohort study. Family practice. 2010;27(6):609-14. | Outcome criteria not fulfilled. |
| 108 | Lotters F, Burdorf A, Kuiper J, Miedema H. Model for the work-relatedness of lowback pain. Scand J Work Environ Health. 2003;29(6):431-40. | Study design criteria not fulfilled. |
| 109 | Macfarlane GJ, Thomas E, Papageorgiou AC, Croft PR, Jayson MI, Silman AJ. Employment and physical work activities as predictors of future low back pain. Spine (Phila Pa 1976) 1997;22:1143-9. | Outcome criteria not fulfilled. |
| 110 | Manninen P, Heliovaara M, Riihimakl H, Makela P. Does psychological distress predict disability? Int J Epidemiol. 1997;26(5):1063-70. | Outcome criteria not fulfilled. |
| 111 | Manninen P, Riihimak H, Heliovaara M. Incidence and risk factors of low-back pain in middle-aged farmers. Occup Med (Lond) 1995;45:141-6. | Outcome criteria not fulfilled. |
| 112 | Marras WS, Lavender SA, Ferguson SA, Splittstoesser RE, Yang G. Quantitative dynamic measures of physical exposure predict low back functional impairment. Spine (Phila Pa 1976). 2010;35(8):914-23. | Outcome criteria not fulfilled. |
| 113 | Massaccesi M, Pagnotta A, Soccetti A, Masali M, Masiero C, Greco F. Investigation of work-related disorders in truck drivers using RULA method. Appl Ergon. 2003;34(4):303-7. | Outcome criteria not fulfilled. |
| 114 | Masset DF, Piette AG, Malchaire JB. Relation between functional characteristics of the trunk and the occurrence of low back pain. Associated risk factors. Spine (Phila Pa 1976). 1998;23(3):359-65. | Exposure criteria not fulfilled. |
| 115 | Matsudaira K, Kawaguchi M, Isomura T, Arisaka M, Fujii T, Takeshita K, et al. Identification of risk factors for new-onset sciatica in Japanese workers: findings from the Japan epidemiological research of occupation-related back pain study. Spine (Phila Pa 1976) 2013;38:E1691-700. | Outcome criteria not fulfilled. |
| 116 | Matsudaira K, Konishi H, Miyoshi K, Isomura T, Takeshita K, Hara N, et al. Potential risk factors for new onset of back pain disability in Japanese workers: findings from the Japan epidemi-ological research of occupation-related back pain study. Spine (Phila Pa 1976) 2012;37:1324-33. | Outcome criteria not fulfilled. |
| 117 | Melloh M, Elfering A, Stanton TR, Kaser A, Salathe CR, Barz T et al. Who is likely to develop persistent low back pain? A longitudinal analysis of prognostic occupational factors. Work 2013 Vol. 46 Issue 3 Pages 297-311. | Other reasons. |
| 118 | Messing K, Stock SR, Tissot F. Should studies of risk factors for musculoskeletal disorders be stratified by gender? Lessons from the 1998 Quebec Health and Social Survey. Scand J Work Environ Health. 2009;35(2):96-112. | Study design criteria not fulfilled. |
| 119 | Miedema HS, Chorus AM, Wevers CW, van der Linden S. Chronicity of back problems during working life. Spine (Phila Pa 1976). 1998;23(18):2021-8; discussion 8-9. | Outcome criteria not fulfilled. |
| 120 | Mikkonen P, Viikari-Juntura E, Remes J, Pienimäki T, Solovieva S, Taimela S, et al. Physical workload and risk of low back pain in adolescence. Occup Environ Med 2012;69:284-90. | Outcome criteria not fulfilled. |
| 121 | Milosavljevic S, Bagheri N, Vasiljev RM, McBride DI, Rehn B. Does daily exposure to whole-body vibration and mechanical shock relate to the prevalence of low back and neck pain in a rural workforce? Ann Occup Hyg. 2012;56(1):10-7. | Outcome criteria not fulfilled. |
| 122 | Miranda H, Viikari-Juntura E, Martikainen R, Takala EP, Riihimaki H. Individual factors, occupational loading, and physical exercise as pre-dictors of sciatic pain. Spine (Phila Pa 1976) 2002;27:1102-9. | Outcome criteria not fulfilled. |
| 123 | Miranda H, Viikari-Juntura E, Punnett L, Riihimaki H. Occupational loading, health behavior and sleep disturbance as predictors of low-back pain. Scand J Work Environ Health 2008;34:411-9. | Outcome criteria not fulfilled. |
| 124 | Miwa S, Yokogawa A, Kobayashi T, Nishimura T, Igarashi K, Inatani H, et al. Risk factors of recurrent lumbar disc herniation: A single center study and review of the literature. Journal of Spinal Disorders and Techniques. 2012;14. | Outcome criteria not fulfilled. |
| 125 | Myers AH, Baker SP, Li G, Smith GS, Wiker S, Liang KY, et al. Back injury in municipal workers: a case-control study. Am J Public Health 1999;89: 1036-41. | Outcome criteria not fulfilled. |
| 126 | Nahit ES, Hunt IM, Lunt M, Dunn G, Silman AJ, Macfarlane GJ. Effects of psychosocial and individual psychological factors on the onset of musculoskeletal pain: common and site-specific effects. Ann Rheum Dis 2003;62:755-60. | Outcome criteria not fulfilled. |
| 127 | Neumann WP, Wells RP, Norman RW, Frank J, Shannon H, Kerr MS. A posture and load sampling approach to determining low-back pain risk in occupational settings. International Journal of Industrial Ergonomics 2001;27:65-77. | Outcome criteria not fulfilled. |
| 128 | Nuwayhid IA, Stewart W, Johnson JV. Work activities and the onset of first-time low back pain among New York City fire fighters. Am J Epidemiol. 1993;137(5):539-48. | Outcome criteria not fulfilled. |
| 129 | Nyman T, Mulder M, Iliadou A, Svartengren M, Wiktorin C. Physical workload, low back pain and neck-shoulder pain: a Swedish twin study. Occupational and Environmental Medicine [Internet]. 2009; (6):[395-401 pp.]. | No measure of an association. |

| | | |
|---|---|---|
| 130 | Oleske DM, Lavender SA, Andersson GB, Morrissey MJ, Zold-Kilbourn P, Allen C, et al. Risk factors for recurrent episodes of work-related low back dis-orders in an industrial population. Spine (Phila Pa 1976) 2006;31:789-98. | Outcome criteria not fulfilled. |
| 131 | Palmer KT, Griffin M, Ntani G, Shambrook J, McNee P, Sampson M, et al. Professional driving and prolapsed lumbar intervertebral disc diagnosed by magnetic resonance imaging: a case-control study. Scand J Work Environ Health. 2012;38(6):577-81. | Outcome criteria not fulfilled. |
| 132 | Palmer KT, Harris CE, Griffin MJ, Bennett J, Reading I, Sampson M, et al. Casecontrol study of low-back pain referred for magnetic resonance imaging, with special focus on whole-body vibration. Scand J Work Environ Health. 2008;34(5):364-73. | Outcome criteria not fulfilled. |
| 133 | Papageorgiou AC, Croft PR, Thomas E, Silman AJ, Macfarlane GJ. Psychosocial risks for low back pain: are these related to work? Ann Rheum Dis. 1998;57(8):500-2. | Outcome criteria not fulfilled. |
| 134 | Papageorgiou AC, Macfarlane GJ, Thomas E, Croft PR, Jayson MI, Silman AJ. Psychosocial factors in the workplace – do they predict new episodes of low back pain? Evidence from the South Manchester Back Pain Study. Spine (Phila Pa 1976) 1997;22:1137-42. | Outcome criteria not fulfilled. |
| 135 | Pietri F, Leclerc A, Boitel L, Chastang JF, Morcet JF, Blondet M. Low-back pain in commercial travelers. Scand J Work Environ Health 1992;18:52-8. | Outcome criteria not fulfilled. |
| 136 | Piterman L, Dunt D. Occupational lower-back injuries in a primary medical care setting: a five-year follow-up study. Med J Aust. 1987;147(6):276-9. | Outcome criteria not fulfilled. |
| 137 | Plouvier S, Leclerc A, Chastang JF, Bonenfant S, Goldberg M. Socioeconomic position and low-back pain--the role of biomechanical strains and psychosocial work factors in the GAZEL cohort. Scand J Work Environ Health. 2009;35(6):429-36. | Outcome criteria not fulfilled. |
| 138 | Plouvier S, Renahy E, Chastang JF, Bonenfant S, Leclerc A. Biomechanical strains and low back disorders: quantifying the effects of the number of years of exposure on various types of pain. Occup Environ Med. 2008;65(4):268-74. | Outcome criteria not fulfilled. |
| 139 | Power C, Frank J, Hertzman C, Schierhout G, Li L. Predictors of low back pain onset in a prospective British study. Am J Public Health. 2001;91(10):1671-8. | Outcome criteria not fulfilled. |
| 140 | Punnett L, Fine LJ, Keyserling WM, Herrin GD, Chaffin DB. Back dis-orders and nonneutral trunk postures of automobile assembly workers. Scand J Work Environ Health 1991;17:337-46. | Outcome criteria not fulfilled. |
| 141 | Ramond-Roquin A, Bodin J, Serazin C, Parot-Schinkel E, Ha C, Richard I, et al. Biomechanical constraints remain major risk factors for low back pain. Results from a prospective cohort study in French male employees. Spine J 2013 Jul 12 [Epub ahead of print]. | Outcome criteria not fulfilled. |
| 142 | Reigo T, Tropp H, Timpka T. Absence of back disorders in adults and work-related predictive factors in a 5-year perspective. Eur Spine J. 2001;10(3):215-20; discussion 21. | Outcome criteria not fulfilled. |
| 143 | Reme SE, Shaw WS, Steenstra IA, Woiszwillo MJ, Pransky G, Linton SJ. Distressed, immobilized, or lacking employer support? A sub-classification of acute work-related low back pain. J Occup Rehabil 2012;22:541-52. | Outcome criteria not fulfilled. |
| 144 | Riihimaki H, Viikari-Juntura E, Moneta G, Kuha J, Videman T, Tola S. Incidence of sciatic pain among men in machine operating, dynamic physical work, and sedentary work. A three-year follow-up. Spine (Phila Pa 1976). 1994;19(2):138-42. | Exposure criteria not fulfilled. |
| 145 | Riihimaki H, Wickstrom G, Hanninen K, Luopajarvi T. Predictors of sciatic pain among concrete reinforcement workers and house painters--a five-year follow-up. Scand J Work Environ Health. 1989;15(6):415-23. | Outcome criteria not fulfilled. |
| 146 | Riyazi N, Rosendaal FR, Slagboom E, Kroon HM, Breedveld FC, Kloppenburg M. Risk factors in familial osteoarthritis: the GARP sibling study. Osteoarthritis Cartilage. 2008;16(6):654-9. | Outcome criteria not fulfilled. |
| 147 | Rohrer MH, Santos-Eggimann B, Paccaud F, Haller-Maslov E. Epidemiologic study of low back pain in 1398 Swiss conscripts between 1985 and 1992. Eur Spine J. 1994;3(1):2-7. | No measure of an association. |
| 148 | Roy TC, Lopez HP, Piva SR. Loads Worn by Soldiers Predict Episodes of Low Back Pain during Deployment to Afghanistan. Spine (Phila Pa 1976). 2013. | Outcome criteria not fulfilled. |
| 149 | Rugulies R, Krause N. Effort-reward imbalance and incidence of low back and neck injuries in San Francisco transit operators. Occup Environ Med. 2008;65(8):525-33. | Exposure criteria not fulfilled. |
| 150 | Rugulies R, Krause N. Job strain, iso-strain, and the incidence of low back and neck injuries. A 7.5-year prospective study of San Francisco transit operators. Soc Sci Med 2005;61:27-39. | Outcome criteria not fulfilled. |
| 151 | Seidler A, Bergmann A, Jager M, Ellegast R, Ditchen D, Elsner G et al. Cumulative occupational lumbar load and lumbar disc disease--results of a German multi-center case-control study (EPILIFT). BMC Musculoskelet Disord 2009 Vol. 10 Pages 48 | Exposure criteria not fulfilled. |
| 152 | Seidler A, Euler U, Bolm-Audorff U, Ellegast R, Grifka J, Haerting J et al. Physical workload and accelerated occurrence of lumbar spine diseases: risk and rate advancement periods in a German multicenter case-control study. Scand J Work Environ Health 2011 Vol. 37 Issue 1 Pages 30-6 | Exposure criteria not fulfilled. |
| 153 | Shannon HS, Woodward CA, Cunningham CE, McIntosh J, Lendrum B, Brown J, et al. Changes in general health and musculoskeletal outcomes in the workforce of a hospital undergoing rapid change: a longitudinal study. J Occup Health Psychol 2001;6:3-14. | Outcome criteria not fulfilled. |
| 154 | Shaw WS, Pransky G, Winters T. The Back Disability Risk Questionnaire for work-related, acute back pain: prediction of unresolved problems at 3-month follow-up. J Occup Environ Med 2009;51:185-94. | Outcome criteria not fulfilled. |
| 155 | Smedley J, Egger P, Cooper C, Coggon D. Prospective cohort study of predictors of incident low back pain in nurses. BMJ 1997;314:1225-8. | Outcome criteria not fulfilled. |

| | | |
|---|---|---|
| 156 | Sterud T, Tynes T. Work-related psychosocial and mechanical risk factors for low back pain: a 3-year follow-up study of the general working population in Norway. Occup Environ Med 2013;70:296-302. | Outcome criteria not fulfilled. |
| 157 | Stevenson JM, Weber CL, Smith JT, Dumas GA, Albert WJ. A longitudinal study of the development of low back pain in an industrial population. Spine (Phila Pa 1976). 2001;26(12):1370-7. | Exposure criteria not fulfilled. |
| 158 | Stobbe TJ, Plummer RW, Jensen RC, Attfield MD. Incidence of low back injuries among nursing personnel as a function of patient lifting frequency. Journal of Safety Research 1988;19:21-8. | Study design criteria not fulfilled. |
| 159 | Stomp-van den Berg SG, Hendriksen IJ, Bruinvels DJ, Twisk JW, van Mechelen W, van Poppel MN. Predictors for post-partum pelvic girdle pain in working women: the Mom@Work cohort study. Pain 2012;153:2370-9. | Outcome criteria not fulfilled. |
| 160 | Studnek JR, Crawford JM. Factors associated with back problems among emergency medical technicians. Am J Ind Med 2007;50:464-9. | Outcome criteria not fulfilled. |
| 161 | Thorbjornsson CB, Alfredsson L, Fredriksson K, Michelsen H, Punnett L, Vingard E, et al. Physical and psycho-social factors related to low back pain during a 24-year period. A nested case-control analysis. Spine (Phila Pa 1976) 2000;25:369-74; discussion 375. | Outcome criteria not fulfilled. |
| 162 | Tiemessen IJ, Hulshof CT, Frings-Dresen MH. Low back pain in drivers exposed to whole body vibration: ana-lysis of a dose-response pattern. Occup Environ Med 2008;65:667-75. | Outcome criteria not fulfilled. |
| 163 | Torp S, Riise T, Moen BE. The impact of psychosocial work factors on musculo-skeletal pain: a prospective study. J Occup Environ Med 2001;43:120-6. | Outcome criteria not fulfilled. |
| 164 | Trinkoff AM, Le R, Geiger-Brown J, Lipscomb J, Lang G. Longitudinal relationship of work hours, mandatory overtime, and on-call to musculoskeletal problems in nurses. Am J Ind Med 2006;49:964-71. | Outcome criteria not fulfilled. |
| 165 | Tubach F, Leclerc A, Landre MF, Pietri- Taleb F. Risk factors for sick leave due to low back pain: a prospective study. J Occup Environ Med 2002;44:451-8. | Outcome criteria not fulfilled. |
| 166 | van den Heuvel SG, Ariens GA, Boshuizen HC, Hoogendoorn WE, Bongers PM. Prognostic factors related to recurrent low-back pain and sickness absence. Scand J Work Environ Health 2004;30:459-67. | Outcome criteria not fulfilled. |
| 167 | Van Nieuwenhuyse A, Somville PR, Crombez G, Burdorf A, Verbeke G, Johannik K, et al. The role of physical workload and pain related fear in the development of low back pain in young workers: evidence from the BelCoBack Study; results after one year of follow up. Occup Environ Med 2006;63:45-52. | Outcome criteria not fulfilled. |
| 168 | van Poppel MN, Koes BW, Deville W, Smid T, Bouter LM. Risk factors for back pain incidence in industry: a pro-spective study. Pain 1998;77:81-6. | Outcome criteria not fulfilled. |
| 169 | Vandergrift JL, Gold JE, Hanlon A, Punnett L. Physical and psychosocial ergonomic risk factors for low back pain in automobile manufacturing workers. Occup Environ Med 2012; 69:29-34. Epub 2011 May 17. | Outcome criteria not fulfilled. |
| 170 | Ward MM, Reveille JD, Learch TJ, Davis JC, Jr., Weisman MH. Occupational physical activities and long-term functional and radiographic outcomes in patients with ankylosing spondylitis. Arthritis Rheum 2008;59:822-32. | Outcome criteria not fulfilled. |
| 171 | Venning PJ, Walter SD, Stitt LW. Personal and job-related factors as determinants of incidence of back injuries among nursing personnel. J Occup Med 1987;29:820-5. | Outcome criteria not fulfilled. |
| 172 | Verbeek JH, van der Beek AJ. Psychosocial factors at work and back pain: a prospective study in office workers. Int J Occup Med Environ Health 1999;12:29-39. | Outcome criteria not fulfilled. |
| 173 | Wergeland EL, Veiersted B, Ingre M, Olsson B, Akerstedt T, Bjornskau T, et al. A shorter workday as a means of reducing the occurrence of musculo-skeletal disorders. Scand J Work Environ Health 2003;29:27-34. | Outcome criteria not fulfilled. |
| 174 | Wickstrom GJ, Pentti J. Occupational factors affecting sick leave attributed to low-back pain. Scand J Work Environ Health 1998;24:145-52. | Outcome criteria not fulfilled. |
| 175 | Videman T, Battie MC, Parent E, Gibbons LE, Vainio P, Kaprio J. Progression and determinants of quantitative magnetic resonance imaging measures of lumbar disc degeneration: a five-year follow-up of adult male monozygotic twins. Spine (Phila Pa 1976) 2008;33:1484-90. | Outcome criteria not fulfilled. |
| 176 | Videman T, Ojajarvi A, Riihimaki H, Troup JD. Low back pain among nurses: a follow-up beginning at entry to the nursing school. Spine (Phila Pa 1976) 2005;30:2334-41. | Outcome criteria not fulfilled. |
| 177 | Wiktorin C, Vingard E, Mortimer M, Pernold G, Wigaeus-Hjelm E, Kilbom A, et al. Interview versus questionnaire for assessing physical loads in the population-based MUSIC-Norrtalje Study. Am J Ind Med 1999;35:441-55. | Outcome criteria not fulfilled. |
| 178 | Williams RA, Pruitt SD, Doctor JN, Epping-Jordan JE, Wahlgren DR, Grant I, et al. The contribution of job satisfaction to the transition from acute to chronic low back pain. Arch Phys Med Rehabil 1998;79:366-74. | Outcome criteria not fulfilled. |
| 179 | Vingard E, Alfredsson L, Hagberg M, Kilbom A, Theorell T, Waldenstrom M, et al. To what extent do current and past physical and psychosocial occupational factors explain care-seeking for low back pain in a working population? Results from the Musculo-skeletal Intervention Center-Norrtalje Study. Spine (Phila Pa 1976) 2000;25:493-500. | Outcome criteria not fulfilled. |
| 180 | Yang LQ, Spector PE, Chang CH, Gallant-Roman M, Powell J. Psychosocial precursors and physical consequences of workplace violence towards nurses: a longitudinal examination with naturally occurring groups in hospital settings. Int J Nurs Stud 2012;49:1091-102. | Outcome criteria not fulfilled. |
| 181 | Yassi A, Khokhar J, Tate R, Cooper J, Snow C, Vallentyne S. The epidemiology of back injuries in nurses at a large Canadian tertiary care hospital: implications for prevention. Occup Med (Lond). 1995;45(4):215-20. | Outcome criteria not fulfilled. |
| 182 | Zhao I, Bogossian F, Turner C. The effects of shift work and interaction between shift work and overweight/obesity on low back pain in nurses: results from a longitudinal study. J Occup Environ Med 2012;54:820-5. | Outcome criteria not fulfilled. |

| | | |
|---|---|---|
| 183 | Zochling J, Bohl-Buhler MH, Baraliakos X, Feldtkeller E, Braun J. Infection and work stress are potential triggers of ankylosing spondylitis. Clin Rheumatol 2006;25:660-6. | Outcome criteria not fulfilled. |
| 184 | Zwerling C, Ryan J, Schootman M. A case-control study of risk factors for industrial low back injury. The utility of preplacement screening in defining high-risk groups. Spine (Phila Pa 1976). 1993;18(9):1242-7. | Outcome criteria not fulfilled. |

**Table S9.** Excluded articles based on full-text read and reason for exclusion.

| **Reference: studies published after January 10, 2014** | | **Reason for exclusion** |
|---|---|---|
| 1 | M. Abid, H. U. Khan, M. H. Abid, A. Ijaz, M. Ahmad, M. T. Naeem, (2021). "Association of occupational risk factors with the level of lumbar disc nucleus pulposus herniation." Pakistan Journal of Medical and Health Sciences **15**(10): 2863-2864. | Study design criteria not fulfilled. |
| 2 | Ahlholm, V. H., Ronkko, V., Ala-Mursula, L., Karppinen, J., & Oura, P. (2021). Modeling the Multidimensional Predictors of Multisite Musculoskeletal Pain Across Adulthood-A Generalized Estimating Equations Approach. *Front Public Health*, *9*, 709778. | Outcome criteria not fulfilled. |
| 3 | Alghadir, A., Zafar, H., & Iqbal, Z. A. (2015). Work-related musculoskeletal disorders among dental professionals in Saudi Arabia. *J Phys Ther Sci*, *27*(4), 1107-1112. | Study design criteria not fulfilled. |
| 4 | Alghadir, A., Zafar, H., Iqbal, Z. A., & Al-Eisa, E. (2017). Work-Related Low Back Pain Among Physical Therapists in Riyadh, Saudi Arabia. *Workplace Health Saf*, *65*(8), 337-345. | Study design criteria not fulfilled. |
| 5 | Alhalabi MS, Alhaleeb H, Madani S. Risk factors associated with chronic low back pain in Syria. Avicenna J Med 2015 Vol. 5 Issue 4 Pages 110-6 | Exposure criteria not fulfilled. |
| 6 | Alrowayeh, H. N., Alnaser, M. Z., Alshatti, T. A., Saeed, R. S. (2021). Prevalence and Risk Factors of Work-Related Lower Back Pain among Radiographers in the State of Kuwait. Radiol Res Pract 2021 Vol. 2021 Pages 5365260. | Study design criteria not fulfilled. |
| 7 | Alziyadi, R. H., Elgezery, M. H., & Alziyadi, R. H. (2021). Prevalence of Low Back Pain and Its Associated Risk Factors among Female Nurses Working in a tertiary hospital in Dhahran, Eastern Province, Saudi Arabia. *World Family Medicine*, *19*(1), 173-182. | Study design criteria not fulfilled. |
| 8 | Amin, R., Safdar, B., & Masood, M. H. (2019). PSYCHOSOCIAL DETERMINANTS OF BACKACHE IN FEMALES. *Indo American Journal of Pharmaceutical Sciences*, *6*(6), 12915-12921. | Outcome criteria not fulfilled. |
| 9 | Andersen, L. L., Vinstrup, J., Sundstrup, E., Skovlund, S. V., Villadsen, E., & Thorsen, S. V. (2021). Combined ergonomic exposures and development of musculoskeletal pain in the general working population: A prospective cohort study. *Scand J Work Environ Health*, *47*(4), 287-295. | Outcome criteria not fulfilled. |
| 10 | Andersen, L. L., Vinstrup, J., Villadsen, E., Jay, K., & Jakobsen, M. D. (2019). Physical and Psychosocial Work Environmental Risk Factors for Back Injury among Healthcare Workers: Prospective Cohort Study. *Int J Environ Res Public Health*, *16*(22). | Outcome criteria not fulfilled. |
| 11 | Arcury, T. A., Chen, H., Mora, D. C., Walker, F. O., Cartwright, M. S., & Quandt, S. A. (2016). The effects of work organization on the health of immigrant manual workers: A longitudinal analysis. *Arch Environ Occup Health*, *71*(2), 66-73. | Outcome criteria not fulfilled. |
| 12 | Arvidsson, I., Gremark Simonsen, J., Lindegard-Andersson, A., Bjork, J., & Nordander, C. (2020). The impact of occupational and personal factors on musculoskeletal pain - a cohort study of female nurses, sonographers and teachers. *BMC Musculoskelet Disord*, *21*(1), 621. | Outcome criteria not fulfilled. |
| 13 | Assadi, S. (2022). "Carrying load and related health disorders and disability." Indian Journal of Occupational and Environmental Medicine **26**(2): 129-132. | Study design criteria not fulfilled. |
| 14 | Badarin, K., Hemmingsson, T., Hillert, L., & Kjellberg, K. (2021). Physical workload and increased frequency of musculoskeletal pain: a cohort study of employed men and women with baseline occasional pain. *Occup Environ Med*, *78*(8), 558-566. | Outcome criteria not fulfilled. |
| 15 | Bazazan, A., Dianat, I., Bahrampour, S., Talebian, A., Zandi, H., Sharafkhaneh, A., & Maleki-Ghahfarokhi, A. (2019). Association of musculoskeletal disorders and workload with work schedule and job satisfaction among emergency nurses. *Int Emerg Nurs*, *44*, 8-13. | Study design criteria not fulfilled. |
| 16 | Bergmann A, Bolm-Audorff U, Ditchen D, Ellegast R, Grifka J, Haerting J et al. Do Occupational Risks for Low Back Pain Differ From Risks for Specific Lumbar Disc Diseases?: Results of the German Lumbar Spine Study (EPILIFT). Spine (Phila Pa 1976) 2017 Vol. 42 Issue 20 Pages E1204-E1211. | Exposure criteria not fulfilled. |
| 17 | Bontrup, C., Taylor, W. R., Fliesser, M., Visscher, R., Green, T., Wippert, P. M., & Zemp, R. (2019). Low back pain and its relationship with sitting behaviour among sedentary office workers. *Appl Ergon*, *81*, 102894. | Study design criteria not fulfilled. |
| 18 | Bonzini, M., Bertù, L., Conti, M., D'Amato, A., Veronesi, G., Coggon, D. N., & Ferrario, M. M. (2014). 0168 Somatising tendency, occupational strain and musculoskeletal symptoms: results from a longitudinal study among Italian nurses. *Occupational and Environmental Medicine*, *71*(Suppl 1), A21.22-A21. | Other reasons (e.g., abstract, books). |
| 19 | Bonzini, M., Bertu, L., Veronesi, G., Conti, M., Coggon, D., & Ferrario, M. M. (2015). Is musculoskeletal pain a consequence or a cause of occupational stress? A longitudinal study. *Int Arch Occup Environ Health*, *88*(5), 607-612. | Outcome criteria not fulfilled. |
| 20 | A. Boutellier, C. Nuesch, P. Suter, G. Perrot and A. Mundermann (2022). Trunk muscle function and its association with functional limitations in sedentary occupation workers with and without chronic nonspecific low back pain. Journal of Back and Musculoskeletal Rehabilitation 2022 Vol. 35 Issue 4 Pages 783-791 | Outcome criteria not fulfilled. |

| | | |
|---|---|---|
| 21 | Bovenzi, M., & Schust, M. (2021). A prospective cohort study of low-back outcomes and alternative measures of cumulative external and internal vibration load on the lumbar spine of professional drivers. *Scand J Work Environ Health*, *47*(4), 277-286. | Outcome criteria not fulfilled. |
| 22 | Bovenzi, M., Schust, M., Menzel, G., Hofmann, J., & Hinz, B. (2015). A cohort study of sciatic pain and measures of internal spinal load in professional drivers. *Ergonomics*, *58*(7), 1088-1102. | Outcome criteria not fulfilled. |
| 23 | Bovenzi, M., Schust, M., Menzel, G., Prodi, A., & Mauro, M. (2015). Relationships of low back outcomes to internal spinal load: a prospective cohort study of professional drivers. *Int Arch Occup Environ Health*, *88*(4), 487-499. | Outcome criteria not fulfilled. |
| 24 | Brauer, C., Mikkelsen, S., Pedersen, E. B., Moller, K. L., Simonsen, E. B., Koblauch, H., Alkjaer, T., Helweg-Larsen, K., & Thygesen, L. C. (2020). Occupational lifting predicts hospital admission due to low back pain in a cohort of airport baggage handlers. *Int Arch Occup Environ Health*, *93*(1), 111-122. | Exposure criteria not fulfilled. |
| 25 | Bugajska, J., Zolnierczyk-Zreda, D., Jedryka-Goral, A., Gasik, R., Hildt-Ciupinska, K., Malinska, M., & Bedynska, S. (2013). Psychological factors at work and musculoskeletal disorders: a one year prospective study. *Rheumatol Int*, *33*(12), 2975-2983. | Outcome criteria not fulfilled. |
| 26 | Caputo E.L., M. R. Domingues, A. D. Bertoldi, P. H. Ferreira, M. L. Ferreira, D. Shirley, et al. (2021). Are leisure-time and work-related activities associated with low back pain during pregnancy? BMC Musculoskeletal Disorders 2021 Vol. 22 Issue 1 Pages 1-8. | Outcome criteria not fulfilled. |
| 27 | Chaiklieng, S., & Suggaravetsiri, P. (2020). Low Back Pain (LBP) Incidence, Ergonomics Risk and Workers' Characteristics in Relations to LBP in Electronics Assembly Manufacturing. *Indian J Occup Environ Med*, *24*(3), 183-187. | Outcome criteria not fulfilled. |
| 28 | Chakrabarty, S., Sarkar, K., Dev, S., Das, T., Mitra, K., Sahu, S., & Gangopadhyay, S. (2016). Impact of rest breaks on musculoskeletal discomfort of Chikan embroiderers of West Bengal, India: a follow up field study. *J Occup Health*, *58*(4), 365-372. | Outcome criteria not fulfilled. |
| 29 | Chan, E. W. M., Hamid, M. S. A., Din, F. H. M., Ahmad, R., Nadzalan, A. M., & Hafiz, E. (2019). Prevalence and factors associated with low back pain among Malaysian army personnel stationed in Klang Valley. *Biomedical Human Kinetics*, *11*(1), 9-18. | Study design criteria not fulfilled. |
| 30 | Chand, R. K., Roomi, M. A., Begum, S., & Mudassar, A. (2020). Prevalence of musculoskeletal disorders, associated risk factors and coping strategies among secondary school teachers in fiji. *Rawal Medical Journal*, *45*(2), 377-381. | Study design criteria not fulfilled. |
| 31 | Chang, K. C., Lee, H. C., Yen, C. L., Liao, Y. H., Hung, J. W., & Wu, C. Y. (2021). Low back pain-associated factors in female hospital-based personal care attendants. *Work*, *69*(1), 315-322. | Study design criteria not fulfilled. |
| 32 | Chaudhary, D. K., Palei, S. K., Kumar, V., Karmakar, N. C. (2022). Whole-body vibration exposure of heavy earthmoving machinery operators in surface coal mines: a comparative assessment of transport and non-transport earthmoving equipment operators. Int J Occup Saf Ergon 2022 Vol. 28 Issue 1 Pages 174-183. | Study design criteria not fulfilled. |
| 33 | Choochouy N, Saita S, Sirithian D. Prevalence of and factors associated with occupational health problems among hill farmers in Thailand. Southeast Asian J Trop Med Public Health. 2022;53(4):368-86. | Study design criteria not fulfilled. |
| 34 | Christensen, J. O., Johansen, S., & Knardahl, S. (2017). Psychological predictors of change in the number of musculoskeletal pain sites among Norwegian employees: a prospective study. *BMC Musculoskelet Disord*, *18*(1), 140. | Outcome criteria not fulfilled. |
| 35 | Christensen, J. O., Nielsen, M. B., Finne, L. B., & Knardahl, S. (2018). Comprehensive profiles of psychological and social work factors as predictors of site-specific and multi-site pain. *Scand J Work Environ Health*, *44*(3), 291-302. | Outcome criteria not fulfilled. |
| 36 | Clausen, T., Andersen, L. L., Holtermann, A., Jorgensen, A. F., Aust, B., & Rugulies, R. (2013). Do self-reported psychosocial working conditions predict low back pain after adjustment for both physical work load and depressive symptoms? A prospective study among female eldercare workers. *Occup Environ Med*, *70*(8), 538-544. | Outcome criteria not fulfilled. |
| 37 | Clays, E., Ketels, M., & Oakman, J. (2021). Low back and neck-shoulder pain: What is the role of objective and subjective measures in determining physical and psychosocial workplace hazards in non-sedentary jobs? *International Journal of Behavioral Medicine*, *28*(SUPPL 1), S134-S135. | Study design criteria not fulfilled. |
| 38 | Coenen, P., Douwes, M., van den Heuvel, S., & Bosch, T. (2016). Towards exposure limits for working postures and musculoskeletal symptoms - a prospective cohort study. *Ergonomics*, *59*(9), 1182-1192. | Outcome criteria not fulfilled. |
| 39 | Coenen, P., Kingma, I., Boot, C. R., Bongers, P. M., & van Dieen, J. H. (2014). Cumulative mechanical low-back load at work is a determinant of low-back pain. *Occup Environ Med*, *71*(5), 332-337. | Outcome criteria not fulfilled. |
| 40 | Coenen, P., Kingma, I., Boot, C. R., Twisk, J. W., Bongers, P. M., & van Dieen, J. H. (2013). Cumulative low back load at work as a risk factor of low back pain: a prospective cohort study. *J Occup Rehabil*, *23*(1), 11-18. | Other reasons (e.g., abstract, books). |
| 41 | Coenen, P., Mathiassen, S. E., Kingma, I., Boot, C. R., Bongers, P. M., & van Dieen, J. H. (2015). The effect of the presence and characteristics of an outlying group on exposure-outcome associations. *Scand J Work Environ Health*, *41*(1), 65-74. | Outcome criteria not fulfilled. |
| 42 | Coggon, D., Ntani, G., Palmer, K. T., Felli, V. E., Harari, F., Quintana, L. A., Felknor, S. A., Rojas, M., Cattrell, A., Vargas-Prada, S., Bonzini, M., Solidaki, E., Merisalu, E., Habib, R. R., Sadeghian, F., Kadir, M. M., Warnakulasuriya, S. S. P., Matsudaira, K., Nyantumbu-Mkhize, B., Harcombe, H. (2019). Drivers of international variation in prevalence of disabling low back pain: Findings from the Cultural and Psychosocial Influences on Disability study. *Eur J Pain*, *23*(1), 35-45. | Study design criteria not fulfilled. |
| 43 | Damrongsak, M., Prapanjaroensin, A., & Brown, K. C. (2018). Predictors of Back Pain in Firefighters. *Workplace Health Saf*, *66*(2), 61-69. | Study design criteria not fulfilled. |
| 44 | Das, B., & Gangopadhyay, S. (2015). Prevalence of musculoskeletal disorders and physiological stress among adult, male potato cultivators of West Bengal, India. *Asia Pac J Public Health*, *27*(2), NP1669-1682. | Study design criteria not fulfilled. |

| | | |
|---|---|---|
| 45 | Das, D., Kumar, A., & Sharma, M. (2021). Risk factors associated with musculoskeletal disorders among gemstone polishers in Jaipur, India. *Int J Occup Saf Ergon*, *27*(1), 95-105. | Study design criteria not fulfilled. |
| 46 | Das B. Ergonomic and psychosocial risk factors for low back pain among rice farmers in West Bengal, India. Work. 2022;72(3):967-77. | Study design criteria not fulfilled. |
| 47 | de Alwis, M. P., & Garme, K. (2020). Effect of occupational exposure to shock and vibration on health in high-performance marine craft occupants. *Proceedings of the Institution of Mechanical Engineers, Part M: Journal of Engineering for the Maritime Environment*, *235*(2), 394-409. | Other reasons (e.g., abstract, books). |
| 48 | de Alwis, M. P., LoMartire, R., Äng, B. O., & Garme, K. (2020). Exposure aboard high-performance marine craft increases musculoskeletal pain and lowers contemporary work capacity of the occupants. *Proceedings of the Institution of Mechanical Engineers, Part M: Journal of Engineering for the Maritime Environment*, *235*(3), 750-762. | Other reasons (e.g., abstract, books). |
| 49 | Dengler K, Hiesinger K, Tisch A. Digital transformation: The role of computer use in employee health. Econ Hum Biol. 2022;46:101137. | Outcome criteria not fulfilled. |
| 50 | Dick, R. B., Lowe, B. D., Lu, M. L., & Krieg, E. F. (2020). Trends in Work-Related Musculoskeletal Disorders From the 2002 to 2014 General Social Survey, Quality of Work Life Supplement. *J Occup Environ Med*, *62*(8), 595-610. | Study design criteria not fulfilled. |
| 51 | Dragioti, E., Gerdle, B., & Larsson, B. (2019). Longitudinal Associations between Anatomical Regions of Pain and Work Conditions: A Study from The SwePain Cohort. *Int J Environ Res Public Health*, *16*(12), 16. | Outcome criteria not fulfilled. |
| 52 | Duenas, M., Moral-Munoz, J. A., Palomo-Osuna, J., Salazar, A., De Sola, H., & Failde, I. (2020). Differences in physical and psychological health in patients with chronic low back pain: a national survey in general Spanish population. *Qual Life Res*, *29*(11), 2935-2947. | Other reasons (e.g., abstract, books). |
| 53 | Ekblom-Bak, E., Stenling, A., Salier Eriksson, J., Hemmingsson, E., Kallings, L. V., Andersson, G., Wallin, P., Ekblom, O., Ekblom, B., & Lindwall, M. (2020). Latent profile analysis patterns of exercise, sitting and fitness in adults - Associations with metabolic risk factors, perceived health, and perceived symptoms. *PLoS One*, *15*(4), e0232210. | Study design criteria not fulfilled. |
| 54 | Ekholm O, Diasso PDK, Davidsen M, Kurita GP, Sjogren P. Increasing prevalence of chronic non-cancer pain in Denmark from 2000 to 2017: A population-based survey. Eur J Pain. 2022;26(3):624-33. | Outcome criteria not fulfilled. |
| 55 | Elvis C, France N, Patience E. Risk factors for work-related musculoskeletal disorders among welders in the informal sector under resource constrained settings. Work. 2022;72(1):239-52. | Study design criteria not fulfilled. |
| 56 | Ervasti, J., Pietilainen, O., Rahkonen, O., Lahelma, E., Kouvonen, A., Lallukka, T., & Manty, M. (2019). Joint contribution of rotation of the back and repetitive movements to disability pension using job exposure matrix data. *Eur J Public Health*, *29*(6), 1079-1084. | Outcome criteria not fulfilled. |
| 57 | Essien, S. K., Bath, B., Koehncke, N., Trask, C., & Saskatchewan Farm Injury Cohort Study, T. (2016). Association Between Farm Machinery Operation and Low Back Disorder in Farmers: A Retrospective Cohort Study. *J Occup Environ Med*, *58*(6), e212-217. | Outcome criteria not fulfilled. |
| 58 | Estrada-Munoz C, Madrid-Casaca H, Salazar-Sepulveda G, Contreras-Barraza N, Iturra-Gonzalez J, Vega-Munoz A. Musculoskeletal Symptoms and Assessment of Ergonomic Risk Factors on a Coffee Farm. Appl Sci-Basel. 2022;12(15):17. | Outcome criteria not fulfilled. |
| 59 | Euro, U., Knekt, P., Rissanen, H., Aromaa, A., Karppinen, J., & Heliovaara, M. (2018). Risk factors for sciatica leading to hospitalization. *Eur Spine J*, *27*(7), 1501-1508. | Exposure criteria not fulfilled. |
| 60 | Euro U, Heliovaara M, Shiri R, Knekt P, Rissanen H et al. Work-related risk factors for sciatica leading to hospitalization. Sci Rep 2019 Vol. 9 Issue 1 Pages 6562. | Exposure criteria not fulfilled. |
| 61 | Farioli, A., Mattioli, S., Quaglieri, A., Curti, S., Violante, F. S., Coggon, D., & Andersen, B. (2014). Musculoskeletal pain in Europe: the role of personal, occupational, and social risk factors. *Scand J Work Environ Health*, *40*(1), 36-46. | Study design criteria not fulfilled. |
| 62 | Feijo FR, Pearce N, Faria NMX, Carvalho MP, Szortyka ALSC, Oliveira PAB, et al. The Role of Workplace Bullying in Low Back Pain: A Study With Civil Servants From a Middle-Income Country. J Pain. 2022;23(3):459-71. | Study design criteria not fulfilled. |
| 63 | Fliesser, M., De Witt Huberts, J., & Wippert, P. M. (2018). Education, job position, income or multidimensional indices? Associations between different socioeconomic status indicators and chronic low back pain in a German sample: a longitudinal field study. *BMJ Open*, *8*(4), e020207. | Exposure criteria not fulfilled. |
| 64 | Friel, C. P., Pascual, C. B., Duran, A. T., Goldsmith, J., & Diaz, K. M. (2020). Joint associations of occupational standing and occupational exertion with musculoskeletal symptoms in a US national sample. *Occup Environ Med.* | Study design criteria not fulfilled. |
| 65 | Gaowgzeh, R. A. M. (2019). Low back pain among nursing professionals in Jeddah, Saudi Arabia: Prevalence and risk factors. *J Back Musculoskelet Rehabil*, *32*(4), 555-560. | Study design criteria not fulfilled. |
| 66 | Garg, A., Boda, S., Hegmann, K. T., Moore, J. S., Kapellusch, J. M., Bhoyar, P., Thiese, M. S., Merryweather, A., Deckow-Schaefer, G., Bloswick, D., & Malloy, E. J. (2014). The NIOSH lifting equation and low-back pain, Part 1: Association with low-back pain in the backworks prospective cohort study. *Hum Factors*, *56*(1), 6-28. | Outcome criteria not fulfilled. |
| 67 | Garg, A., Kapellusch, J. M., Hegmann, K. T., Moore, J. S., Boda, S., Bhoyar, P., Thiese, M. S., Merryweather, A., Deckow-Schaefer, G., Bloswick, D., & Malloy, E. J. (2014). The NIOSH lifting equation and low-back pain, Part 2: Association with seeking care in the backworks prospective cohort study. *Hum Factors*, *56*(1), 44-57. | Outcome criteria not fulfilled. |
| 68 | Glambek, M., Nielsen, M. B., Gjerstad, J., Einarsen, S., & Croft. (2018). Gender differences in the relationship between workplace bullying and subjective back and neck pain: A two-wave study in a Norwegian probability sample. *J Psychosom Res*, *106*, 73-75. | Outcome criteria not fulfilled. |

| | | |
|---|---|---|
| 69 | Gunal, A., & Demirturk, F. (2021). Occupational hazards, sleep quality and musculoskeletal problems of pregnant workers. *J Obstet Gynaecol*, 1-5. | Study design criteria not fulfilled. |
| 70 | Gustafsson, K., Marklund, S., Aronsson, G., & Leineweber, C. (2019). Physical work environment factors affecting risk for disability pension due to mental or musculoskeletal diagnoses among nursing professionals, care assistants and other occupations: a prospective, population-based cohort study. *BMJ Open*, *9*(10), e026491. | Outcome criteria not fulfilled. |
| 71 | Habib, M. M. (2015). Ergonomic risk factor identification for sewing machine operators through supervised occupational therapy fieldwork in Bangladesh: A case study. *Work*, *50*(3), 357-362. | Study design criteria not fulfilled. |
| 72 | Halonen, J. I., Shiri, R., Manty, M., Sumanen, H., Solovieva, S., Viikari-Juntura, E., Kahonen, M., Lehtimaki, T., Raitakari, O. T., & Lallukka, T. (2019). Exposure to heavy physical work from early to later adulthood and primary healthcare visits due to musculoskeletal diseases in midlife: a register linked study. *BMJ Open*, *9*(8), e031564. | Outcome criteria not fulfilled. |
| 73 | Halonen JI, Shiri R, Magnusson Hanson LL, Lallukka T. Risk and Prognostic Factors of Low Back Pain: Repeated Population-based Cohort Study in Sweden. Spine (Phila Pa 1976) 2019 Vol. 44 Issue 17 Pages 1248-1255 | Exposure criteria not fulfilled. |
| 74 | Hansen, B. B., Kirkeskov, L., Begtrup, L. M., Boesen, M., Bliddal, H., Christensen, R., Andreasen, D. L., Kristensen, L. E., Flachs, E. M., & Kryger, A. I. (2019). Early occupational intervention for people with low back pain in physically demanding jobs: A randomized clinical trial. *PLoS Med*, *16*(8), e1002898. | Outcome criteria not fulfilled. |
| 75 | Hassani M, Hesampour R, Bartnicka J, Monjezi N, Ezbarami SM. Evaluation of working conditions, work postures, musculoskeletal disorders and low back pain among sugar production workers. Work. 2022. | Outcome criteria not fulfilled. |
| 76 | Hemati, K., Darbandi, Z., Kabir-Mokamelkhah, E., Poursadeghiyan, M., Ghasemi, M. S., Mohseni-Ezhiye, M., Abdolahian, Y., Aghilinejad, M., Ali Salehi, M., & Dehghan, N. (2020). Ergonomic intervention to reduce musculoskeletal disorders among flour factory workers. *Work*, *67*(3), 611-618. | Other reasons (e.g., abstract, books). |
| 77 | Henriques, M., Sacadura-Leite, E. M., & Serranheira, F. (2019). Low back pain among hospital nursing assistants. *Rev Bras Med Trab*, *17*(3), 370-377. | Study design criteria not fulfilled. |
| 78 | Heuch I, Hecuh I, Hagen K, Zwart A. Physical activity level at work and risk of chronic low back pain: A follow-up in the Nord-Trondelag Health Study. PLoS One 2017 Vol. 12 Issue 4 Pages e0175086. | Exposure criteria not fulfilled. |
| 79 | Hong, C., Lee, C. G., & Song, H. (2021). Characteristics of lumbar disc degeneration and risk factors for collapsed lumbar disc in Korean farmers and fishers. *Annals of Occupational and Environmental Medicine*, *33*(1), e16. | Outcome criteria not fulfilled. |
| 80 | Hung, Y. J., Shih, T. T., Chen, B. B., Hwang, Y. H., Ma, L. P., Huang, W. C., Liou, S. H., Ho, I. K., & Guo, Y. L. (2014). The dose-response relationship between cumulative lifting load and lumbar disk degeneration based on magnetic resonance imaging findings. *Phys Ther*, *94*(11), 1582-1593. | Study design criteria not fulfilled. |
| 81 | Hviid Andersen, J., Frost, P., Frølund Thomsen, J., Donbaek Jensen, L., & Wulff Svendsen, S. (2014). Back Surgery in relation to occupational lifting. A cohort study based on the Musculoskeletal Research Database at the Danish Ramazzini Centre. *Occupational and Environmental Medicine*, *71*(Suppl 1), A33.31-A33. | Other reasons (e.g., abstract, books). |
| 82 | Ishimoto, Y., C. Cooper, G. Ntani, H. Yamada, H. Hashizume, K. Nagata, et al. (2019). Is radiographic lumbar spondylolisthesis associated with occupational exposures? Findings from a nested case control study within the Wakayama spine study. BMC Musculoskelet Disord 2019 Vol. 20 Issue 1 Pages 618. | Outcome criteria not fulfilled. |
| 83 | Ishimoto, Y., C. Cooper, G. Ntani, H. Yamada, H. Hashizume, K. Nagata, et al. (2019). Factory and construction work is associated with an increased risk of severe lumbar spinal stenosis on MRI: A case control analysis within the wakayama spine study. Am J Ind Med 2019 Vol. 62 Issue 5 Pages 430-438. | Outcome criteria not fulfilled. |
| 84 | Jackson JA, Liv P, Sayed-Noor AS, Punnett L, Wahlstrom J. Risk factors for surgically treated cervical spondylosis in male construction workers: a 20-year prospective study. Spine Journal. 2022. | Outcome criteria not fulfilled. |
| 85 | Jo, H., Lim, O. B., Ahn, Y. S., Chang, S. J., & Koh, S. B. (2021). Negative Impacts of Prolonged Standing at Work on Musculoskeletal Symptoms and Physical Fatigue: The Fifth Korean Working Conditions Survey. *Yonsei Med J*, *62*(6), 510-519. | Study design criteria not fulfilled. |
| 86 | Jorgensen, M. B., Nabe-Nielsen, K., Clausen, T., & Holtermann, A. (2013). Independent effect of physical workload and childhood socioeconomic status on low back pain among health care workers in Denmark. *Spine (Phila Pa 1976)*, *38*(6), E359-366. | Outcome criteria not fulfilled. |
| 87 | Kapellusch, J. M., Garg, A., Boda, S., Hegmann, K. T., Moore, J. S., Thiese, M. S., Merryweather, A., Tomich, S., Foster, J. C., Bloswick, D., & Malloy, E. J. (2014). Association between lifting and use of medication for low back pain: results from the Backworks Prospective Cohort Study. *J Occup Environ Med*, *56*(8), 867-877. | Outcome criteria not fulfilled. |
| 88 | Kawaguchi, M., Matsudaira, K., Sawada, T., Koga, T., Ishizuka, A., Isomura, T., & Coggon, D. (2017). Assessment of potential risk factors for new onset disabling low back pain in Japanese workers: findings from the CUPID (cultural and psychosocial influences on disability) study. *BMC Musculoskelet Disord*, *18*(1), 334. | Outcome criteria not fulfilled. |
| 89 | Khansa, I., Khansa, L., Westvik, T. S., Ahmad, J., Lista, F., & Janis, J. E. (2018). Work-Related Musculoskeletal Injuries in Plastic Surgeons in the United States, Canada, and Norway. *Plast Reconstr Surg*, *141*(1), 165e-175e. | Study design criteria not fulfilled. |
| 90 | Kim HR. Associations Between Workplace Violence, Mental Health, and Physical Health among Korean Workers: The Fifth Korean Working Conditions Survey. Workplace Health & Safety. 2022;70(3):161-72. | Study design criteria not fulfilled. |
| 91 | Kim, Y. H., & Jung, M. H. (2016). Effect of occupational health nursing practice on musculoskeletal pains among hospital nursing staff in South Korea. *Int J Occup Saf Ergon*, *22*(2), 199-206. | Study design criteria not fulfilled. |
| 92 | Korshoj, M., Jorgensen, M. B., Hallman, D. M., Lagersted-Olsen, J., Holtermann, A., & Gupta, N. (2018). Prolonged sitting at work is associated with a favorable time course of low-back pain among blue-collar workers: a prospective study in the DPhacto cohort. *Scand J Work Environ Health*, *44*(5), 530-538. | Outcome criteria not fulfilled. |
| 93 | Kumar V, Palei SK, Karmakar NC, Chaudhary DK. Whole-Body Vibration Exposure vis-a-vis Musculoskeletal Health Risk of Dumper Operators Compared to a Control Group in Coal Mines. Saf Health Work. 2022;13(1):73-7. | Outcome criteria not fulfilled. |

| | | |
|---|---|---|
| 94 | Lagersted-Olsen, J., Thomsen, B. L., Holtermann, A., Sogaard, K., & Jorgensen, M. B. (2016). Does objectively measured daily duration of forward bending predict development and aggravation of low-back pain? A prospective study. *Scand J Work Environ Health*, *42*(6), 528-537. | Outcome criteria not fulfilled. |
| 95 | Lallukka, T., Viikari-Juntura, E., Viikari, J., Kahonen, M., Lehtimaki, T., Raitakari, O. T., & Solovieva, S. (2017). Early work-related physical exposures and low back pain in midlife: the Cardiovascular Risk in Young Finns Study. *Occup Environ Med*, *74*(3), 163-168. | Outcome criteria not fulfilled. |
| 96 | Larson RE, Johnson AW, Bruening D, Ridge ST, Mitchell UH. Low back pain and lumbar multifidus cross-sectional area, multifidus activation, and low back force in healthcare workers. Int J Ind Ergon. 2022;88:5. | Study design criteria not fulfilled. |
| 97 | Ling CF, Radin Umar RZ, Ahmad N. Development of a predictive model for work-relatedness of MSDs among semiconductor back-end workers. Int J Occup Saf Ergon. 2022;28(2):872-82. | Outcome criteria not fulfilled. |
| 98 | Liu, H.-C., Cheng, Y., & Ho, J.-J. (2020). Associations of ergonomic and psychosocial work hazards with musculoskeletal disorders of specific body parts: A study of general employees in Taiwan. *International Journal of Industrial Ergonomics*, *76*, 8. | Study design criteria not fulfilled. |
| 99 | Liu, L., Chen, S. G., Tang, S. C., Wang, S., He, L. H., Guo, Z. H., Li, J. Y., Yu, S. F., & Wang, Z. X. (2015). How Work Organization Affects the Prevalence of WMSDs: A Case-control Study. *Biomed Environ Sci*, *28*(9), 627-633. | Study design criteria not fulfilled. |
| 100 | Lovgren, M., Gustavsson, P., Melin, B., & Rudman, A. (2014). Neck/shoulder and back pain in new graduate nurses: A growth mixture modeling analysis. *Int J Nurs Stud*, *51*(4), 625-639. | Outcome criteria not fulfilled. |
| 101 | Lu, M. L., Waters, T. R., Krieg, E., & Werren, D. (2014). Efficacy of the revised NIOSH lifting equation to predict risk of low-back pain associated with manual lifting: a one-year prospective study. *Hum Factors*, *56*(1), 73-85. | Outcome criteria not fulfilled. |
| 102 | Lundberg, U. (2015). Work conditions and back pain problems. *Stress Health*, *31*(1), 1-4. | Other reasons (e.g., abstract, books). |
| 103 | Lunde, L. K., Koch, M., Knardahl, S., & Veiersted, K. B. (2017). Associations of objectively measured sitting and standing with low-back pain intensity: a 6-month follow-up of construction and healthcare workers. *Scand J Work Environ Health*, *43*(3), 269-278. | Outcome criteria not fulfilled. |
| 104 | Lunde, L. K., Koch, M., Merkus, S. L., Knardahl, S., Waersted, M., & Veiersted, K. B. (2019). Associations of objectively measured forward bending at work with low-back pain intensity: a 2-year follow-up of construction and healthcare workers. *Occup Environ Med*, *76*(9), 660-667. | Outcome criteria not fulfilled. |
| 105 | Luo, Y., Wang, J., Pei, J., Rong, Y., Liu, W., Tang, P., Cai, W., & Yin, G. (2020). Interactions between the MMP-3 gene rs591058 polymorphism and occupational risk factors contribute to the increased risk for lumbar disk herniation: A case-control study. *J Clin Lab Anal*, *34*(7), e23273. | Exposure criteria not fulfilled. |
| 106 | Magnusson Hanson, L. L., Madsen, I. E., Rugulies, R., Peristera, P., Westerlund, H., & Descatha, A. (2017). Temporal relationships between job strain and low-back pain. *Scand J Work Environ Health*, *43*(5), 396-404. | Other reasons (e.g., abstract, books). |
| 107 | Matsudaira, K., Kawaguchi, M., Isomura, T., Arisaka, M., Fujii, T., Takeshita, K., Kitagawa, T., Miyoshi, K., & Konishi, H. (2013). Identification of risk factors for new-onset sciatica in Japanese workers: findings from the Japan epidemiological research of Occupation-related Back pain study. *Spine (Phila Pa 1976)*, *38*(26), E1691-1700. | Other reasons (e.g., abstract, books). |
| 108 | Mierswa, T., & Kellmann, M. (2017). Psychological detachment as moderator between psychosocial work conditions and low back pain development. *Int J Occup Med Environ Health*, *30*(2), 313-327. | Outcome criteria not fulfilled. |
| 109 | Mierswa, T., Kellmann, M., & Alexopoulos. (2017). Differences in low back pain occurrence over a 6-month period between four recovery-stress groups. *Work*, *58*(2), 193-202. | Study design criteria not fulfilled. |
| 110 | Minghelli, B., Ettro, N., Simao, J., & Mauricio, K. (2019). Work-related self-reported musculoskeletal disorders in hypermarket cashiers: a study in south of Portugal. *Med Lav*, *110*(3), 191-201. | Study design criteria not fulfilled. |
| 111 | Mishra, S., & Sarkar, K. (2021). Work-related musculoskeletal disorders and associated risk factors among urban metropolitan hairdressers in India. *J Occup Health*, *63*(1), e12200. | Study design criteria not fulfilled. |
| 112 | Miwa, S., Yokogawa, A., Kobayashi, T., Nishimura, T., Igarashi, K., Inatani, H., & Tsuchiya, H. (2015). Risk factors of recurrent lumbar disk herniation: a single center study and review of the literature. *J Spinal Disord Tech*, *28*(5), E265-269. | Outcome criteria not fulfilled. |
| 113 | Monaco, M. G. L., Uccello, R., Muoio, M., Greco, A., Spada, S., Coggiola, M., Pedata, P., Caputo, F., Chiodini, P., Miraglia, N., & Aublet-Cuvelier. (2019). Work-related upper limb disorders and risk assessment among automobile manufacturing workers: A retrospective cohort analysis. *Work*, *64*(4), 755-761. | Outcome criteria not fulfilled. |
| 114 | Monnier, A., Djupsjobacka, M., Larsson, H., Norman, K., & Ang, B. O. (2016). Risk factors for back pain in marines; a prospective cohort study. *BMC Musculoskelet Disord*, *17*, 319. | Outcome criteria not fulfilled. |
| 115 | Monnier, A., Larsson, H., Nero, H., Djupsjobacka, M., & Ang, B. O. (2019). A longitudinal observational study of back pain incidence, risk factors and occupational physical activity in Swedish marine trainees. *BMJ Open*, *9*(5), e025150. | Exposure criteria not fulfilled. |
| 116 | Monteiro, L. F., dos Santos, J. W., de Alsina, O. L. S., Univ Fed Sergipe, S. C. S. B., Univ Tiradentes, I. T., & Res, A. S. B. (2020). Association Between Manual Handling of Loads and Occupational Low Back Pain: A Case-Control Study with Brazilians Workers. *16th International Symposium on Occupational Safety and Hygiene (SHO)*, *277*, 471-479. | Other reasons (e.g., abstract, books). |
| 117 | Moshe, S., Zack, O., Finestone, A. S., Mishal, M., Segal, N., Slodownik, D., & Yagev, Y. (2016). The incidence and worsening of newly diagnosed low back pain in a population of young male military recruits. *BMC Musculoskelet Disord*, *17*, 279. | Study design criteria not fulfilled. |

| | | |
|---|---|---|
| 118 | Mozafari, A., Vahedian, M., Mohebi, S., & Najafi, M. (2015). Work-related musculoskeletal disorders in truck drivers and official workers. *Acta Med Iran*, *53*(7), 432-438. | Study design criteria not fulfilled. |
| 119 | Muslim, K., & Nussbaum, M. A. (2015). Musculoskeletal symptoms associated with posterior load carriage: An assessment of manual material handling workers in Indonesia. *Work*, *51*(2), 205-213. | Study design criteria not fulfilled. |
| 120 | Muthukrishnan, R., & Maqbool Ahmad, J. (2020). Ergonomic risk factors and risk exposure level of nursing tasks: association with work-related musculoskeletal disorders in nurses. *European Journal of Physiotherapy*, *23*(4), 248-253. | Study design criteria not fulfilled. |
| 121 | Nambiema, A., Bertrais, S., Bodin, J., Fouquet, N., Aublet-Cuvelier, A., Evanoff, B., Descatha, A., & Roquelaure, Y. (2020). Proportion of upper extremity musculoskeletal disorders attributable to personal and occupational factors: results from the French Pays de la Loire study. *BMC Public Health*, *20*(1), 456. | Outcome criteria not fulfilled. |
| 122 | Ncube, F., Kanda, A., & Dhlakama, P. (2019). Postural risk associated with Wooden Steel Chairs and Stackable Arm Chairs in a low-income country. *Work*, *64*(3), 579-586. | Study design criteria not fulfilled. |
| 123 | Neupane, S., Nygard, C. H., Prakash, K. C., von Bonsdorff, M. B., von Bonsdorff, M. E., Seitsamo, J., Rantanen, T., Ilmarinen, J., & Leino-Arjas, P. (2018). Multisite musculoskeletal pain trajectories from midlife to old age: a 28-year follow-up of municipal employees. *Occup Environ Med*, *75*(12), 863-870. | Outcome criteria not fulfilled. |
| 124 | Ng, Y. M., Voo, P., & Maakip, I. (2019). Psychosocial factors, depression, and musculoskeletal disorders among teachers. *BMC Public Health*, *19*(1), 234. | Study design criteria not fulfilled. |
| 125 | Ngabirano, L., Fadel, M., Leclerc, A., Evanoff, B. A., Dale, A. M., Roquelaure, Y., & Descatha, A. (2020). Comparison Between a Job-Exposure Matrix (JEM) Score and Self-Reported Exposures for Carrying Heavy Loads Over the Working Lifetime in the CONSTANCES Cohort. *Ann Work Expo Health*, *64*(4), 455-460. | Study design criteria not fulfilled. |
| 126 | Nissen, L. R., Marott, J. L., Gyntelberg, F., & Guldager, B. (2014). Deployment-related risk factors of low back pain: a study among danish soldiers deployed to Iraq. *Mil Med*, *179*(4), 451-458. | Study design criteria not fulfilled. |
| 127 | Nordin, M., Bolin, M., & Allvin. (2014). Do Sex Differences in the Association between Work Exposure and Health in the Manufacturing Industry Depend on Work Context? Results from the WOLF-Study. *Psychology*, *05*(08), 896-907. | Outcome criteria not fulfilled. |
| 128 | Oakman, J., Neupane, S., & Nygard, C. H. (2016). Does age matter in predicting musculoskeletal disorder risk? An analysis of workplace predictors over 4 years. *Int Arch Occup Environ Health*, *89*(7), 1127-1136. | Outcome criteria not fulfilled. |
| 129 | Oakman J, Ketels M, Clays E. Low back and neck pain: objective and subjective measures of workplace psychosocial and physical hazards. Int Arch Occup Environ Health. 2021;94(7):1637-44. | Outcome criteria not fulfilled. |
| 130 | Oliveira, V. C., Ferreira, M. L., Refshauge, K. M., Maher, C. G., Griffin, A. R., Hopper, J. L., & Ferreira, P. H. (2015). Risk factors for low back pain: insights from a novel case-control twin study. *Spine J*, *15*(1), 50-57. | Outcome criteria not fulfilled. |
| 131 | Palfreyman, S. (2015). The effect of daily walking steps on preventing neck and low back pain in sedentary workers: A 1-year prospective cohort study. *International Journal of Osteopathic Medicine*, *18*(2), 155-156. | Outcome criteria not fulfilled. |
| 132 | Pandalai, S. P., Wheeler, M. W., & Lu, M. L. (2017). Non-chemical Risk Assessment for Lifting and Low Back Pain Based on Bayesian Threshold Models. *Saf Health Work*, *8*(2), 206-211. | Outcome criteria not fulfilled. |
| 133 | Park, J., Han, B. Y., & Kim, Y. (2017). Gender differences in occupations and complaints of musculoskeletal symptoms: Representative sample of South Korean workers. *Am J Ind Med*, *60*(4), 342-349. | Study design criteria not fulfilled. |
| 134 | Park, J., & Kim, Y. (2020). Factors Related to Physical and Mental Health in Workers With Different Categories of Employment. *J Occup Environ Med*, *62*(7), 511-518. | Study design criteria not fulfilled. |
| 135 | Picavet HS, Pas LW, Oostrom SH, Ploeg HP, Verschuren WM, Proper KI. The Relation between Occupational Sitting and Mental, Cardiometabolic, and Musculoskeletal Health over a Period of 15 Years--The Doetinchem Cohort Study. PLoS One 2016 Vol. 11 Issue 1 Pages e0146639. | Exposure criteria not fulfilled. |
| 136 | Piranveyseh, P., Motamedzade, M., Osatuke, K., Mohammadfam, I., Moghimbeigi, A., Soltanzadeh, A., & Mohammadi, H. (2016). Association between psychosocial, organizational and personal factors and prevalence of musculoskeletal disorders in office workers. *Int J Occup Saf Ergon*, *22*(2), 267-273. | Study design criteria not fulfilled. |
| 137 | Plouvier, S., Chastang, J. F., Cyr, D., Bonenfant, S., Descatha, A., Goldberg, M., & Leclerc, A. (2015). Occupational biomechanical exposure predicts low back pain in older age among men in the Gazel Cohort. *Int Arch Occup Environ Health*, *88*(4), 501-510. | Outcome criteria not fulfilled. |
| 138 | Prado-León LR. Push/Pull Risk Factors at Workstations: A Case-Control Study of Mexican Industrial Workers. Human Factors and Ergonomics in Manufacturing & Service Industries 2014;24(3):328-35. doi: https://doi.org/10.1002/hfm.20380 | Exposure criteria not fulfilled. |
| 139 | Prakash, K. C., Neupane, S., Leino-Arjas, P., von Bonsdorff, M. B., Rantanen, T., von Bonsdorff, M. E., Seitsamo, J., Ilmarinen, J., & Nygard, C. H. (2017). Work-Related Biomechanical Exposure and Job Strain as Separate and Joint Predictors of Musculoskeletal Diseases: A 28-Year Prospective Follow-up Study. *Am J Epidemiol*, *186*(11), 1256-1267. | Outcome criteria not fulfilled. |
| 140 | Puschmann, A. K., Driesslein, D., Beck, H., Arampatzis, A., Moreno Catala, M., Schiltenwolf, M., Mayer, F., & Wippert, P. M. (2020). Stress and Self-Efficacy as Long-Term Predictors for Chronic Low Back Pain: A Prospective Longitudinal Study. *J Pain Res*, *13*, 613-621. | Outcome criteria not fulfilled. |
| 141 | Ramond-Roquin, A., Bodin, J., Serazin, C., Parot-Schinkel, E., Ha, C., Richard, I., Petit Le Manach, A., Fouquet, N., & Roquelaure, Y. (2015). Biomechanical constraints remain major risk factors for low back pain. Results from a prospective cohort study in French male employees. *Spine J*, *15*(4), 559-569. | Outcome criteria not fulfilled. |

| | | |
|---|---|---|
| 142 | Rasmussen, C. D. N., Holtermann, A., & Jorgensen, M. B. (2018). Recall Bias in Low Back Pain Among Workers: Effects of Recall Period and Individual and Work-Related Factors. *Spine (Phila Pa 1976)*, *43*(12), E727-E733. | No measure of an association. |
| 143 | Rasmussen-Barr, E., Grooten, W. J. A., Hallqvist, J., Holm, L. W., & Skillgate, E. (2017). Are job strain and sleep disturbances prognostic factors for low-back pain? A cohort study of a general population of working age in Sweden. *J Rehabil Med*, *49*(7), 591-597. | Outcome criteria not fulfilled. |
| 144 | Reme, S. E., Shaw, W. S., Boden, L. I., Tveito, T. H., O'Day, E. T., Dennerlein, J. T., & Sorensen, G. (2014). Worker assessments of organizational practices and psychosocial work environment are associated with musculoskeletal injuries in hospital patient care workers. *Am J Ind Med*, *57*(7), 810-818. | Study design criteria not fulfilled. |
| 145 | Ricco, M., Pezzetti, F., & Signorelli, C. (2017). Back and neck pain disability and upper limb symptoms of home healthcare workers: A case-control study from Northern Italy. *Int J Occup Med Environ Health*, *30*(2), 291-304. | Outcome criteria not fulfilled. |
| 146 | Ropponen, A., Narusyte, J., Silventoinen, K., & Svedberg, P. (2020). Health behaviours and psychosocial working conditions as predictors of disability pension due to different diagnoses: a population-based study. *BMC Public Health*, *20*(1), 1507. | Outcome criteria not fulfilled. |
| 147 | Ropponen, A., Samuelsson, A., Alexanderson, K., & Svedberg, P. (2013). Register-based data of psychosocial working conditions and occupational groups as predictors of disability pension due to musculoskeletal diagnoses: a prospective cohort study of 24,543 Swedish twins. *BMC Musculoskelet Disord*, *14*, 268. | Outcome criteria not fulfilled. |
| 148 | Roy, T. C., Lopez, H. P., & Adams. (2013). A comparison of deployed occupational tasks performed by different types of military battalions and resulting low back pain. *Mil Med*, *178*(8), e937-943. | Outcome criteria not fulfilled. |
| 149 | Roy, T. C., Lopez, H. P., & Piva, S. R. (2013). Loads worn by soldiers predict episodes of low back pain during deployment to Afghanistan. *Spine (Phila Pa 1976)*, *38*(15), 1310-1317. | Outcome criteria not fulfilled. |
| 150 | Roy, T. C., Piva, S. R., Christiansen, B. C., Lesher, J. D., Doyle, P. M., Waring, R. M., Irrgang, J. J., Moore, C. G., Brininger, T. L., & Sharp, M. A. (2016). Heavy Loads and Lifting are Risk Factors for Musculoskeletal Injuries in Deployed Female Soldiers. *Mil Med*, *181*(11), e1476-e1483. | Outcome criteria not fulfilled. |
| 151 | Rufa'i, A. A., Sa'idu, I. A., Ahmad, R. Y., Elmi, O. S., Aliyu, S. U., Jajere, A. M., & Digil, A. A. (2015). Prevalence and Risk Factors for Low Back Pain Among Professional Drivers in Kano, Nigeria. *Arch Environ Occup Health*, *70*(5), 251-255. | Study design criteria not fulfilled. |
| 152 | Runeson-Broberg, R., Lindgren, T., & Norback, D. (2014). Musculoskeletal symptoms and psychosocial work environment, among Swedish commercial pilots. *Int Arch Occup Environ Health*, *87*(7), 685-693. | Study design criteria not fulfilled. |
| 143 | Sadeghi-Yarandi M, Ghasemi M, Ghanjal A, Sepandi M, Soltanzadeh A. The Prediction of Chronicity in Patients With Acute and Subacute Nonspecific Low Back Pain and Associated Risk Factors: A Case-Control Study. Pain Manag Nurs. 2022. | No measure of an association. |
| 154 | Sadeghian, F., Coggon, D., Ntani, G., & Hosseinzadeh, S. (2015). Predictors of low back pain in a longitudinal study of Iranian nurses and office workers. *Work*, *51*(2), 239-244. | Outcome criteria not fulfilled. |
| 155 | Sain, M. K., & Meena, M. (2019). Identifying musculoskeletal issues and associated risk factors among clay brick kiln workers. *Ind Health*, *57*(3), 381-391. | Study design criteria not fulfilled. |
| 156 | Sain, M. K., & Meena, M. L. (2018). Exploring the musculoskeletal problems and associated risk-factors among brick kiln workers. *International Journal of Workplace Health Management*, *11*(6), 395-410. | Study design criteria not fulfilled. |
| 157 | Salve, U. R. (2015). Prevalence of musculoskeletal discomfort among the workers engaged in jewelry manufacturing. *Indian J Occup Environ Med*, *19*(1), 44-55. | Other reasons (e.g., abstract, books). |
| 158 | Salo S, Hurri H, Rikkonen T, Sund R, Kroger H, Sirola J. Association between severe lumbar disc degeneration and self-reported occupational physical loading. J Occup Health. 2022;64(1):14. | Study design criteria not fulfilled. |
| 159 | Samaei, S. E., Mostafaee, M., Jafarpoor, H., & Hosseinabadi, M. B. (2017). Effects of patient-handling and individual factors on the prevalence of low back pain among nursing personnel. *Work*, *56*(4), 551-561. | Study design criteria not fulfilled. |
| 160 | Sauter M, Barthelme J, Müller C, Liebers F. Manual handling of heavy loads and low back pain among different occupational groups: results of the 2018 BIBB/BAuA employment survey. BMC Musculoskeletal Disorders. 2021;22(1):1-14. | Study design criteria not fulfilled. |
| 161 | Schlussel, A. T., & Maykel, J. A. (2019). Ergonomics and Musculoskeletal Health of the Surgeon. *Clin Colon Rectal Surg*, *32*(6), 424-434. | Other reasons (e.g., abstract, books). |
| 162 | Serranheira, F., Sousa-Uva, M., Heranz, F., Kovacs, F., & Sousa-Uva, A. (2020). Low Back Pain (LBP), work and absenteeism. *Work*, *65*(2), 463-469. | Study design criteria not fulfilled. |
| 163 | Shankar, S., Naveen Kumar, R., Mohankumar, P., & Jayaraman, S. (2017). Prevalence of work-related musculoskeletal injuries among South Indian hand screen-printing workers. *Work*, *58*(2), 163-172. | Study design criteria not fulfilled. |
| 164 | Shankar, S., Shanmugam, M., & Srinivasan, J. (2015). Workplace factors and prevalence of low back pain among male commercial kitchen workers. *J Back Musculoskelet Rehabil*, *28*(3), 481-488. | Study design criteria not fulfilled. |
| 165 | Shiri, R., Falah-Hassani, K., Heliovaara, M., Solovieva, S., Amiri, S., Lallukka, T., Burdorf, A., Husgafvel-Pursiainen, K., & Viikari-Juntura, E. (2019). Risk Factors for Low Back Pain: A Population-Based Longitudinal Study. *Arthritis Care Res (Hoboken)*, *71*(2), 290-299. | Outcome criteria not fulfilled. |
| 166 | Silva, C., Barros, C., Cunha, L., Carnide, F., & Santos, M. (2016). Prevalence of back pain problems in relation to occupational group. *International Journal of Industrial Ergonomics*, *52*, 52-58. | Study design criteria not fulfilled. |

| | | |
|---|---|---|
| 167 | Skillgate E, Isacson Hjortzberg M, Strömwall P, Hallqvist J, Onell C, Holm LW, et al. Non-Preferred Work and the Incidence of Spinal Pain and Psychological Distress-A Prospective Cohort Study. Int J Environ Res Public Health. 2021;18(19). | Outcome criteria not fulfilled. |
| 168 | Solecki, L. (2014). Complaints of low back pain among private farmers exposed to whole body vibration. *Med Pr*, *65*(1), 55-64. | Other reasons (e.g., abstract, books). |
| 169 | Solovev, A., Watanabe, Y., Kitamura, K., Takahashi, A., Kobayashi, R., Saito, T., Takachi, R., Kabasawa, K., Oshiki, R., Platonova, K., Tsugane, S., Iki, M., Sasaki, A., Yamazaki, O., Watanabe, K., & Nakamura, K. (2020). Total physical activity and risk of chronic low back and knee pain in middle-aged and elderly Japanese people: The Murakami cohort study. *Eur J Pain*, *24*(4), 863-872. | Exposure criteria not fulfilled. |
| 170 | Sterud, T., Johannessen, H. A., & Tynes, T. (2016). Do Work-Related Mechanical and Psychosocial Factors Contribute to the Social Gradient in Low Back Pain?: A 3-Year Follow-Up Study of the General Working Population in Norway. *Spine (Phila Pa 1976)*, *41*(13), 1089-1095. | Outcome criteria not fulfilled. |
| 171 | Sterud, T., & Tynes, T. (2013). Work-related psychosocial and mechanical risk factors for low back pain: a 3-year follow-up study of the general working population in Norway. *Occup Environ Med*, *70*(5), 296-302. | Outcome criteria not fulfilled. |
| 172 | Stevens, M. L., Boyle, E., Hartvigsen, J., Mansell, G., Sogaard, K., Jorgensen, M. B., Holtermann, A., & Rasmussen, C. D. N. (2019). Mechanisms for reducing low back pain: a mediation analysis of a multifaceted intervention in workers in elderly care. *Int Arch Occup Environ Health*, *92*(1), 49-58. | Outcome criteria not fulfilled. |
| 173 | Sundstrup, E., & Andersen, L. L. (2017). Hard Physical Work Intensifies the Occupational Consequence of Physician-Diagnosed Back Disorder: Prospective Cohort Study with Register Follow-Up among 10,000 Workers. *Int J Rheumatol*, *2017*, 1037051. | Outcome criteria not fulfilled. |
| 174 | Tang, R., Kapellusch, J. M., Hegmann, K. T., Thiese, M. S., Wang, I., & Merryweather, A. S. (2020). Evaluating Different Measures of Low Back Pain Among U.S. Manual Materials Handling Workers: Comparisons of Demographic, Psychosocial, and Job Physical Exposure. *Hum Factors*, 18720820971101. | Outcome criteria not fulfilled. |
| 175 | Telaprolu, N., & Anne, S. D. (2014). Physical and psychological work demands as potential risk factors for musculoskeletal disorders among workers in weaving operations. *Indian JOccup Environ Med*, *18*(3), 129-134. | Study design criteria not fulfilled. |
| 176 | Thiede, M., Liebers, F., Seidler, A., Gravemeyer, S., & Latza, U. (2014). Gender specific analysis of occupational diseases of the low back caused by carrying, lifting or extreme trunk flexion--use of a prevention index to identify occupations with high prevention needs. *Am J Ind Med*, *57*(2), 233-244. | Study design criteria not fulfilled. |
| 177 | Thiese, M. S., Lu, M. L., Merryweather, A., Tang, R., Ferguson, S. A., Malloy, E. J., Marras, W. S., Hegmann, K. T., & Kapellusch, J. (2020). Psychosocial Factors and Low Back Pain Outcomes in a Pooled Analysis of Low Back Pain Studies. *J Occup Environ Med*, *62*(10), 810-815. | Study design criteria not fulfilled. |
| 178 | Udom, C., Kanlayanaphotporn, R., & Janwantanakul, P. (2019). Predictors for Nonspecific Low Back Pain in Rubber Farmers: A 1-Year Prospective Cohort Study. *Asia Pac J Public Health*, *31*(1), 7-17. | Outcome criteria not fulfilled. |
| 179 | Upadhyay R, Bhattacherjee A, Patra AK, Chau N. Association between Whole-Body Vibration exposure and musculoskeletal disorders among dumper operators: A case-control study in Indian iron ore mines. Work. 2022;71(1):235-47. | Outcome criteria not fulfilled. |
| 180 | Urquhart, D. M., Kelsall, H. L., Hoe, V. C., Cicuttini, F. M., Forbes, A. B., Sim, M. R., & Burton. (2013). Are psychosocial factors associated with low back pain and work absence for low back pain in an occupational cohort? *Clin J Pain*, *29*(12), 1015-1020. | Study design criteria not fulfilled. |
| 181 | Vargas-Prada, S., Serra, C., Martinez, J. M., Ntani, G., Delclos, G. L., Palmer, K. T., Coggon, D., & Benavides, F. G. (2013). Psychological and culturally-influenced risk factors for the incidence and persistence of low back pain and associated disability in Spanish workers: findings from the CUPID study. *Occup Environ Med*, *70*(1), 57-62. | Outcome criteria not fulfilled. |
| 182 | Vieira LA, Dos Santos AA, Peluso C, et al. Influence of lifestyle characteristics and VDR polymorphisms as risk factors for intervertebral disc degeneration: a case-control study. Eur J Med Res 2018;23(1):11. doi: 10.1186/s40001-018-0309-x. | Exposure criteria not fulfilled. |
| 183 | Vinstrup, J., Jakobsen, M. D., & Andersen, L. L. (2020). Perceived Stress and Low-Back Pain Among Healthcare Workers: A Multi-Center Prospective Cohort Study. *Front Public Health*, *8*, 297. | Outcome criteria not fulfilled. |
| 184 | Vinstrup, J., Jakobsen, M. D., Madeleine, P., & Andersen, L. L. (2020). Physical exposure during patient transfer and risk of back injury & low-back pain: prospective cohort study. *BMC Musculoskelet Disord*, *21*(1), 715. | Outcome criteria not fulfilled. |
| 185 | Wahlstrom J, Burstrom L, Johnson PW, et al. Exposure to whole-body vibration and hospitalization due to lumbar disc herniation. Int Arch Occup Environ Health 2018;91(6):689- 94. doi: 10.1007/s00420-018-1316-5. | Exposure criteria not fulfilled. |
| 186 | Wang F, Chen K, Lin QS, Ma YG, Huang H, Wang CAF, et al. Earlier or heavier spinal loading is more likely to lead to recurrent lumbar disc herniation after percutaneous endoscopic lumbar discectomy. J Orthop Surg Res. 2022;17(1):7. | Outcome criteria not fulfilled. |
| 187 | Wippert PM, Valencia LP, Driesslein D. Stress and Pain. Predictive (Neuro)Pattern Identification for Chronic Back Pain: A Longitudinal Observational Study. Front Med. 2022;9:14. | Outcome criteria not fulfilled. |
| 188 | Wixted, F., & O'Sullivan, L. (2019). Task engagement as a mediator between the cognitive demands of sustained attention and musculoskeletal complaints: A structural equation modelling approach. *Work*, *64*(3), 623-634. | Study design criteria not fulfilled. |
| 189 | Wurzelbacher, S. J., Lampl, M. P., Bertke, S. J., & Tseng, C. Y. (2020). The effectiveness of ergonomic interventions in material handling operations. *Appl Ergon*, *87*, 103139. | Outcome criteria not fulfilled. |
| 190 | Xiao, H., McCurdy, S. A., Stoecklin-Marois, M. T., Li, C. S., & Schenker, M. B. (2013). Agricultural work and chronic musculoskeletal pain among Latino farm workers: the MICASA study. *Am J Ind Med*, *56*(2), 216-225. | Study design criteria not fulfilled. |

| | | |
|---|---|---|
| 191 | Yang, S. T., Park, M. H., & Jeong, B. Y. (2020). Types of manual materials handling (MMH) and occupational incidents and musculoskeletal disorders (MSDs) in motor vehicle parts manufacturing (MVPM) industry. *International Journal of Industrial Ergonomics*, *77*, 9. | Study design criteria not fulfilled. |
| 192 | Yazgan E, Ozkan NF, Ulutas BH. A questionnaire-based musculoskeletal disorder assessment for aircraft maintenance technicians. Aircr Eng Aerosp Technol. 2022;94(2):240-7. | Outcome criteria not fulfilled. |
| 193 | Yovi, E. Y., & Prajawati, W. (2015). High Risk Posture on Motor-Manual Short Wood Logging System in Acacia mangium Plantation. *Jurnal Manajemen Hutan Tropika (Journal of Tropical Forest Management)*, *21*(1), 11-18. | Study design criteria not fulfilled. |
| 194 | Yovi, E. Y., & Yamada, Y. (2019). Addressing Occupational Ergonomics Issues in Indonesian Forestry. *Croatian Journal of Forest Engineering*, *40*(2), 351-363. | Study design criteria not fulfilled. |
| 195 | Yue, P., Xu, G., Li, L., & Wang, S. (2014). Prevalence of musculoskeletal symptoms in relation to psychosocial factors. *Occup Med (Lond)*, *64*(3), 211-216. | Study design criteria not fulfilled. |
| 196 | Zamri, E. N., Hoe, V. C. W., & Moy, F. M. (2020). Predictors of low back pain among secondary school teachers in Malaysia: a longitudinal study. *Ind Health*, *58*(3), 254-264. | Outcome criteria not fulfilled. |
| 197 | Zarra, T., & Lambrianidis, T. (2014). Musculoskeletal disorders amongst Greek endodontists: a national questionnaire survey. *Int Endod J*, *47*(8), 791-801. | Study design criteria not fulfilled. |
| 198 | Zhang, D., & Huang, H. (2017). Prevalence of work-related musculoskeletal disorders among sonographers in China: results from a national web-based survey. *J Occup Health*, *59*(6), 529-541. | Study design criteria not fulfilled. |
| 199 | Zhang, M. Y., Bai, Z. Z., Zhao, X. F., Ieee, Ieee, & Dalian Univ Technol, F. I. E. D. C. M. D. P. R. C. (2017). Real-time Risk Assessment for Construction Workers' Trunk Posture Using Mobile Sensor. *International Conference on Robotics and Automation Sciences (ICRAS)*, 153-157. | Study design criteria not fulfilled. |

# **Appendix F**. Study characteristics

**Table S10**. Description of each included study

| Author | Design | Population | Outcome | | Exposure | |
|---|---|---|---|---|---|---|
| | | | **Definition** | **Assessment** | **Definition** | **Assessment** |
| **Aghilinejad 2015 (27)** | Cohort | The cohort consisted of male workers in one of the biggest metal-industry factories in Iran followed from 2012 to 2013. In total, 218 workers received a questionnaire and 33 were excluded. Therefore, 185 workers were eligible for the analysis (49 chronic participants and 136 cured for acute LBP) with a mean age of 35.96 (SD=7.33). | LBP: Self-reported chronic pain ≥3 months. Participants are followed up until 3 months after onset during monthly phone calls. If the pain had ended before the 3 months, they were categorised in the acute group. | Interview. | Psychosocial exposures were defined as: job demand, job control, job satisfaction, social support, and job strain.<br>All exposures were dichotomised. | Questionnaire (MUSIC)*. |
| **Ahsan 2013 (28)** | Case-control | 240 cases with LDH were recruited by their physicians from a spinal surgery unit in Dhaka, Bangladesh (2007-2010). 200 cases (124 males and 76 females) were eligible, and 200 controls were matched on age, sex, and area of residence from a non-spinal related orthopaedic department. The overall mean age was 39.42 years (SD=NS). | LDH: With low back pain with or without sciatica collected from radiological and physicians' examinations.<br>Diagnostic criteria:<br>- Dominant leg pain than back pain.<br>- Restricted Straight Leg Raise.<br>- Neurological deficit.<br>- Positive MRI findings. | Physical examination, radiological examination, and MRI. | Psychosocial exposures comprised job satisfaction and stress at work. | Interview. |
| **Esquirol 2017 (29)** | Cohort | 3,237 male and female employed and retired workers from south of France were followed with a 5-year follow-up period with age ranging from 32 to 52 years. For the analysis, 804 males and 756 females were eligible and divided in:<br>- 231 participants reported "persistent chronic LBP" (they were compared with a non-persistent chronic LBP group containing 199 participants)<br>- 255 participants reported "incidence of chronic LBP" (they were compared with a non-chronic LBP group containing 875 participants). | LBP: Participants were considered as suffering from chronic LBP if they reported LBP or a specific treatment for such pain for at least 6 months at both measurements' points.<br><br>Participants are divided in three outcome groups:<br>1. Answering "no" to CLBP at both measurements.<br>2. Incidence CLBP – answering "no" at baseline but "yes" at follow-up.<br>3. Persistence CLBP – answering "yes" at both measurements. | Questionnaire. | Psychosocial exposures were defined using the Karasek model to categories levels of decision latitude and demands. Furthermore, three items were dichotomised: job recognition, occupational support, and difficulty communicating. | Questionnaire. |
| **Gold 2017 (30)** | Cohort | The study consisted of workers from multiple facilities within a single company in USA; with the vast majority being clinically staff, specifically nurses and nursing aids with a mean age of 41.1 years (SD=13.1).<br>1291 responded to the survey after 2 years with 1154 participants being eligible for the analyses (>90 % females), and after 6 years, 228 participants were left after attrition. | LBP: Was defined as pain in the low back region the past 3 months with at least mild severity during the prior week. | Questionnaire. | Psychosocial exposures comprised work-family imbalance. | Questionnaire (JCQ)*. |

| | | | | | | |
|---|---|---|---|---|---|---|
| **Halonen 2018 (31)** | Cohort | A representative Swedish working population consisting of 9,756 individuals. Of these, 4,079 participants were included based on answers to at least 3 out of 4 survey rounds. For the analyses, 1,845 had onset of LBP with (932 males and 913 females) with age NS. | LBP: Self-reported pain in the last 3 months defined as either "pain that affects my life a little" or "pain that affects my life a lot".<br><br>LBP was dichotomised into:<br>- No affecting pain (no pain or pain that does not affect life).<br>- Affecting pain (pain affecting life a little or a lot). | Questionnaire. | Psychosocial exposures comprised the effort-reward imbalance. It consisted of 10 effort-reward items: three related to efforts at work and seven related to rewards. An effort-reward ratio was calculated and values below 1 indicated no effort-reward imbalance and $\geq$1 indicated perception of effort-reward imbalance. | Questionnaire. |
| **Herin 2014 (32)** | Cohort | Representative sample of subjects randomly selected from 7 French regions using exhaustive lists under the supervision of 400 volunteering occupational physicians. A total of 21,378 participants were included at baseline, 18,695 responded at follow-up, and 12,591 was eligible both at baseline and follow-up. For the analyses, 1206 participants (787 males and 419 females) were presented with LBP who came from 4 years of birth (1938, 1943, 1948 and 1953). | LBP: Self-reported musculoskeletal pain in combination with physicians' examination. Chronic musculoskeletal pain was defined as subjects who, on the day of examination, declared low back pain for at least 6 months who also presented with positive clinical signs. | Interview and physical examination. | Psychosocial exposures comprised psychological demands and decision latitude, both proxies of the two fundamental psychosocial factors of the Karasek model. | Questionnaire. |
| **Jansen 2004 (33)** | Cohort | The cohort consisted of workers from 7 Dutch nursing homes and homes for elderly with various professions such as nurses, care givers, kitchen workers, transportation etc.<br>1208 subjects were invited to participate in 1998-1999 and 769 agreed to participate. After 1 year, 523 were observed again with a mean age of 40.7 years of age (SD=9.7).<br>Information on sex was not provided. | LBP: Low back pain with disability was defined by Von Korff's disability score >50 points which indicated "high disability". | Questionnaire. | Psychosocial exposures comprised decision authority, skill discretion, and work demands included in the Karasek model. | Questionnaire |
| **Jørgensen 2013 (34)** | Cohort | The cohort consisted of Danish participants employed in 14 private and public companies. 5,249 men took part in the baseline examination. 3,833 were without LBP at baseline and was then included for the analyses aged between 40 and 59 years. | LDH: Hospitalisation due to herniated lumbar disc disease was identified in the National Hospital Register between 1977 and 2003 using the ICD-8 code 725.11 and ICD-10 code M51.1. | Register information. | Psychosocial exposures comprised mental stress at work - seldom or regularly. | Questionnaire |
| **Latza 2002 (35)** | Cohort | The cohort consisted of 571 male construction workers (age 17-59 years) from Hamburg, Germany. After 3 years, all workers were approached for a follow-up survey and 488 were willing to participate with a mean age of 33.1 years (SD=10.0). | Chronic LBP: $\geq$90 days of low back pain during the last 12 months. | Interview. | Psychosocial exposures comprised monotonous work, time pressure, low job control, poor social support, and satisfaction with own achievements, all measured on a 5-point Likert scale. | Interview |
| **Matsudaira 2014 (36)** | Cohort | Employees were recruited from 16 local offices (e.g., office workers, nurses, salesmen and manufacturing engineers) in/near Tokyo, Japan. | Persistent LBP: LBP interfering with work (grade 2 or 3) with disability lasting longer than 3 months during a 1-year follow-up period. | Questionnaire. | Psychosocial exposures comprised mental workload, interpersonal stress at work, workplace environment stress, job control, utilization of skills and expertise, and | Questionnaire (BJSQ)*. |

| | | | | | | |
|---|---|---|---|---|---|---|
| | | Baseline questionnaire was distributed to 6,140 participants and 5,310 responded. After 1 year, 3,811 participants completed the follow-up questionnaire.<br>Among the 3,811 participants, 1,675 reported mild LBP during the past year at baseline with a mean age of 43.1 years (SD=10.1) and 78.6% males. Of these, 43 participants reported persistent LBP within the 1-year follow-up period. | Grades:<br>- Grade: No LBP.<br>- Grade 1: LBP that does not interfere with work.<br>- Grade 2: LBP that interferes with work but no absence from work.<br>- Grade 3: LBP that interferes with work, leading to sick leave. | | reward to work. Each exposure was assessed on a five-point scale. | |
| **Matsudaira 2015 (37)** | Cohort | Employees were recruited from 16 local offices (e.g., office workers, nurses, salesmen and manufacturing engineers) in/near Tokyo, Japan.<br>Baseline questionnaire was distributed to 6140 participants and 5,310 responded. After 1 year, 3,811 participants completed the follow-up questionnaire.<br>Among the 3,811 employees, 171 reported LBP and experiencing work interferences with or without sick leave during a month prior to baseline with a mean age of 42.9 years (SD=10.1) (71.4% males). Of these, 29 developed chronic disabling LBP during a year prior to the follow-up period. | Chronic disabling LBP: LBP that interfered with work for >3 months, regardless of sick leave (grade 2 and 3) during a 1-year follow-up period.<br><br>Grades:<br>- Grade: No LBP.<br>- Grade 1: LBP that does not interfere with work.<br>- Grade 2: LBP that interferes with work but no absence from work.<br>- Grade 3: LBP that interferes with work, leading to sick leave. | Questionnaire. | Psychosocial exposures comprised mental workload, interpersonal stress at work, workplace environment stress, job control, utilization of skills and expertise, and reward to work. Each exposure was assessed on a five-point scale. | Questionnaire (BJSQ)*. |
| **Matsudaira 2019 (38)** | Cohort | Participants were recruited from different occupational groups in/near Tokyo. Occupational groups varied from nurses, office workers, sales/marketing to transportation. Baseline questionnaire was distributed to 3,187 employees and 2651 returned the questionnaire. Of these, 1809 participants returned the completed follow-up questionnaire.<br>Among the 1809 participant, only 198 participants with disabling LBP during the month before baseline were included. The mean age was 36.0 (SD=9.1) where 69% were males. Of these 198, 35 had chronic disabling LBP during the 1-year follow-up period. | Chronic disabling LBP: LBP that interfered with work for >3 months regardless of sick leave (grade 2 and 3) during the 1-year follow-up period.<br><br>Grades:<br>- Grade 0: No LBP.<br>- Grade 1: LBP that does not interfere with work.<br>- Grade 2: LBP that interferes with work but no absence from work.<br>- Grade 3: LBP that interferes with work, leading to sick-leave. | Questionnaire. | Psychosocial exposures comprised interpersonal stress at work, inadequate breaks at work, lack of control over how to work, lack of control over what to do at work, lack of workplace support, and dissatisfied with job | Questionnaire (CUPID). |
| **Melloh 2013 (39)** | Cohort | 315 patients were recruited from 14 health practitioners across New Zealand due to their first episode of acute to subacute LBP or for recurrent LBP. 147 patients were lost to follow-up resulting in 168 patients participating over the six-months period (62% females) with a mean age of 36 (SD=13.1). | To determine persistent LBP, follow-up questionnaires were sent after 3, 6, and 12 weeks and at 6 month collecting information on pain intensity (VAS-scale) in the low back during the last week. Patients with persistent LBP were then compared to patients with non-persistent LBP – no information was given on how the groups were categorised. | Questionnaire. | Psychosocial exposures comprised job satisfaction, job control and social support at work based on the Multinational Musculoskeletal Inception Cohort Study statements. | Questionnaire (items from JCQ). |

| | | | | | | |
|---|---|---|---|---|---|---|
| **Melloh 2013 (40)** | Cohort | 315 patients were recruited from 14 health practitioners in New Zealand due to their first episode of acute to subacute LBP or for recurrent LBP. 168 participants (62 % females) completed the follow-up with a mean age of 36.0 (SD=13.1). | Persistent low back pain was defined by Oswestry Disability Index scorer of >10 points at baseline and 6 months and an ODI change score of ≤10 points between baseline and 6-month follow-up. | Questionnaire. | Psychosocial exposures comprised job satisfaction, job control and social support at work based on the Multinational Musculoskeletal Inception Cohort Study statements. | Questionnaire (items from JCQ). |
| **Melloh 2013 (41)** | Cohort | 315 patients were recruited from 14 health practitioners in New Zealand due to their first episode of acute to subacute LBP or for recurrent LBP. 195 participants (62 % females) completed the follow-up with a mean age of 36.0 (SD=13.1). | Persistent low back pain was defined by Oswestry Disability Index scorer of >10 points, measured at baseline and 3, 6, and 12 weeks. An ODI score of >10 points and under a 10-point change score at 12-week follow-up was considered as persistent LBP. | Questionnaire. | Psychosocial exposures comprised job satisfaction based on a combined score (4-28 points) and social support at work based on a combined score (6-30 points). | Questionnaire (items from JCQ). |
| **Seidler 2003 (42)** | Case-control | 437 male patients recruited at neurological clinics in Germany aged between 25 and 65 years. After exclusion, 225 cases were eligible and divided into:<br>- LDH with osteochondrosis/spondylosis = 131, mean age 43.7<br>- “Pure” LDH = 94, mean age of 40.0.<br><br>107 controls (males) were from a random population group (mean age of 43) and 90 patients (males) admitted to hospital for urolithiasis who had no radiographically confirmed osteochondrosis or spondylosis (mean age of 40). | Herniation of the lumbar discs or osteochondrosis/spondylosis of the lumbar spine associated with chronic complaints (low back pain, sciatica). The diagnosis of lumbar disc herniation had to have been confirmed by computed tomography or magnetic resonance imaging.<br><br>The radiographs were reassessed by reference radiologist. | MRI and CT. | Psychosocial exposures comprised monotonous work, opportunities to use knowledge and skills, satisfaction with supervisor, satisfaction with workmates, psychic strain, time pressure, and to much responsibility. The psychosocial exposures were assessed on a scale from 1 to 6. | Interview. |
| **Seyedmehdi 2016 (43)** | Cohort | All industrial workers from a large Iranian rubber factory with acute non-specific LBP in the past 2 weeks were included (2011-2912). Diagnosis of acute non-specific LBP at baseline was made by two occupational medicine specialists. The cohort consisted of 542 participants and 511 completed the 1-year follow-up (500 males and 11 females) with a mean age of 37.6 years (SD=5.8). | LBP was assessed 3, 6, 9 months and at 1 year after baseline asking the question: “Have you recovered from your LBP”. If the answer was “yes”<br>The question would follow: “How long did it last?”<br><br>Participants with LBP were divided in 2 groups:<br>1. LBP lasting <3 months.<br>2. LBP lasting ≥3 months. | Face-to-face or telephone interview. | Psychosocial exposures comprised job stress, evaluated by the factors decision making, psychological demands, social support, and occupational and physical needs, and dichotomised into high/low stress. | Questionnaire (JCQ). |
| **Sihawong 2016 (44)** | Cohort | The cohort consisted of a sample of office workers recruited from nine-scale enterprises in Thailand. 3,446 office workers responded to the questionnaire and 2,483 were excluded. 669 agreed to participate in the physical examination and 615 were followed up after 1 year.<br>Due to incomplete data, 609 were included in the analysis with a mean age of 35.7 (SD=8.3) including 168 males and 501 females. | Incidence of chronic low back pain was defined as ongoing pain greater than 3 months over the past 6 months. Participants received a self-administered diary to record low back pain, and researchers would collect the diaries every month over the course of 12 months. | Diaries. | Psychosocial exposures comprised psychological job demands measured by factors of decision latitude, support, demands, and security. | Questionnaire (JCQ). |
| **Sørensen 2011 (45)** | Cohort | Participants were employed at 14 different private and public companies in Copenhagen, Denmark, including railway, insurance, fire brigade etc., aged between 40 to 59 years. 5249 | Hospitalisation due to herniated lumbar disc disease was identified in the National Hospital Register between 1977 and 2003 using the ICD-8 code 725.11 and ICD-10 code M51.1. | Register information. | Psychosocial exposures comprised mental stress at work measured by “seldom” or “regularly”. | Questionnaire. |

| | | | | | | |
|---|---|---|---|---|---|---|
| | | males were invited at baseline and 3833 without LBP at baseline were entered in the study. | | | | |
| **Tubach 2004 (46)** | Cohort | At baseline, the cohort included 20,624 subjects who were employees at a French electricity and gas company. Of these, a random sample of 4,018 subjects received a questionnaire about LBP. 3,240 completed the questionnaire whereas 475 were included in the analyses (405 males and 70 females) aged between 35 to 50 years. | LBP: Assessed by the question regarding suffering from sciatica the last year and if the subjects had visited a physician regarding their symptoms. | Questionnaire. | Psychosocial exposures comprised job satisfaction measured with an analogic visual scale divided into tertiles. | Questionnaire. |

Abbreviations: CT = computed tomography; ICD = International Classification of Diseases; kg = kilogram; LBP = low back pain; LDH = lumbar disc herniation; LDN = lumbar disc narrowing; MRI = magnetic resonance imaging; MS = milliseconds; NS = not specified; SD = standard deviation. *BJSQ = Brief Job Stress Questionnaire; CUPID = Cultural and Psychosocial Influences on Disability; JCQ = Job Content Questionnaire; MUSIC = Musculoskeletal intervention Center-Norrtälje Study.

## **Appendix G**. Measure of associations

**Table S11**. Measures of associations of each included study

| Author | Exposure | Confounders | Categories of exposure | Results: Men — Measure of association | Men — 95% CI | Women — Measure of association | Women — 95% CI | All — Measure of association | All — 95% CI |
|---|---|---|---|---|---|---|---|---|---|
| **Job control** | | | | | | | | | |
| Aghilinejad 2015 | *Job control*: Was assessed using Karasek model and measured by 6 questions on a four-point scale and dichotomised (N=185, 126 cases and 59 controls). | None. | - No | 1.00 OR | - | - | - | - | - |
| | | | - Yes | 0.84 OR | 0.42 – 1.68 | - | - | - | - |
| Herin 2014 | *Decision latitude*: Was assessed using Karasek model and considered "low" when answering "no" to two out of three questions regarding room for learning, variety of work, and choosing how to do one's work (N=1,206, 787 males and 419 females). | Age, sports participation, BMI, and social class. | Men | 1.00 OR | - | 1.00 OR | - | - | - |
| | | | - Low | 1.06 OR | 0.90 – 1.26 | 0.91 OR | 0.71 – 1.16 | - | - |
| | | | - High | | | | | | |
| | | | Women | | | | | | |
| | | | - Low | | | | | | |
| | | | - High | | | | | | |
| Jansen 2004 | *Decision authority*: was assessed using Karasek's model with 11 questions reflecting planning, influence on pace, pauses etc. and based on the centile of distribution (N=523). | Age, trunk flexion between 20 to 45º, trunk flexion >45º, and lifting/carrying loads >10 kg, skill discretion, work demands, and years in service | - 10th centile | - | - | - | - | 1.00 RR | - |
| | | | - 25th centile | - | - | - | - | 1.07 RR | 0.64 – 1.78 |
| | | | - 50th centile | - | - | - | - | 1.13 RR | 0.42 – 3.04 |
| | | | - 75th centile | - | - | - | - | 1.50 RR | 0.55 – 4.14 |
| | | | - 90th centile | - | - | - | - | 0.76 RR | 0.21 – 2.72 |
| | *Skill discretion*: was assessed using Karasek's model with 6 questions reflecting skills, task variety, learning etc. and based on the centile of distribution (N=523). | Age, trunk flexion between 20 to 45º, trunk flexion >45º, lifting/carrying loads >10 kg, decision authority, work demands, and years in service | - 10th centile | - | - | - | - | 1.00 RR | - |
| | | | - 25th centile | - | - | - | - | 1.10 RR | 0.63 – 1.89 |
| | | | - 50th centile | - | - | - | - | 1.22 RR | 0.45 – 3.31 |
| | | | - 75th centile | - | - | - | - | 1.44 RR | 0.53 – 3.91 |
| | | | - 90th centile | - | - | - | - | 1.09 RR | 0.38 – 3.16 |
| Latza 2002 | *Job control*: Was measured by the question "Regulations and instructions hinder my performance very much" using a 5-point Likert scale (without chronic LBP N=404 at baseline and all workers N= 488). | Age. | Without chronic LBP at baseline | | | | | | |
| | | | - High | 1.00 PR | - | - | - | - | - |
| | | | - Medium | 1.48 PR | 0.53 – 4.12 | - | - | - | - |
| | | | - Low | 1.13 PR | 0.40 – 3.20 | - | - | - | - |
| | | | All workers | | | | | | |
| | | | - High | 1.00 PR | - | - | - | - | - |
| | | | - Medium | 1.45 PR | 0.71 – 2.96 | - | - | - | - |
| | | | - Low | 1.39 PR | 0.69 – 2.83 | - | - | - | - |
| Matsudaira 2015 | *Control*: Job control was assessed with the BJSQ on a five-point Likert scale and dichotomised (N=119, 4 controlled and 115 not controlled) | None. | - Controlled | | - | - | - | 1.00 OR | - |
| | | | - Not controlled | | - | - | - | 1.81 OR | 0.69 – 4.79 |

| | | | | | | | | | |
|---|---|---|---|---|---|---|---|---|---|
| Matsudaira 2019 | *Control*: Lack of control over how to work was assessed with the CUPID-Questionnaire on a five-point scale and dichotomised (N=198). | None. | - No<br>- Yes | -<br>- | -<br>- | -<br>- | -<br>- | 1.00 OR<br>1.03 OR | -<br>0.50 – 2.14 |
| | *Control*: Lack of control over what to do at work was assessed with the CUPID-Questionnaire on a five-point scale and dichotomised (N=198). | None. | - No<br>- Yes | -<br>- | -<br>- | -<br>- | -<br>- | 1.00 OR<br>1.08 OR | -<br>0.51 – 2.25 |
| Melloh 2013 | *Job control*: Was assessed using the JCQ and measured on a seven-point scale (N=168, 38 with persistent LBP and 130 with non-persistent LBP). | Age, sex, and BMI. | - No/low job control<br>- Job control | -<br>- | -<br>- | -<br>- | -<br>- | 1.00 OR<br>0.90 OR | -<br>0.55 – 1.50 |

## Job demand

| | | | | | | | | | |
|---|---|---|---|---|---|---|---|---|---|
| Aghilinejad 2015 | *Job demand*: Was assessed using Karasek model and measured by 5 questions on a four-point scale and dichotomised (N=185, 150 cases and 35 controls). | None. | - No<br>- Yes | 1.00 OR<br>1.94 OR | -<br>0.75 – 5.00 | -<br>- | -<br>- | -<br>- | -<br>- |
| Herin 2014 | *Demand*: Was assessed using Karasek model and psychological demand was measured by pace at work, interruption at work, and number of job tasks dichotomised (N=1,206, 787 males and 419 females). | Age, sports participation, BMI, and social class. | Men<br>- Low<br>- High<br>Women<br>- Low<br>- High | 1.00 OR<br>1.00 OR | -<br>0.87 – 1.15 | <br><br>1.00 OR<br>1.11 OR | <br><br>-<br>0.92 – 1.34 | <br><br>-<br>- | <br><br>-<br>- |
| Jansen 2004 | *Work demand:* was assessed using Karasek's model with 11 questions reflecting working fast, hard, excessive work etc. and based on the centile of distribution (N=523). | Trunk flexion between 20 to 45º, trunk flexion >45º, lifting/carrying loads >10 kg, decision authority, and skill discretion. | - 10th centile<br>- 25th centile<br>- 50th centile<br>- 75th centile<br>- 90th centile | -<br>-<br>-<br>-<br>- | -<br>-<br>-<br>-<br>- | -<br>-<br>-<br>-<br>- | -<br>-<br>-<br>-<br>- | 1.00 RR<br>0.86 RR<br>0.75 RR<br>1.41 RR<br>1.45 RR | -<br>0.61 – 1.21<br>0.33 – 1.73<br>0.63 – 3.18<br>0.60 – 3.53 |
| Sihawong 2016 | *Job demand*: Was assessed using the JCQ and measured by a seven-scale (N=609). | History of LBP, frequency of rest breaks, and frequency of exercise. | - Rate of chronic LBP | - | - | - | - | 1.12 OR | 0.99 – 1.26 |

## Job strain

| | | | | | | | | | |
|---|---|---|---|---|---|---|---|---|---|
| Aghilinejad 2015 | *Job strain*: Domains of job demands, job control and social support, and job satisfaction were summed, cut in the mid-point and divided into low and high (N=185, 45 cases and 140 controls). | None. | - No<br>- Yes | 1.000 OR<br>1.174 OR | -<br>0.56 – 2.48 | -<br>- | -<br>- | -<br>- | -<br>- |
| Esquirol 2017 | *Job strain*: Was assessed using Karasek model by combining levels of four dimensions based on psychological demands and decision latitude with low=low psych. and high decision, passive=low psych. and low decision, active=high psych. and | None. | Incidence chronic LBP:<br>- Low<br>- Passive<br>- Active<br>- High | <br>-<br>-<br>-<br>- | <br>-<br>-<br>-<br>- | <br>-<br>-<br>-<br>- | <br>-<br>-<br>-<br>- | <br>1.00 OR<br>1.05 OR<br>1.69 OR<br>0.95 OR | <br>-<br>0.59 – 1.88<br>0.81 – 3.55<br>0.55 – 1.63 |

| | | | | | | | | | |
|---|---|---|---|---|---|---|---|---|---|
| | high decision, and high=high psych. and low decision (N=1,130 for incidence and 430 for persistence). | | Persistence chronic LBP: | | | | | | |
| | | | - Low | - | - | - | - | 1.00 OR | - |
| | | | - Passive | - | - | - | - | 0.77 OR | 0.36 – 1.64 |
| | | | - Active | - | - | - | - | 1.30 OR | 0.49 – 3.45 |
| | | | - High | - | - | - | - | 0.67 OR | 0.34 – 1.35 |

# Job support

| | | | | | | | | | |
|---|---|---|---|---|---|---|---|---|---|
| Aghilinejad 2015 | *Social support*: Was assessed using the MUSIC-questionnaire and measured by 6 questions on a four-point scale and dichotomised (N=185, 49 cases and 136 controls). | Age, BMI, smoking, LBP history, family LBP history, education, shift working, job type, other physical and psychosocial factors. | - Low | 1.000 OR | - | - | - | - | - |
| | | | - High | 0.432 OR | 0.16 – 1.17 | - | - | - | - |
| Esquirol 2017 | *Support*: Occupational support was dichotomised (N=1,130 for incidence and 430 for persistence). | None. | Incidence chronic LBP: | | | | | | |
| | | | - No | - | - | - | - | 1.00 OR | - |
| | | | - Yes | - | - | - | - | 1.25 OR | 0.87 – 1.79 |
| | | | Persistence chronic LBP: | | | | | | |
| | | | - No | - | - | - | - | 1.00 OR | - |
| | | | - Yes | - | - | - | - | 1.15 OR | 0.74 – 1.81 |
| Latza 2002 | *Social support*: Was measured by the question “Colleagues impede my work” using a 5-point Likert scale (without chronic LBP N=404 at baseline and all workers N= 488). | Age. | Without chronic LBP at baseline | | | | | | |
| | | | - Low | 1.00 PR | - | - | - | - | - |
| | | | - Medium | 1.39 PR | 0.58 – 3.36 | - | - | - | - |
| | | | - High | 1.40 PR | 0.59 – 3.31 | - | - | - | - |
| | | | All workers | | | | | | |
| | | | - Low | 1.00 PR | - | - | - | - | - |
| | | | - Medium | 1.46 PR | 0.82 – 2.60 | - | - | - | - |
| | | | - High | 1.50 PR | 0.86 – 2.62 | - | - | - | - |
| Matsudaira 2015 | *Support by supervisors*: Was assessed with the BJSQ on a five-point scale and dichotomised (N=167). | None. | - Supported | - | - | - | - | 1.00 OR | - |
| | | | - Not supported | - | - | - | - | 2.00 OR | 0.88 – 4.55 |
| | *Support by co-workers*: Was assessed with the BJSQ on a five-point scale and dichotomised (N=168). | None. | - Supported | - | - | - | - | 1.00 OR | - |
| | | | - Not supported | - | - | - | - | 0.97 OR | 0.43 – 2.18 |
| Matsudaira 2014 | *Support by supervisors*: Was assessed with the BJSQ on a five-point scale and dichotomised (N=1,675). | Age, sex, obesity, smoking, education, and manual materials handling at work. | - Support | - | - | - | - | 1.00 OR | - |
| | | | - No support | - | - | - | - | 2.01 OR | 1.05 – 3.85 |
| Matsudaira 2019 | *Lack of workplace support*: Was assessed with the CUPID-Questionnaire (N=194). | None. | - Support | - | - | - | - | 1.00 OR | - |
| | | | - No support | - | - | - | - | 1.74 OR | 0.67 – 4.50 |
| Melloh 2013 | *Support*: Social support at work was assessed with the JCQ on a seven-point scale (N=168, 38 with persistent LBP and 130 with non-persistent LBP). | Age, sex, BMI, and somatization. | - No/low support | - | - | - | - | 1.00 OR | - |
| | | | - Social support | - | - | - | - | 0.67 OR | 0.45 – 0.99 |
| Melloh 2013 | *Social support*: Were assessed by questionnaires based on the recommendations of a multinational musculoskeletal cohort study addressing occupational, psychological risk factors (N=169). | Age, sex, and BMI. | - No/low support | - | - | - | - | 1.00 OR | - |
| | | | - Social support | - | - | - | - | 0.54 OR | 0.32 – 0.90 |

| | | | | | | | | | |
|---|---|---|---|---|---|---|---|---|---|
| Melloh<br>2013 | *Social support*: Social support was assessed with Caplan, Cobb, French, et al.'s scale combining score of 6–30 points with higher scores expressing higher social support on a 5-point scale (N=195). | Age, sex, and BMI | - No/low support<br>- Social support | -<br>- | -<br>- | -<br>- | -<br>- | 1.00 OR<br>1.78 OR | -<br>1.20 – 2.66 |

## Job stress

| | | | | | | | | | |
|---|---|---|---|---|---|---|---|---|---|
| Jørgensen<br>2013 | *Mental stress at work*: Was measured by the question: "do you feel under mental stress when performing your job/during leisure time?" (N=3,823). | Age. | - Seldom<br>- Regularly | 1.00 HR<br>0.64 HR | -<br>0.32 – 1.26 | -<br>- | -<br>- | -<br>- | -<br>- |
| Matsudaira<br>2015 | *Interpersonal stress*: Was assessed with the BJSQ on a five-point scale and dichotomised (N=171). | None. | - Not stressed<br>- Stressed | -<br>- | -<br>- | -<br>- | -<br>- | 1.00 OR<br>1.15 OR | -<br>0.49 – 2.68 |
| Matsudaira<br>2014 | *Interpersonal stress*: Was assessed with the BJSQ on a five-point scale and dichotomised (N=1,675). | Age, sex, obesity, smoking, education, and manual materials handling at work. | - No stress<br>- Stress | -<br>- | -<br>- | -<br>- | -<br>- | 1.00 OR<br>1.96 OR | -<br>1.00 – 3.82 |
| Matsudaira<br>2019 | *Interpersonal stress*: Was assessed with the CUPID-Questionnaire (N=197). | None. | - No stress<br>- Stressed | -<br>- | -<br>- | -<br>- | -<br>- | 1.00 OR<br>0.55 OR | -<br>0.26 – 1.15 |
| Melloh<br>2013 | *Stress*: Single-sided physical stress was assessed using the JCQ and measured on a seven-point scale (N=168, 38 with persistent LBP and 130 with non-persistent LBP). | Age, sex, and BMI. | - No/low stress<br>- Stressed | -<br>- | -<br>- | -<br>- | -<br>- | 1.00 OR<br>1.03 OR | -<br>0.43 – 2.51 |
| Seyedmehdi<br>2016 | *Stress*: Job stress was assessed with the JCQ on a four-point scale and dichotomised (N=511). | Age, Job experience, smoking, educational level, BMI, heavy physical load, and general health. | - Low<br>- High | -<br>- | -<br>- | -<br>- | -<br>- | 1.00 OR<br>1.67 OR | -<br>1.13 – 2.46 |
| Sørensen<br>2011 | *Mental stress at work*: Was measured by the question: "do you feel under mental stress when performing your job/during leisure time?" (N=3,823). | Age | - Seldom<br>- Regularly | 1.00 HR<br>0.64 HR | -<br>0.32 – 1.26 | -<br>- | -<br>- | -<br>- | -<br>- |

## Satisfaction

| | | | | | | | | | |
|---|---|---|---|---|---|---|---|---|---|
| Aghilinejad<br>2015 | *Satisfaction*: Was assessed using the MUSIC-questionnaire and measured by 4 questions on a five-point scale and dichotomised (N=185, 49 cases and 136 controls). | Age, BMI, smoking, LBP history, family LBP history, education, shift working, job type, other physical and psychosocial factors. | - Low<br>- High | 1.000 OR<br>0.501 OR | -<br>0.22 – 1.16 | -<br>- | -<br>- | -<br>- | -<br>- |

| | | | | | | | | | |
|---|---|---|---|---|---|---|---|---|---|
| Latza 2002 | *Satisfaction with own achievements at work*: Was measured by the question “I am satisfied with my achievements at work” using a 5-point Likert scale (without chronic LBP N=404 at baseline and all workers N= 488). | Age. | Without chronic LBP at baseline | | | | | | |
| | | | - Low | 1.00 PR | - | - | - | - | - |
| | | | - Medium | 1.53 PR | 0.55 – 4.23 | - | - | - | - |
| | | | - High | 1.85 PR | 0.67 – 5.01 | - | - | - | - |
| | | | All workers | | | | | | |
| | | | - Low | 1.00 PR | - | - | - | - | - |
| | | | - Medium | 1.67 PR | 0.88 – 3.13 | - | - | - | - |
| | | | - High | 2.07 PR | 1.10 – 3.88 | - | - | - | - |
| Matsudaira 2014 | *Job satisfaction*: Was assessed with the BJSQ on a five-point scale and dichotomised (N=1,675). | Age, sex, obesity, smoking, education, manual materials handling at work, somatic symptoms, and family history of LBP with disability. | - Satisfied | - | - | - | - | 1.00 OR | - |
| | | | - Not satisfied | - | - | - | - | 2.03 OR | 1.01 – 4.07 |
| Melloh 2013 | *Satisfaction*: Job satisfaction was assessed with the JCQ on a seven-point scale (N=168, 38 with persistent LBP and 130 with non-persistent LBP). | Age, sex, and BMI. | - Persistent group | - | - | - | - | 1.15 OR | 0.75 – 1.78 |
| Seidler 2003 | *Satisfaction with supervisors*: Was measured from 1 = very satisfied to 6 = very dissatisfied and classified by number of working years as 5 or 6 (N=175 controls and 128 cases). | Age, region, nationality, other diseases of the lumbar spine, and lifting/carrying combined with extreme forward bending. | - 0 years | 1.0 OR | - | - | - | - | - |
| | | | - >0 - <10 years | 1.1 OR | 0.6 – 2.1 | - | - | - | - |
| | | | - ≥10 | 1.1 OR | 0.3 – 4.0 | - | - | - | - |
| | *Satisfaction with workmates*: Was measured from 1 = very satisfied to 6 = very dissatisfied and classified by number of working years as 5 or 6 (N=175 controls and 125 cases). | Age, region, nationality, other diseases of the lumbar spine, and lifting/carrying combined with extreme forward bending. | - 0 years | 1.0 OR | - | - | - | - | - |
| | | | - >0 - <10 years | 2.7 OR | 0.8 – 9.3 | - | - | - | - |
| | | | - ≥10 | - | - | - | - | - | - |
| Tubach 2004 | *Job satisfaction*: Was measured with an analogic visual scale and divided into tertiles (N=475). | Sex, carrying loads, driving, home repair and renovation activity, visit to a medical practitioner for low back pain, sick leave, psychosomatic score, depression, long lasting LBP in 1991, and sciatica in 1990. | - High | - | - | - | - | 1.00 OR | - |
| | | | - Intermediate | - | - | - | - | 1.52 OR | 0.90 – 2.58 |
| | | | - Low | - | - | - | - | 1.13 OR | 0.71 – 1.78 |

## Other

| | | | | | | | | | |
|---|---|---|---|---|---|---|---|---|---|
| Ahsan 2013 | Less job satisfaction or stress at work. | Matched on age, sex, and area of residence. | - No | - | - | - | - | 1.00 OR | - |
| | | | - Yes | - | - | - | - | 2.45 OR | NS |
| Esquirol 2017 | *Repetitive work:* Repetitive work under time pressure were categorised into three classes according to exposure (N=1,130 for incidence and 430 for persistence). | None. | Incidence chronic LBP: | | | | | | |
| | | | - Never | - | - | - | - | 1.00 OR | - |
| | | | - Former | - | - | - | - | 1.39 OR | 0.87 – 2.20 |
| | | | - Current | - | - | - | - | 1.25 OR | 0.78 – 2.01 |
| | | | Persistence chronic LBP: | | | | | | |
| | | | - Never | - | - | - | - | 1.00 OR | - |

| | | | | | | | | | |
|---|---|---|---|---|---|---|---|---|---|
| | | | - Former | - | - | - | - | 1.09 OR | 0.60 – 1.99 |
| | | | - Current | - | - | - | - | 0.86 OR | 0.44 – 1.68 |
| | *Communicating*: Difficulty communicating with colleagues (N=430 for persistence). | Age, sex, history of rheumatologically events, history of depression, leisure time physical activity (sport), leisure time activity (gardening), and job changes between instances of data collection. | Persistence chronic LBP: | | | | | | |
| | | | - No | - | - | - | - | 1.00 OR | - |
| | | | - Yes | - | - | - | - | 1.45 OR | 0.87 – 2.42 |
| | *Recognition*: Work recognition was dichotomised (N=1,130 for incidence). | Sex, age, history of rheumatologically events., BMI, number of different jobs held, job changes, productivity-related income, and work recognition | Incidence chronic LBP: | | | | | | |
| | | | - Yes | - | - | - | - | 1.00 OR | - |
| | | | - No | - | - | - | - | 1.76 OR | 1.21 – 2.56 |
| Gold 2017 | *Work-family imbalance:* Was assessed with the sum of three items regarding tiredness after work, so much work it takes away personal interests, and preoccupation with work while being home (N=228). | No information is given. | - Yes | - | - | - | - | 1.82 OR | 1.12 – 2.98 |
| Halonen 2018 | *Effort-reward*: Effort-reward imbalance (ERI) perception was assessed using the ERI-Questionnaire and measured by an Effort-reward ratio and dichotomised (N=1,845). | Age, sex, number of pseudo-trials, marital status, socioeconomic status, and physically strenuous work. | LBP after onset of ERI: | | | | | | |
| | | | - Affecting | - | - | - | - | 1.21 RR | 0.97 – 1.50 |
| | | | - Any | - | - | - | - | 1.05 RR | 0.85 – 1.29 |
| Latza 2002 | *Monotonous work*: Was measured by the question "Altogether, my work is uniform" using a 5-point Likert scale (without chronic LBP N=404 at baseline and all workers N= 488). | Age. | Without chronic LBP at baseline | | | | | | |
| | | | - Low | 1.00 PR | - | - | - | - | - |
| | | | - Medium | 1.39 PR | 0.58 – 3.36 | - | - | - | - |
| | | | - High | 1.40 PR | 0.59 – 3.31 | - | - | - | - |
| | | | All workers | | | | | | |
| | | | - Low | 1.00 PR | - | - | - | - | - |
| | | | - Medium | 1.46 PR | 0.82 – 2.60 | - | - | - | - |
| | | | - High | 1.50 PR | 0.86 – 2.62 | - | - | - | - |
| | *Time pressure*: Was measured by the question "I am under time pressure" using a 5-point Likert scale (without chronic LBP N=404 at baseline and all workers N= 488). | Age. | Without chronic LBP at baseline | | | | | | |
| | | | - Low | 1.00 PR | - | - | - | - | - |
| | | | - Medium | 7.43 PR | 1.70 – 32.6 | - | - | - | - |
| | | | - High | 6.30 PR | 1.41 – 28.2 | - | - | - | - |
| | | | All workers | | | | | | |
| | | | - Low | 1.00 PR | - | - | - | - | - |
| | | | - Medium | 1.63 PR | 0.87 – 3.06 | - | - | - | - |
| | | | - High | 1.70 PR | 0.92 – 3.15 | - | - | - | - |
| Matsudaira 2019 | *Inadequate breaks at work:* Assessed using the JCQ (N=197). | | - Yes | - | - | - | - | 0.71 OR | 0.30 – 1.67 |

| | | | | | | | | | |
|---|---|---|---|---|---|---|---|---|---|
| | *Dissatisfied with job*: Was assessed with the CUPID-Questionnaire (N=198). | None. | - Not dissatisfied<br>- Dissatisfied | -<br>- | -<br>- | -<br>- | -<br>- | 1.00 OR<br>1.14 OR | -<br>0.52 – 2.50 |
| Matsudaira 2015 | *Utilization of skills and expertise:* Assessed using the JCQ (N=140). | None. | - Utilization of skills and expertise<br>- No utilization of skills and expertise | -<br>- | -<br>- | -<br>- | -<br>- | 1.00 OR<br>1.59 OR | -<br>0.66 – 3.85 |
| | *Job Fitness:* Feeling fit for the job (N=121). | None. | - Feeling fit<br>- Not feeling fit | -<br>- | -<br>- | -<br>- | -<br>- | 1.00 OR<br>2.04 OR | -<br>0.91 – 4.60 |
| | *Reward*: Reward to work was assessed with the BJSQ on a five-point scale and dichotomised (N=171). | Anxiety and daily-life satisfaction. | - Feel rewarded<br>- Not feeling rewarded | -<br>- | -<br>- | -<br>- | -<br>- | 1.00 OR<br>3.59 OR | -<br>1.57 – 8.20 |
| | *Mental workload*: The quantitative aspect was assessed with the BJSQ on a five-point scale and dichotomised (N=170). | None. | - Not stressed<br>- Stressed | -<br>- | -<br>- | -<br>- | -<br>- | 1.00 OR<br>1.08 OR | -<br>0.47 – 2.46 |
| | *Mental workload*: The qualitative aspect was assessed with the BJSQ on a five-point scale and dichotomised (N=170). | None. | - Not stressed<br>- Stressed | -<br>- | -<br>- | -<br>- | -<br>- | 1.00 OR<br>0.63 OR | -<br>0.28 – 1.42 |
| | *Physical workload*: Was assessed with the BJSQ on a five-point scale and dichotomised (N=171). | None. | - Not stressed<br>- Stressed | -<br>- | -<br>- | -<br>- | -<br>- | 1.00 OR<br>1.62 OR | -<br>0.70 – 3.73 |
| | *Environment stress*: Was assessed with the BJSQ on a five-point scale and dichotomised (N=171). | None. | - Not stressed<br>- Stressed | -<br>- | -<br>- | -<br>- | -<br>- | 1.00 OR<br>1.95 OR | -<br>0.87 – 4.38 |
| Matsudaira 2014 | *Physical workload*: Was assessed with the BJSQ on a five-point scale and dichotomised (N=1,675). | Age, sex, obesity, smoking, education, and manual materials handling at work. | - No stress<br>- Stress | -<br>- | -<br>- | -<br>- | -<br>- | 1.00 OR<br>1.53 OR | -<br>0.70 – 3.33 |
| Melloh 2013 | *Work absenteeism:* Assessed using the JCQ (N=168, 38 with persistent LBP and 130 with non-persistent LBP). | Age, sex, and BMI. | - Yes | - | - | - | - | 1.00 OR | 0.99 – 1.01 |
| | *Resigned attitude towards the job:* Assessed using the JCQ (N=168, 38 with persistent LBP and 130 with non-persistent LBP). | Age, sex, and BMI. | - Yes | - | - | - | - | 1.48 OR | 0.99 – 2.20 |
| | *Uncertainty:* Assessed using the JCQ (N=168, 38 with persistent LBP and 130 with non-persistent LBP). | Age, sex, and BMI. | - Yes | - | - | - | - | 0.92 OR | 0.47 – 1.83 |
| | *Work organisational problems:* Assessed using the JCQ | Age, sex, and BMI. | - Yes | - | - | - | - | 0.63 OR | 0.30 – 1.34 |
| | *Work interruptions:* Assessed using the JCQ (N=168, 38 with persistent LBP and 130 with non-persistent LBP). | Age, sex, and BMI. | - Yes | - | - | - | - | 0.87 OR | 0.47 – 1.58 |

| | | | | | | | | | |
|---|---|---|---|---|---|---|---|---|---|
| | *Concentration requirements:* Assessed using the JCQ (N=168, 38 with persistent LBP and 130 with non-persistent LBP). | Age, sex, and BMI. | - Yes | - | - | - | - | 1.84 OR | 0.95 – 3.77 |
| | *Time pressure:* Assessed using the JCQ (N=168, 38 with persistent LBP and 130 with non-persistent LBP). | Age, sex, and BMI. | - Yes | - | - | - | - | 0.76 OR | 0.40 – 1.47 |
| | *Emotional suppression:* Assessed using the JCQ (N=168, 38 with persistent LBP and 130 with non-persistent LBP). | Age, sex, and BMI. | - Yes | - | - | - | - | 1.09 OR | 0.67 – 1.75 |
| Melloh 2013 | *Resigned attitude towards the job*: Were assessed by questionnaires based on the recommendations of a multinational musculoskeletal cohort study addressing occupational, psychological risk factors (N=169). | Age, sex, BMI, and social support. | Resigned attitude towards the job | - | - | - | - | 1.83 OR | 1.23 – 2.72 |
| | *Job satisfaction*: Were assessed by questionnaires based on the recommendations of a multinational musculoskeletal cohort study addressing occupational, psychological risk factors (N=169). | Age, sex, and BMI. | Job satisfaction | - | - | - | - | 0.74 OR | 0.57 – 0.96 |
| | *Time pressure*: Were assessed by questionnaires based on the recommendations of a multinational musculoskeletal cohort study addressing occupational, psychological risk factors (N=169). | Age, sex, and BMI. | Time pressure | - | - | - | - | 1.20 OR | 0.83 – 1.75 |
| | *Uncertainty*: Were assessed by questionnaires based on the recommendations of a multinational musculoskeletal cohort study addressing occupational, psychological risk factors (N=169). | Age, sex, and BMI. | Uncertainty | - | - | - | - | 1.22 OR | 0.81 – 1.83 |
| | *Physically demanding work activities*: Were assessed by questionnaires based on the recommendations of a multinational musculoskeletal cohort study addressing occupational, psychological risk factors (N=169). | Age, sex, and BMI. | Physically demanding work activities | - | - | - | - | 0.90 OR | 0.39 – 2.04 |
| | *Ergonomics*: Were assessed by questionnaires based on the recommendations of a multinational musculoskeletal cohort study addressing occupational, psychological risk factors (N=169). | Age, sex, and BMI. | Ergonomics | - | - | - | - | 0.80 OR | 0.46 – 1.42 |
| Seidler 2003 | *Opportunities to use knowledge and skills*: Was measured from 1 = very often to 6 = very seldom and classified by number of working years as 5 or 6 (N=176 controls and 127 cases). | Age, region, nationality, other diseases of the lumbar spine, and lifting/carrying combined with extreme forward bending. | - 0 years<br>- >0 - <10 years<br>- ≥10 | 1.0 OR<br>1.6 OR<br>0.5 OR | -<br>0.9 – 2.9<br>0.1 – 2.2 | -<br>-<br>- | -<br>-<br>- | -<br>-<br>- | -<br>-<br>- |
| | *Psychic strain through contact with clients*: Was measured from 1 = very little to 6 = very much | Age, region, nationality, other diseases of the lumbar spine, | - 0 years<br>- >0 - <10 years | 1.0 OR<br>6.9 OR | -<br>1.2 – 40.1 | -<br>- | -<br>- | -<br>- | -<br>- |

| | | | | | | | | | |
|---|---|---|---|---|---|---|---|---|---|
| | and classified by number of working years with high degree of psychic strain as 5 or 6 (N=184 controls and 130 cases). | and lifting/carrying combined with extreme forward bending. | - ≥10 | - | - | - | - | - | - |
| | *Time pressure*: Was measured from 1 = very little to 6 = very much and classified by number of working years under high degree of time pressure as 5 or 6 (N=176 controls and 128 cases). | Age, region, nationality, other diseases of the lumbar spine, and lifting/carrying combined with extreme forward bending. | - 0 years<br>- >0 - <10 years<br>- ≥10 | 1.0 OR<br>1.3 OR<br>2.3 OR | -<br>0.7 – 2.8<br>1.1 – 4.8 | -<br>-<br>- | -<br>-<br>- | -<br>-<br>- | -<br>-<br>- |

Abbreviations: BJSQ = Brief Job Stress Questionnaire; BMI = body mass index; CUPID = Cultural and Psychosocial Influences on Disability; HR = Hazard Ratio; JCQ = Job Content Questionnaire; kg = kilogram; LDH = lumbar disc herniation; LDN = lumbar disc narrowing; Musculoskeletal Intervention Centre; NS = Not stated; OR = Odds Ratio; PR = Prevalence Ratio; RR = Relative Risk.

## **Appendix H**. Funnel plots

**Figure S1**. Job control

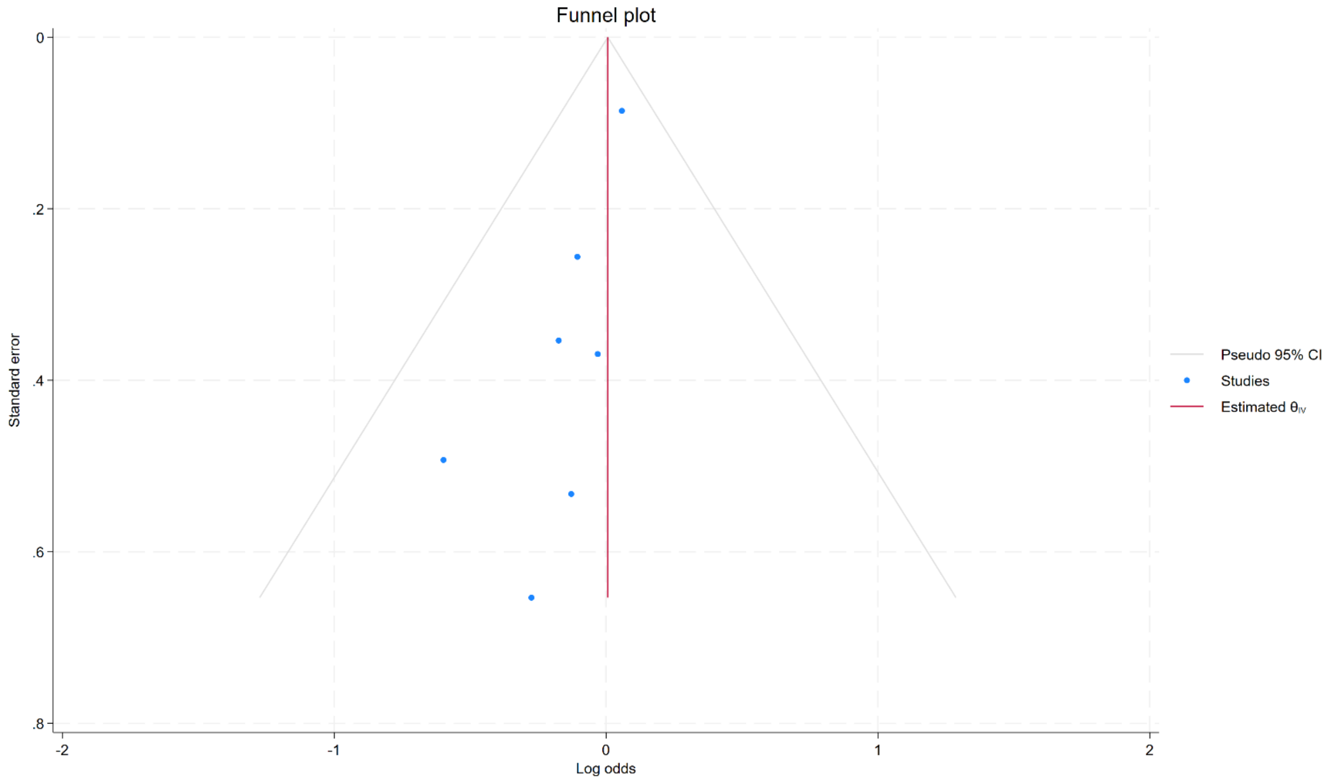


**Figure S2.** Job demand

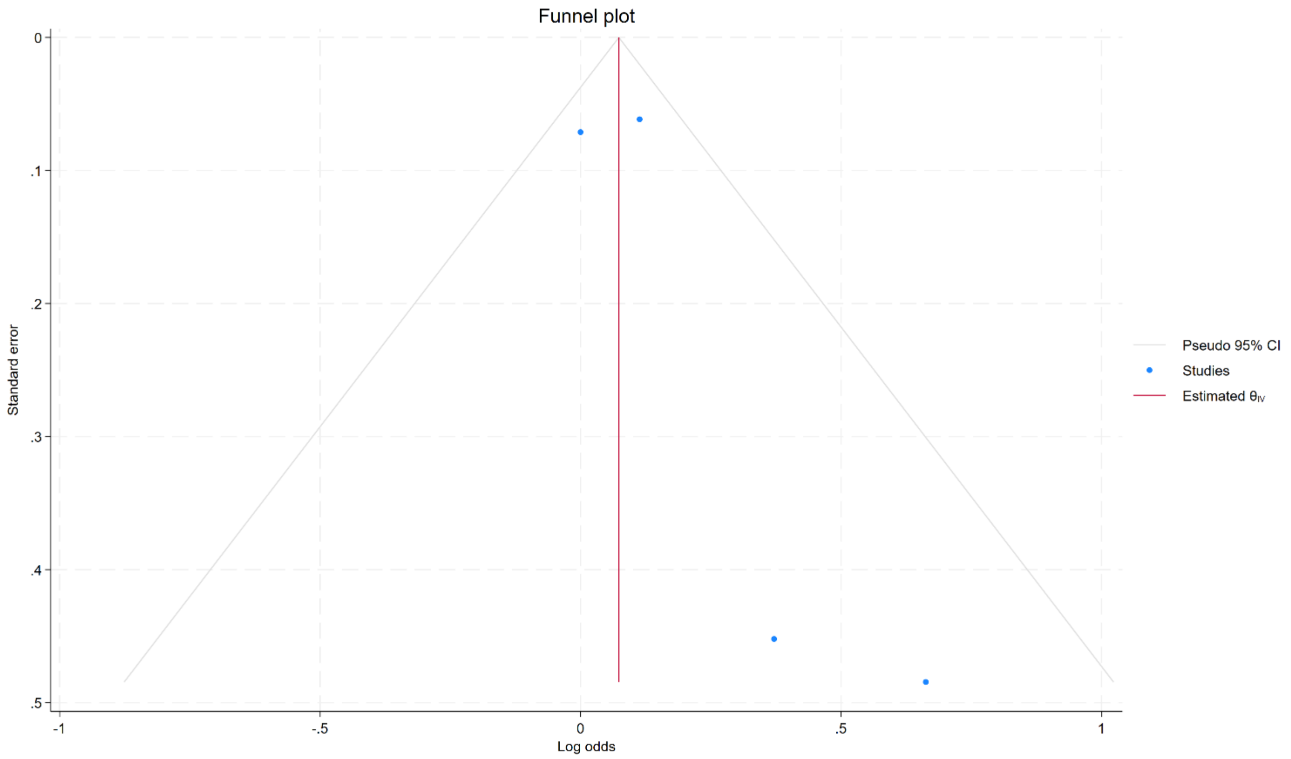

**Figure S3**. Job support

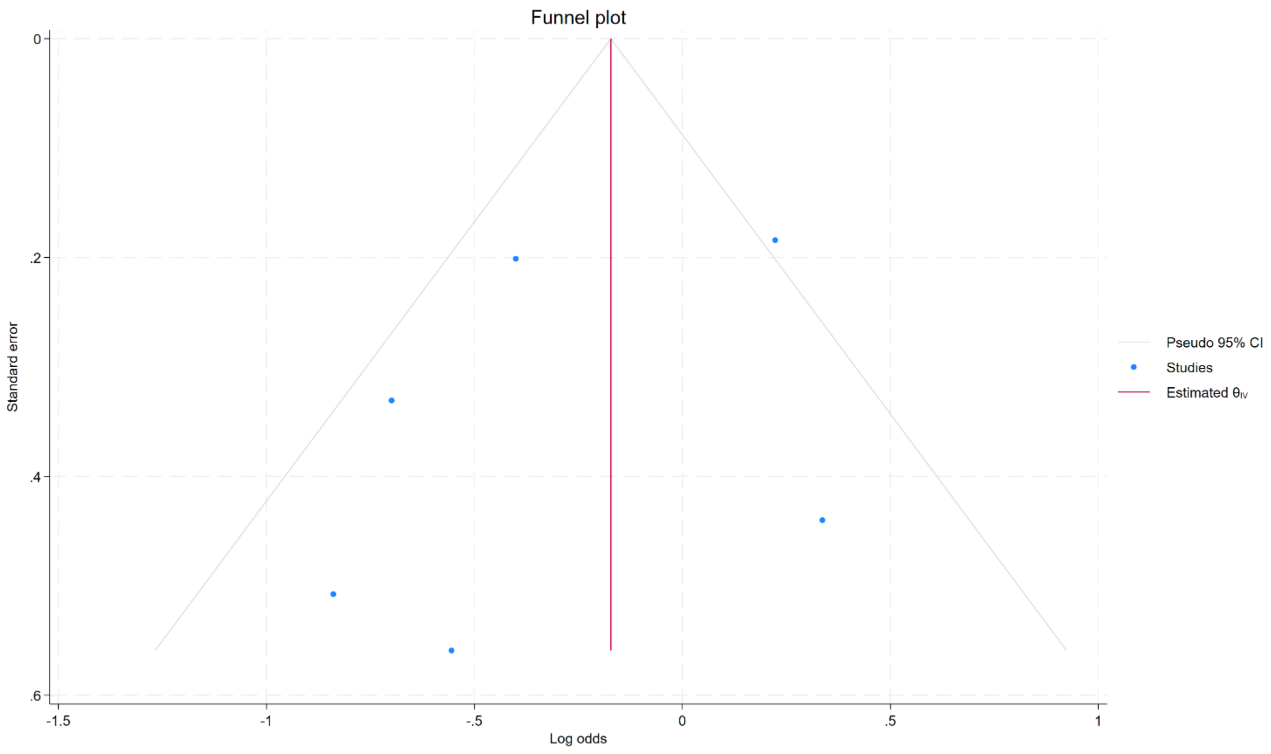


**Figure S4**. Job stress

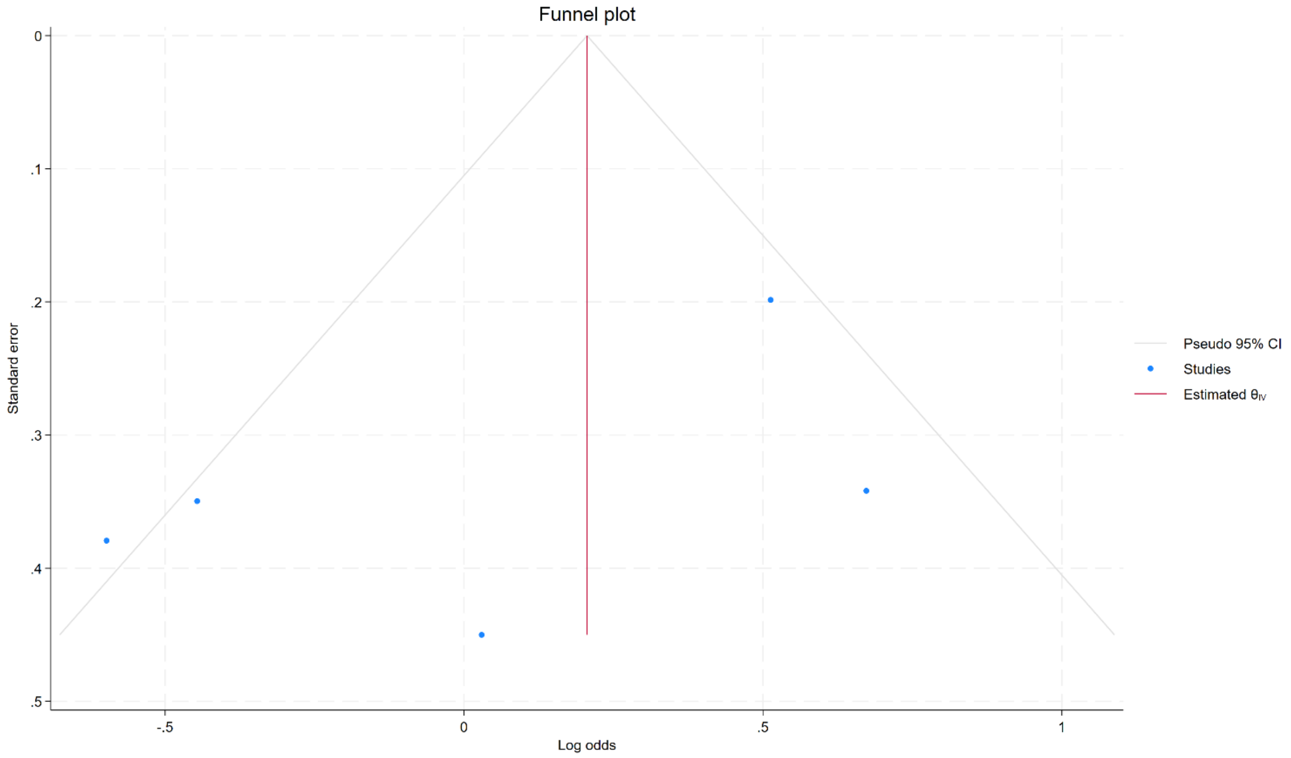

**Figure S5**. Job satisfaction

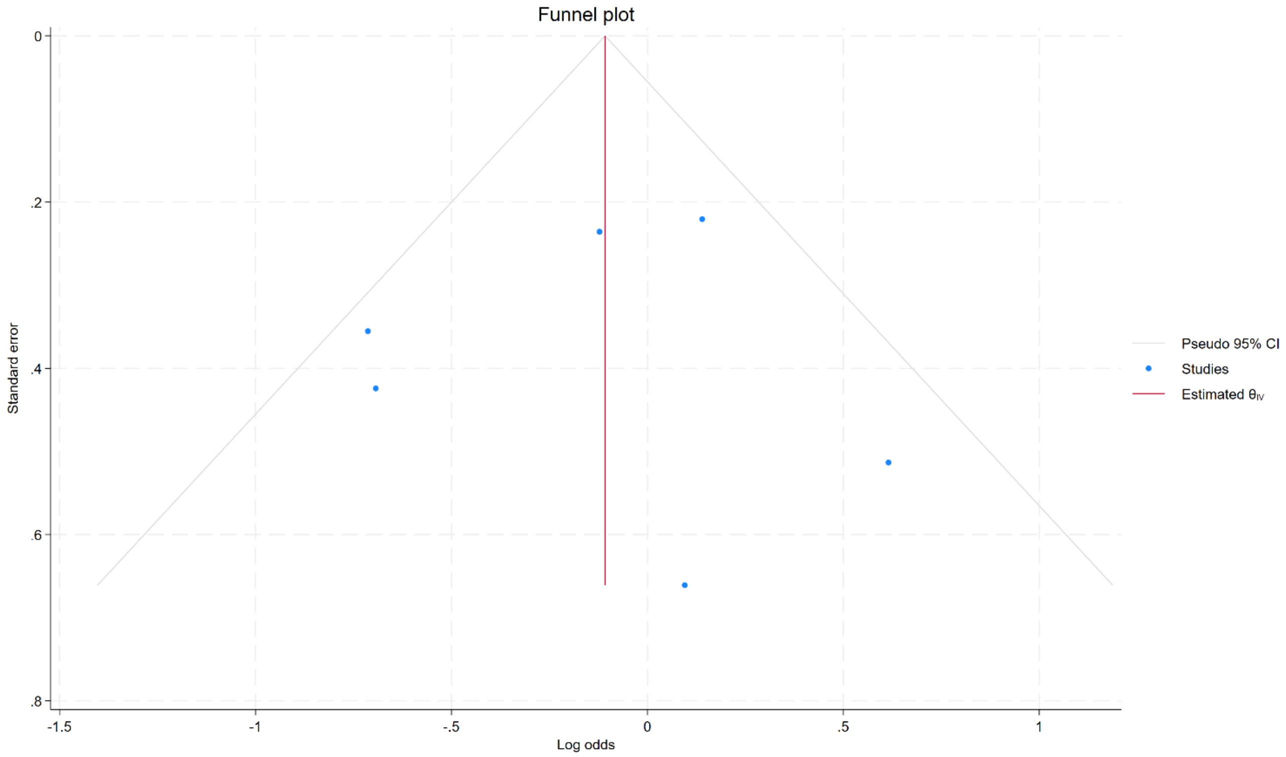

Supplement: Supplementary material [file SJWEH-50-329-S001.pdf]
